# Supplementary figures and images for: Nuclei segmentation of HE stained histopathological images based on feature global delivery connection network
Source: PLoS One. 2022 Sep 15;17(9):e0273682. doi: 10.1371/journal.pone.0273682 (PMC9477331; doi:10.1371/journal.pone.0273682)

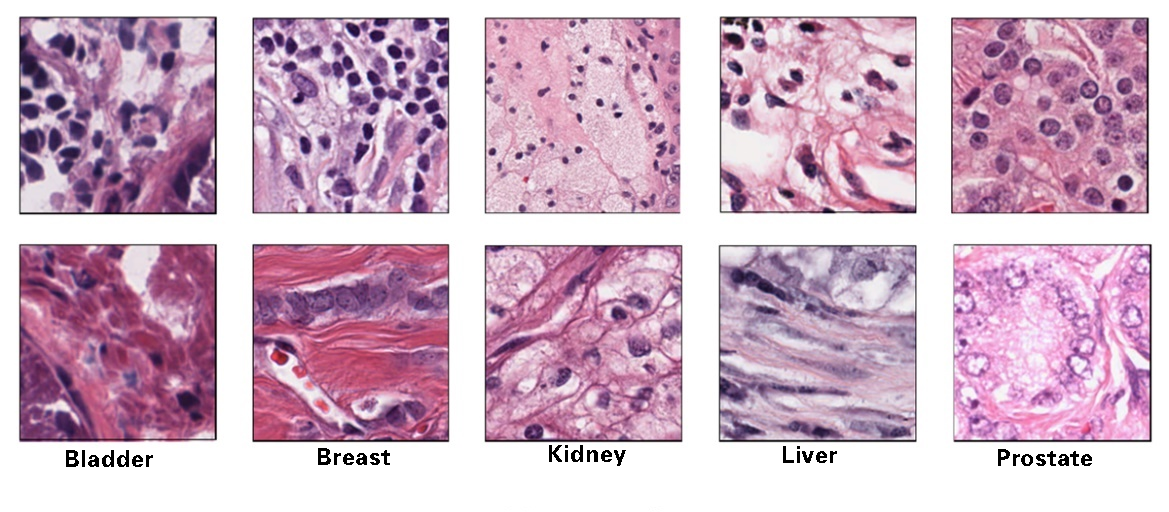

Supplement: S1 Fig — (ZIP) [file pone.0273682.s001.zip › Figure1.png]

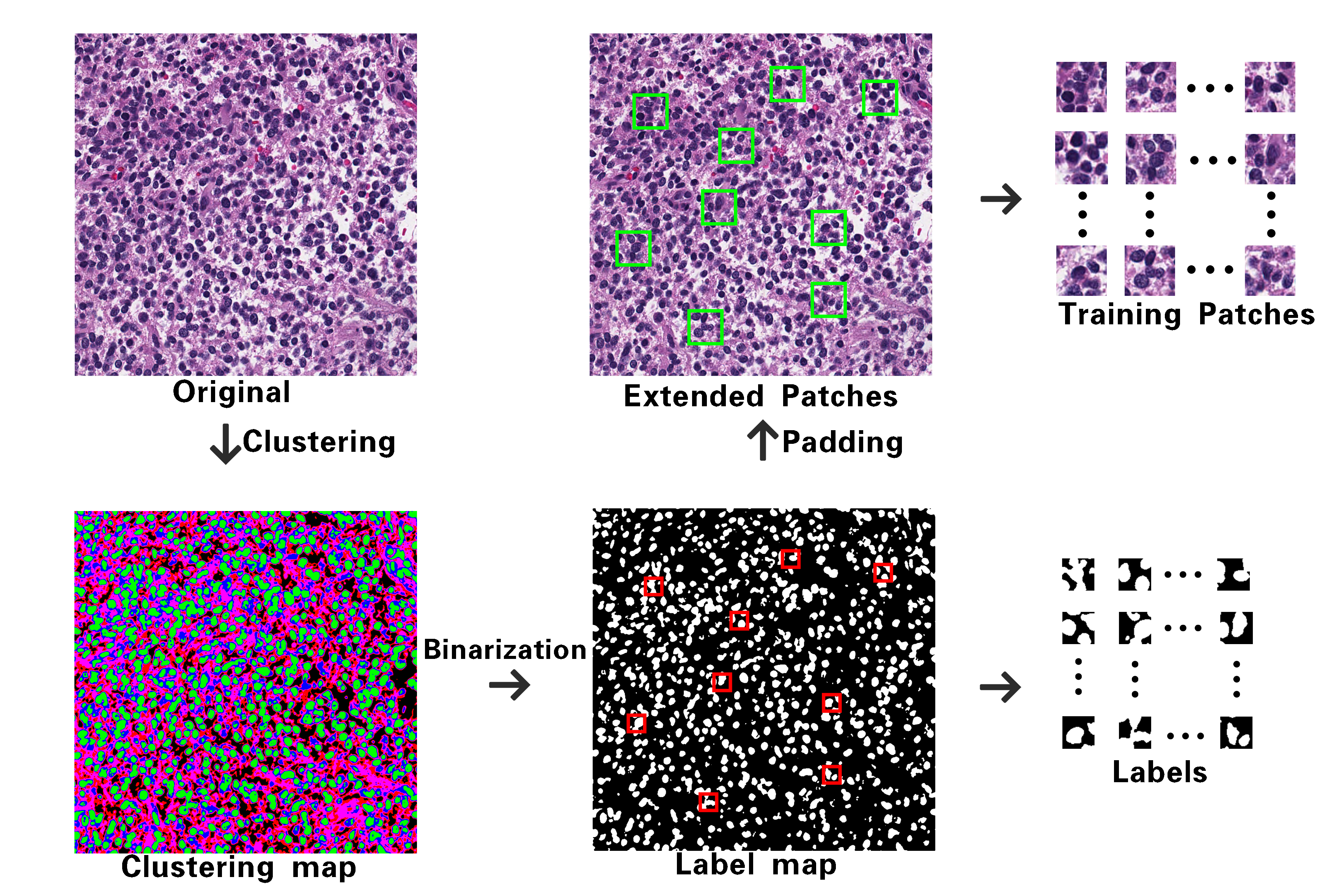

Supplement: S2 Fig — (ZIP) [file pone.0273682.s002.zip › Figure2.png]

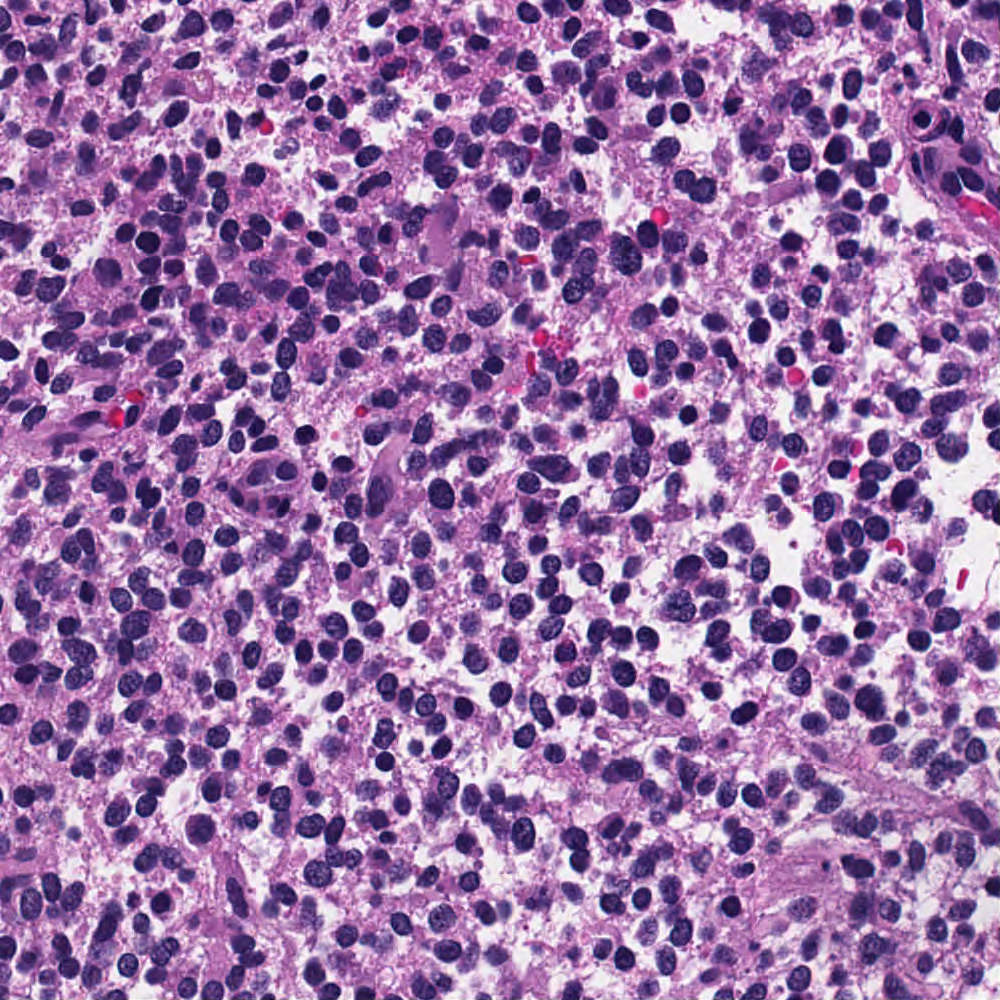

Supplement: S2 Fig — (ZIP) [file pone.0273682.s002.zip › Figure2_Ori.png]

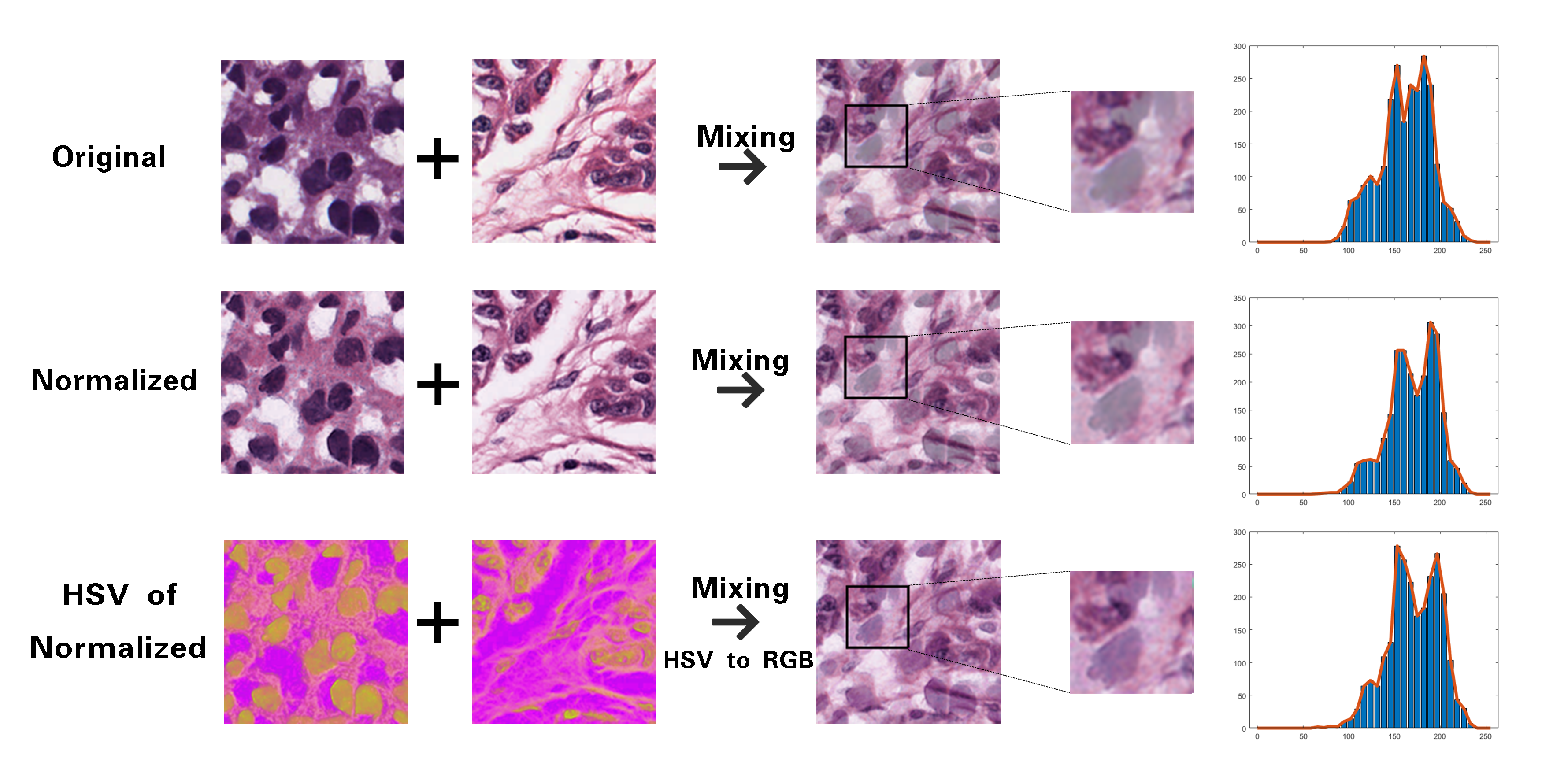

Supplement: S3 Fig — (ZIP) [file pone.0273682.s003.zip › Figure3.png]

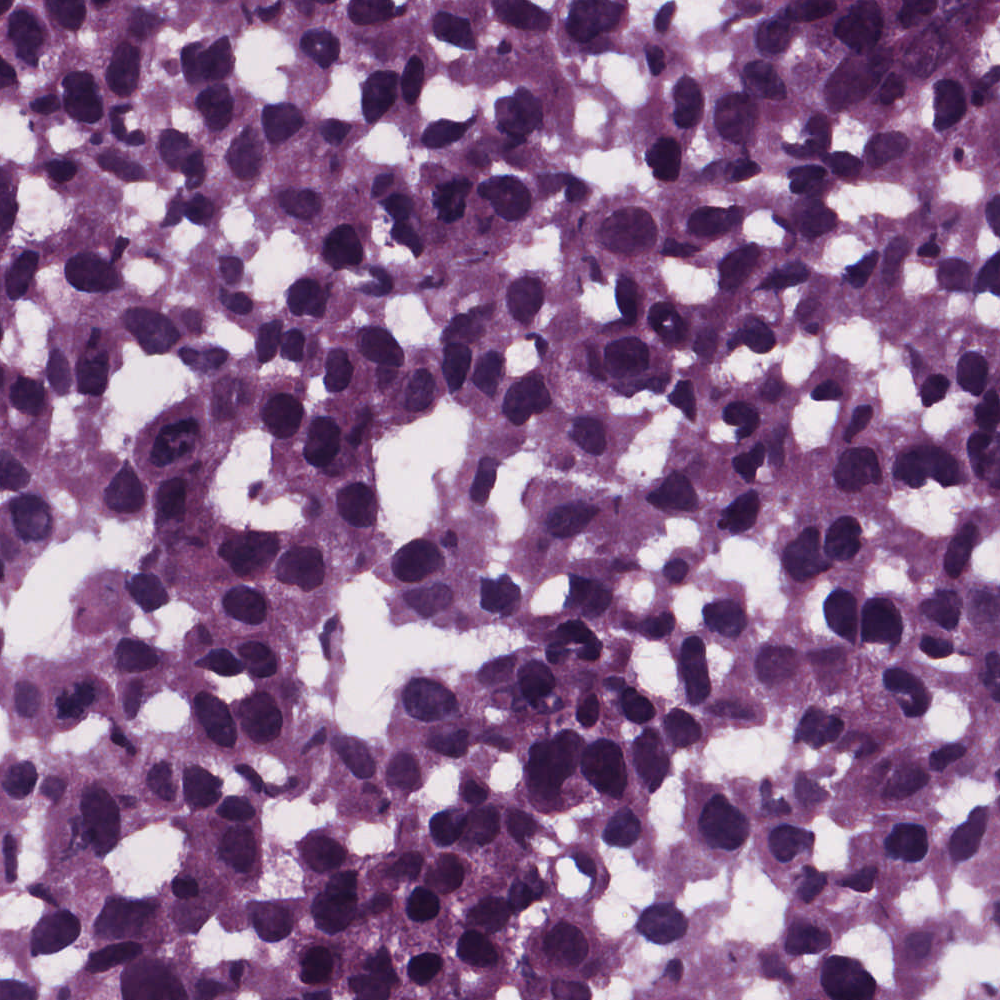

Supplement: S3 Fig — (ZIP) [file pone.0273682.s003.zip › Figure3_Ori.png]

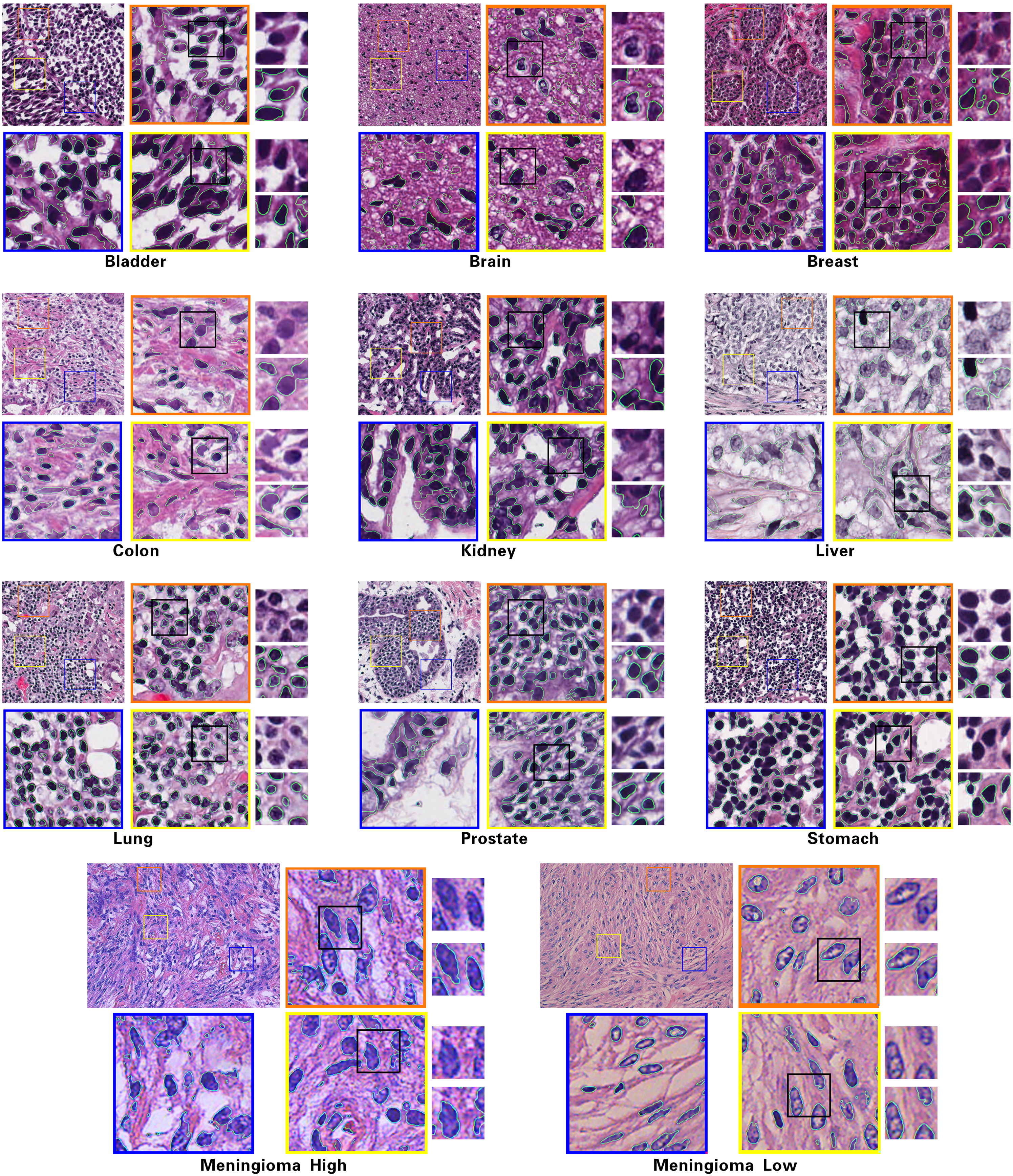

Supplement: S4 Fig — (ZIP) [file pone.0273682.s004.zip › Figure7.png]

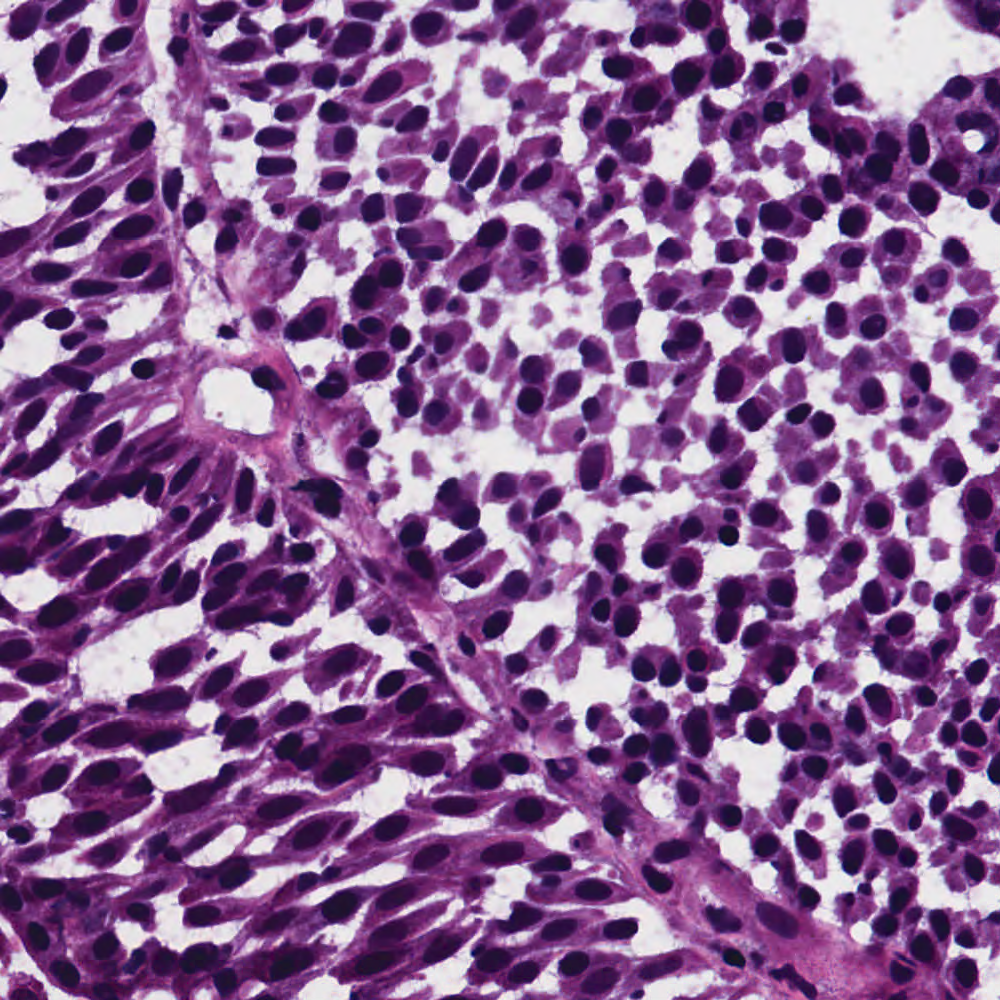

Supplement: S4 Fig — (ZIP) [file pone.0273682.s004.zip › Figure7_Ori_Bladdder.tif]

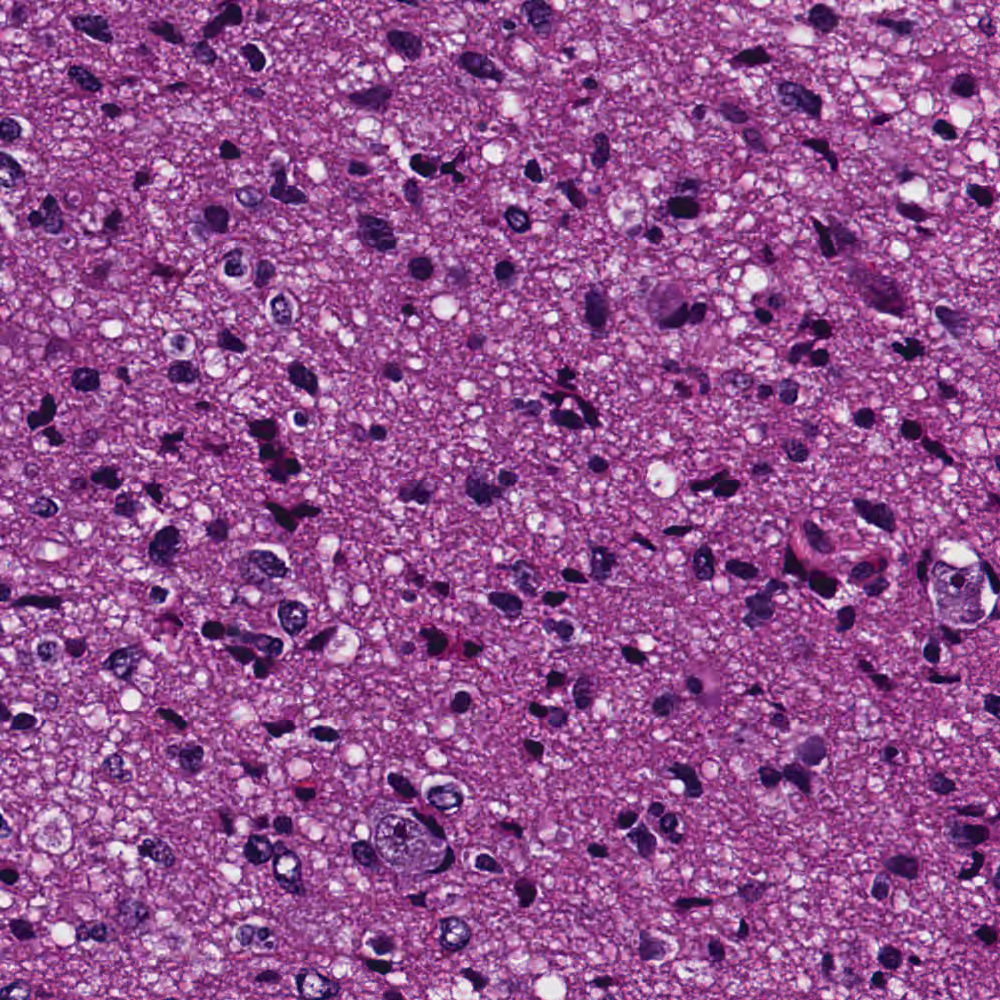

Supplement: S4 Fig — (ZIP) [file pone.0273682.s004.zip › Figure7_Ori_Brain.tif]

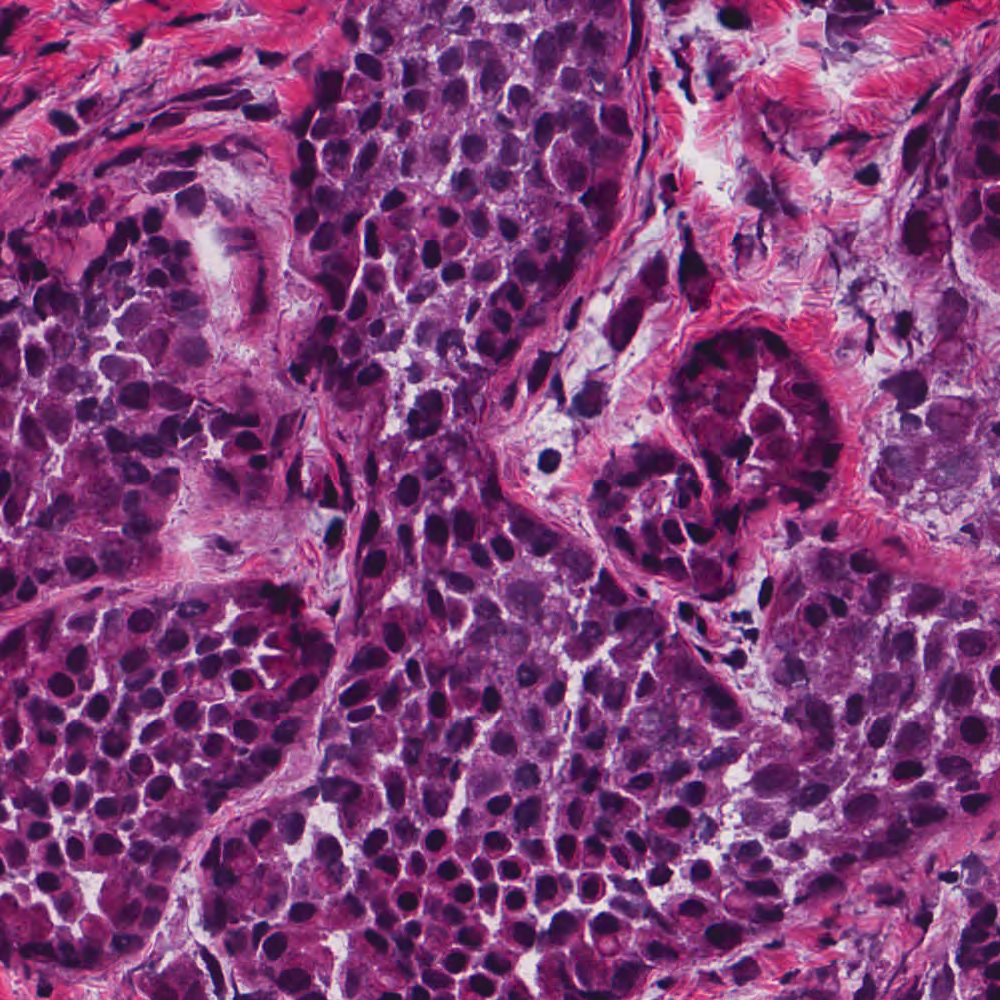

Supplement: S4 Fig — (ZIP) [file pone.0273682.s004.zip › Figure7_Ori_Breast.tif]

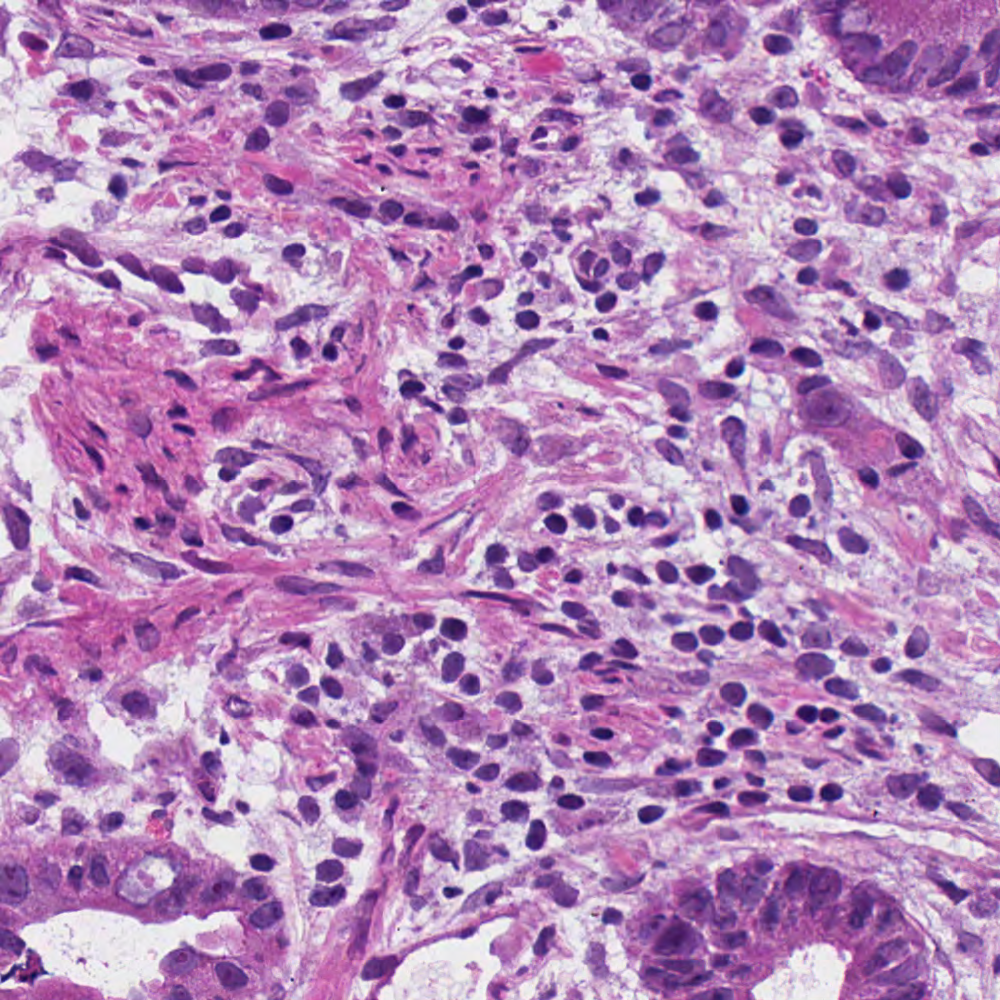

Supplement: S4 Fig — (ZIP) [file pone.0273682.s004.zip › Figure7_Ori_Colon.tif]

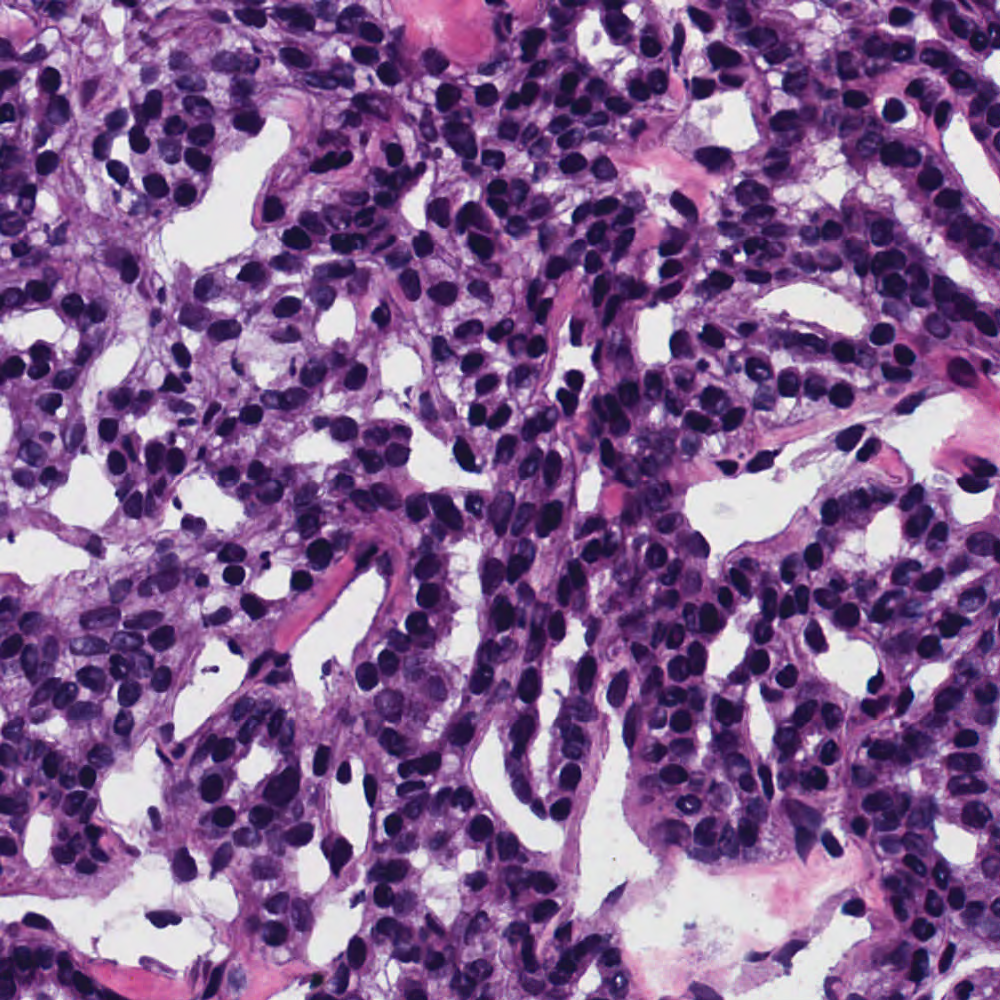

Supplement: S4 Fig — (ZIP) [file pone.0273682.s004.zip › Figure7_Ori_Kidney.tif]

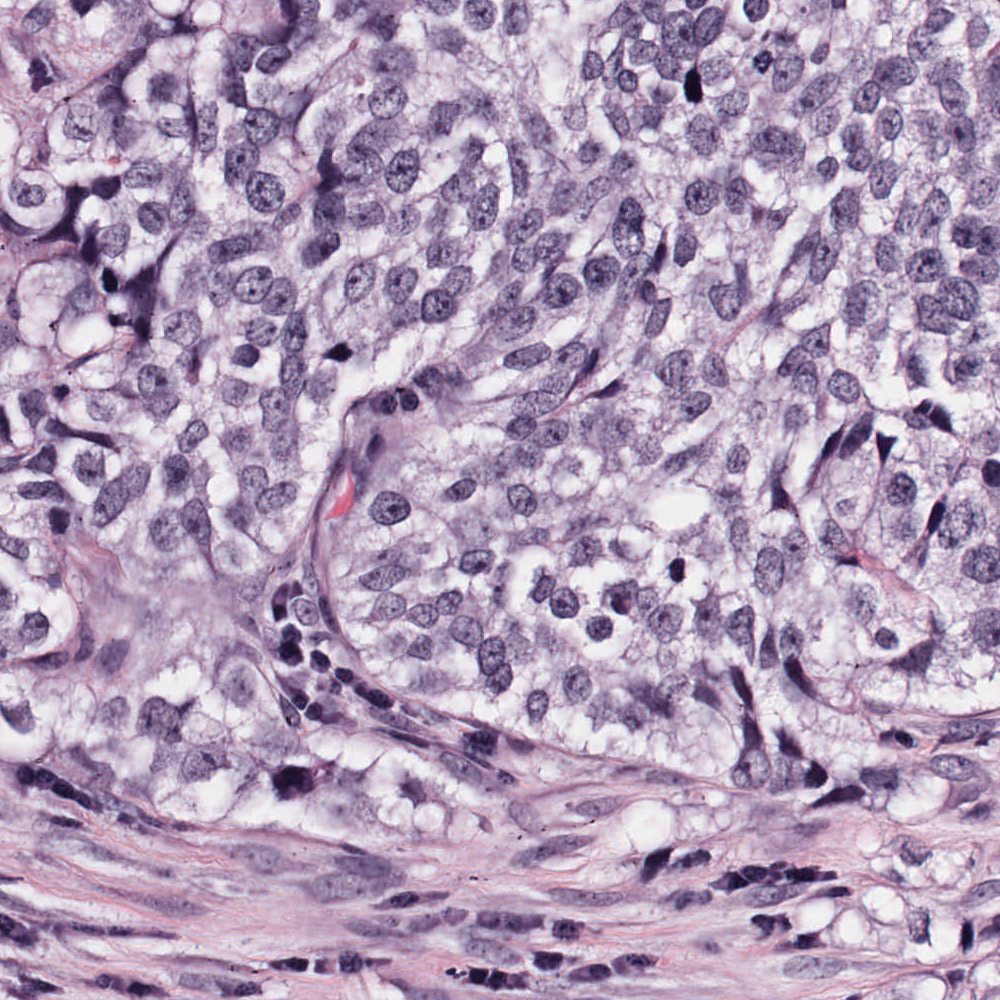

Supplement: S4 Fig — (ZIP) [file pone.0273682.s004.zip › Figure7_Ori_Liver.tif]

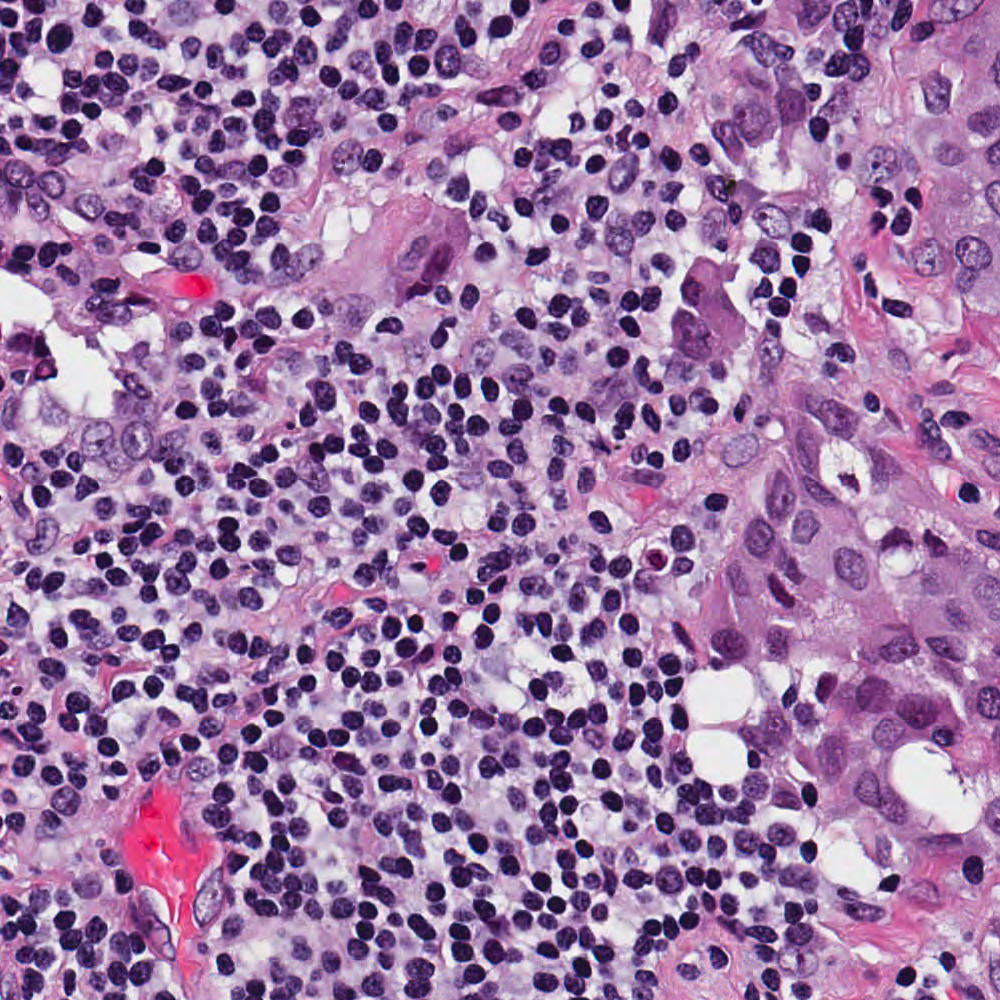

Supplement: S4 Fig — (ZIP) [file pone.0273682.s004.zip › Figure7_Ori_Lung.tif]

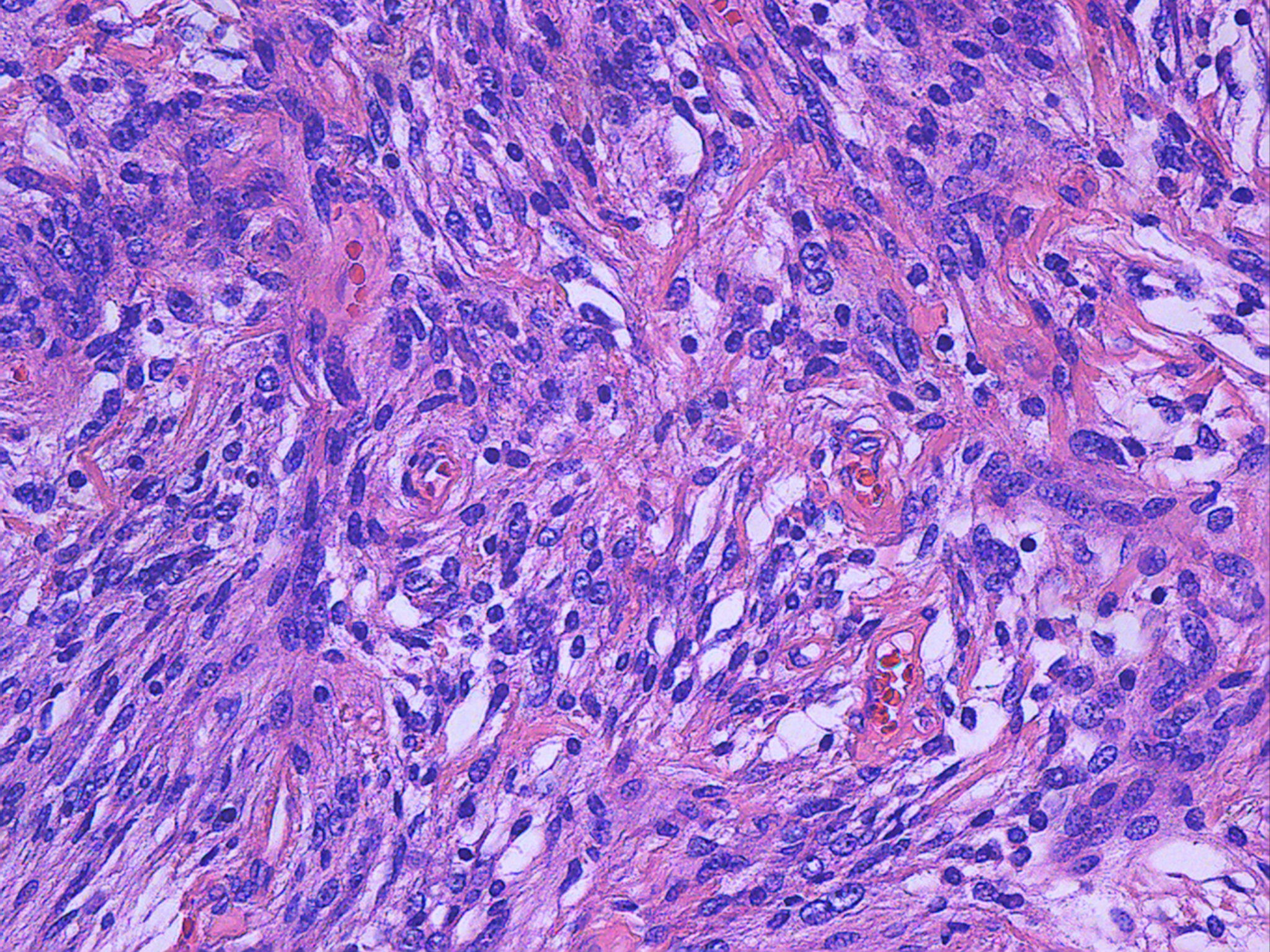

Supplement: S4 Fig — (ZIP) [file pone.0273682.s004.zip › Figure7_Ori_Meningioma1.tif]

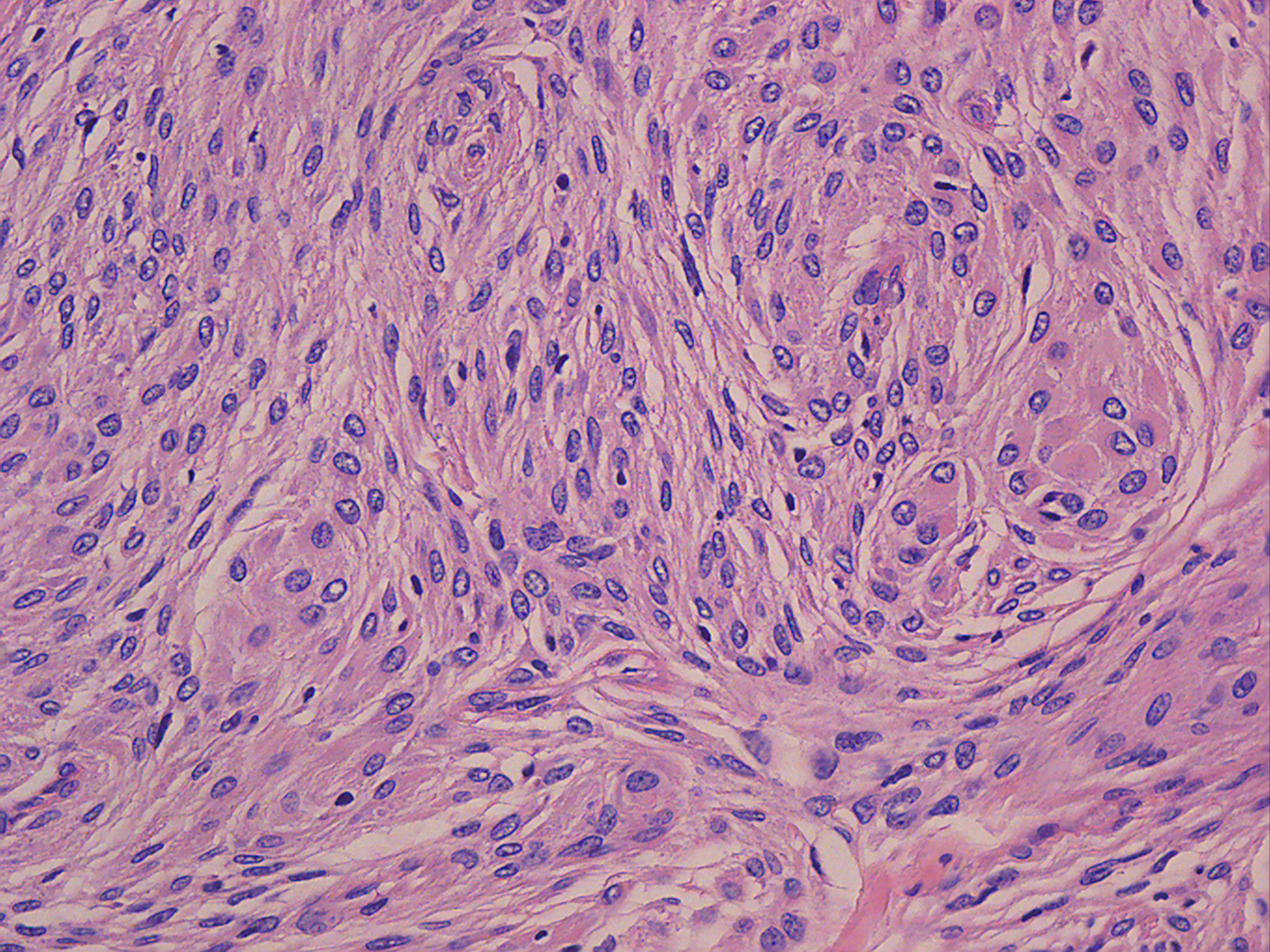

Supplement: S4 Fig — (ZIP) [file pone.0273682.s004.zip › Figure7_Ori_Meningioma2.tif]

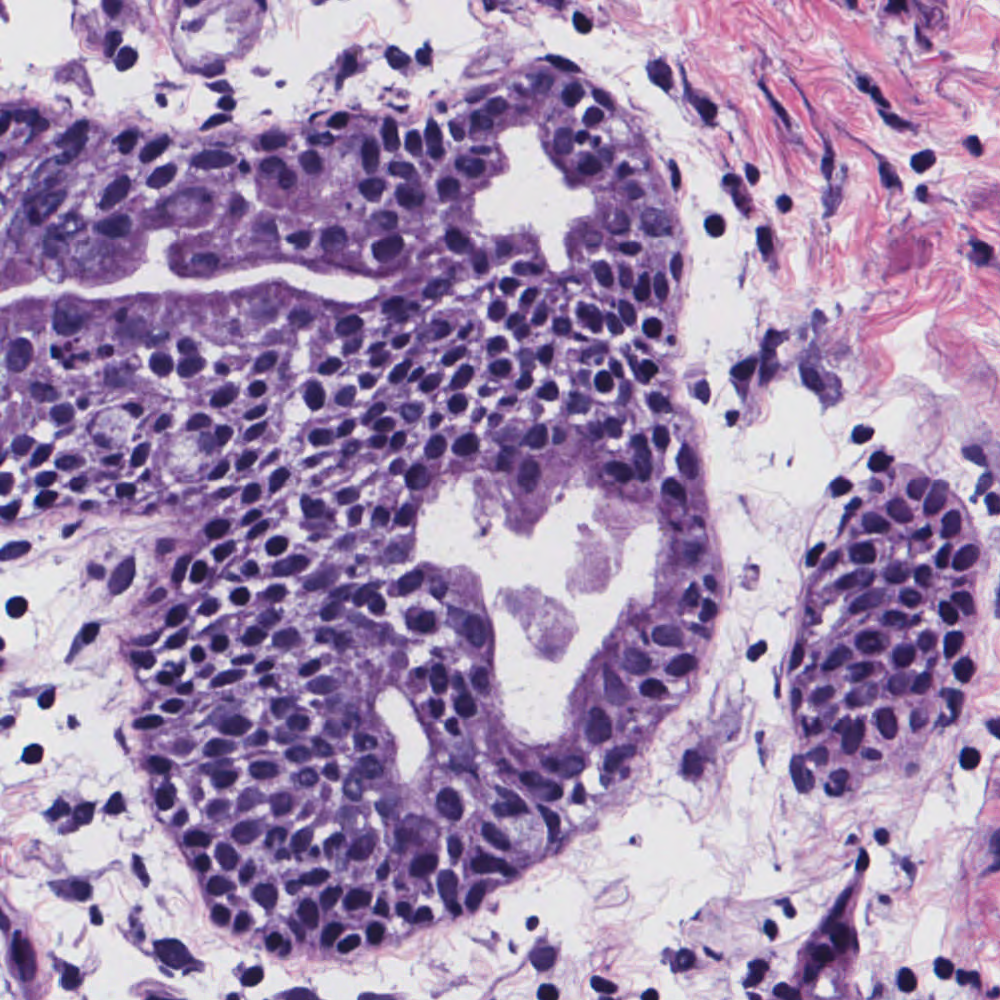

Supplement: S4 Fig — (ZIP) [file pone.0273682.s004.zip › Figure7_Ori_Prostate.tif]

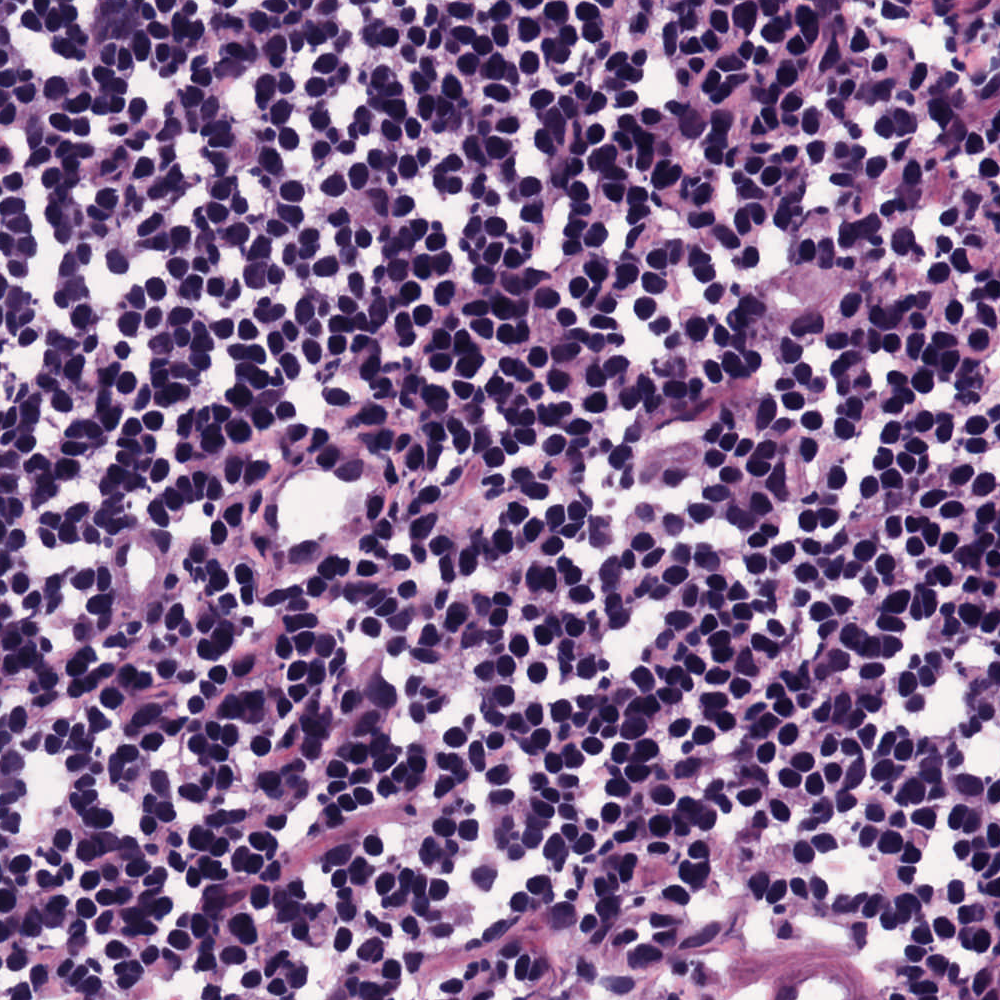

Supplement: S4 Fig — (ZIP) [file pone.0273682.s004.zip › Figure7_Ori_Stomach.tif]

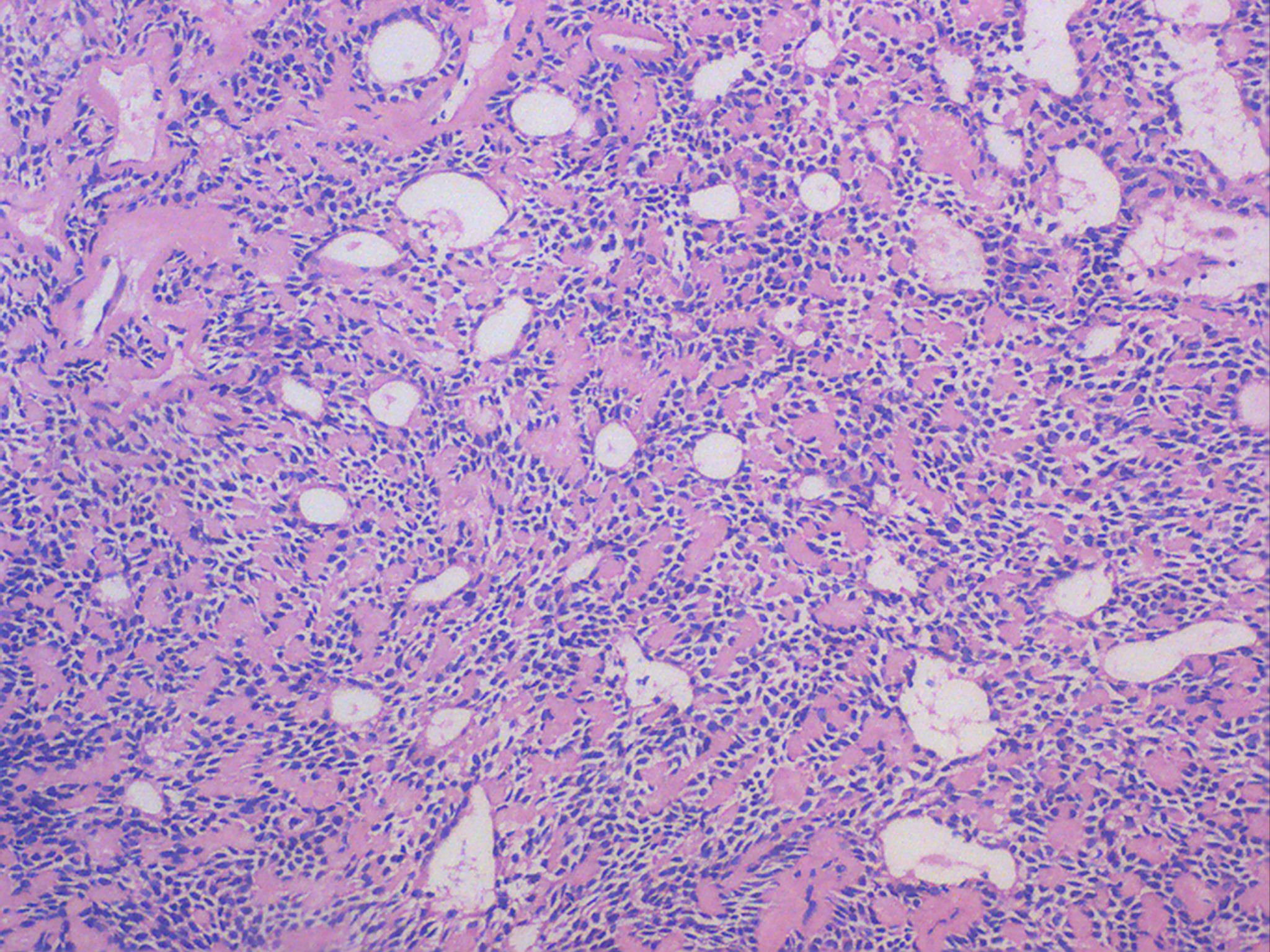

Supplement: S5 Fig — (ZIP) [file pone.0273682.s005.zip › 01.tif]

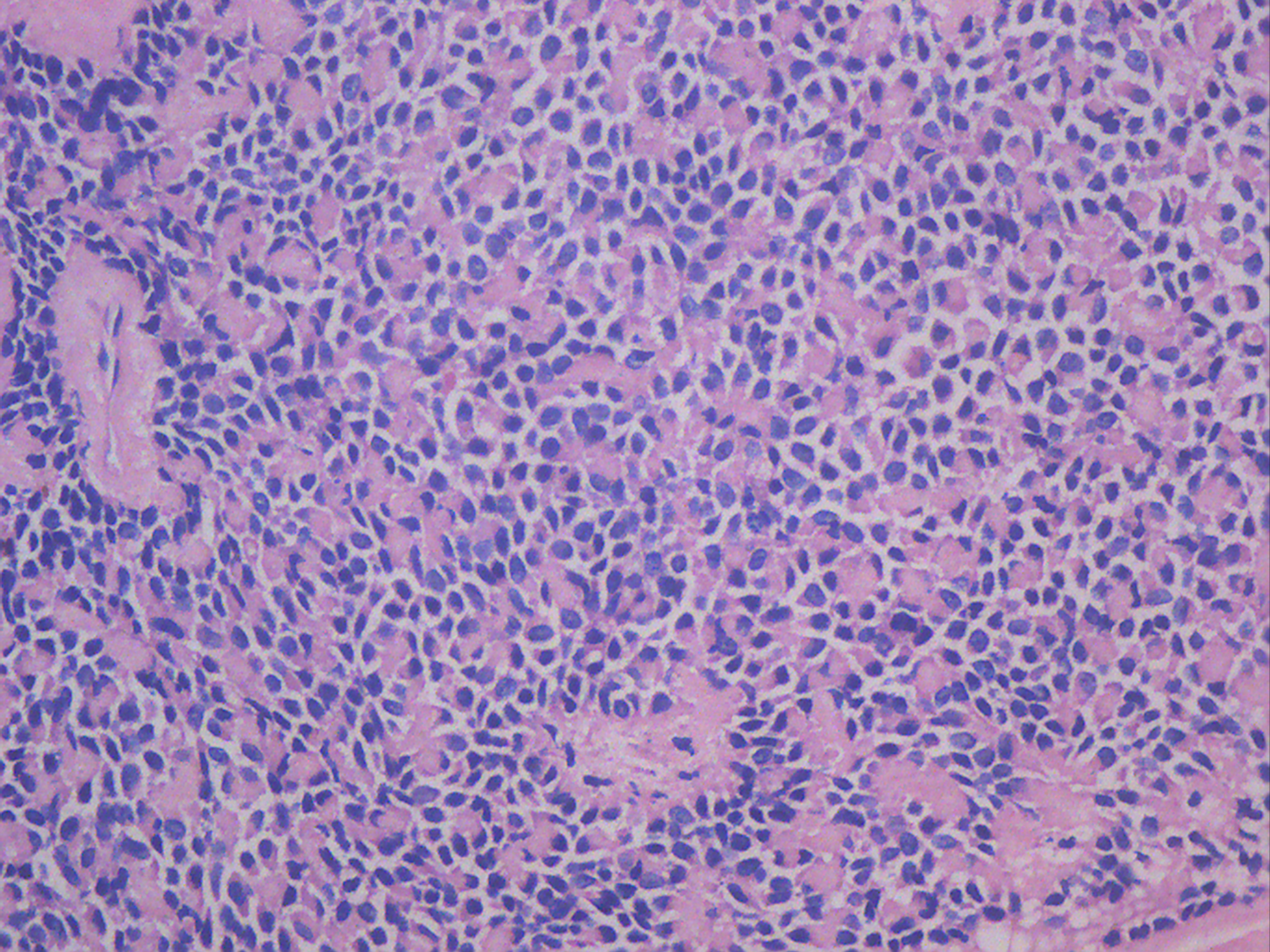

Supplement: S5 Fig — (ZIP) [file pone.0273682.s005.zip › 02.tif]

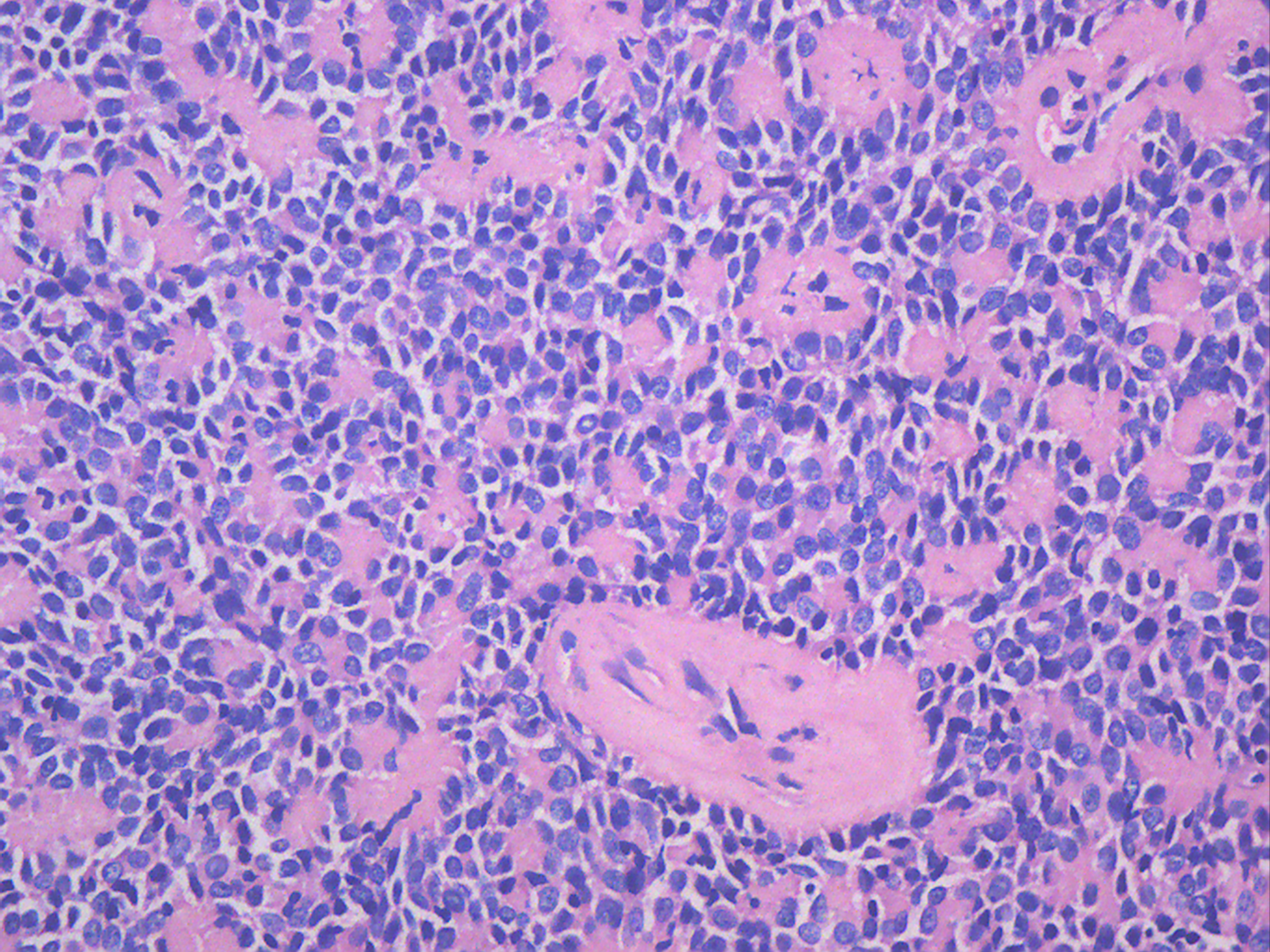

Supplement: S5 Fig — (ZIP) [file pone.0273682.s005.zip › 03.tif]

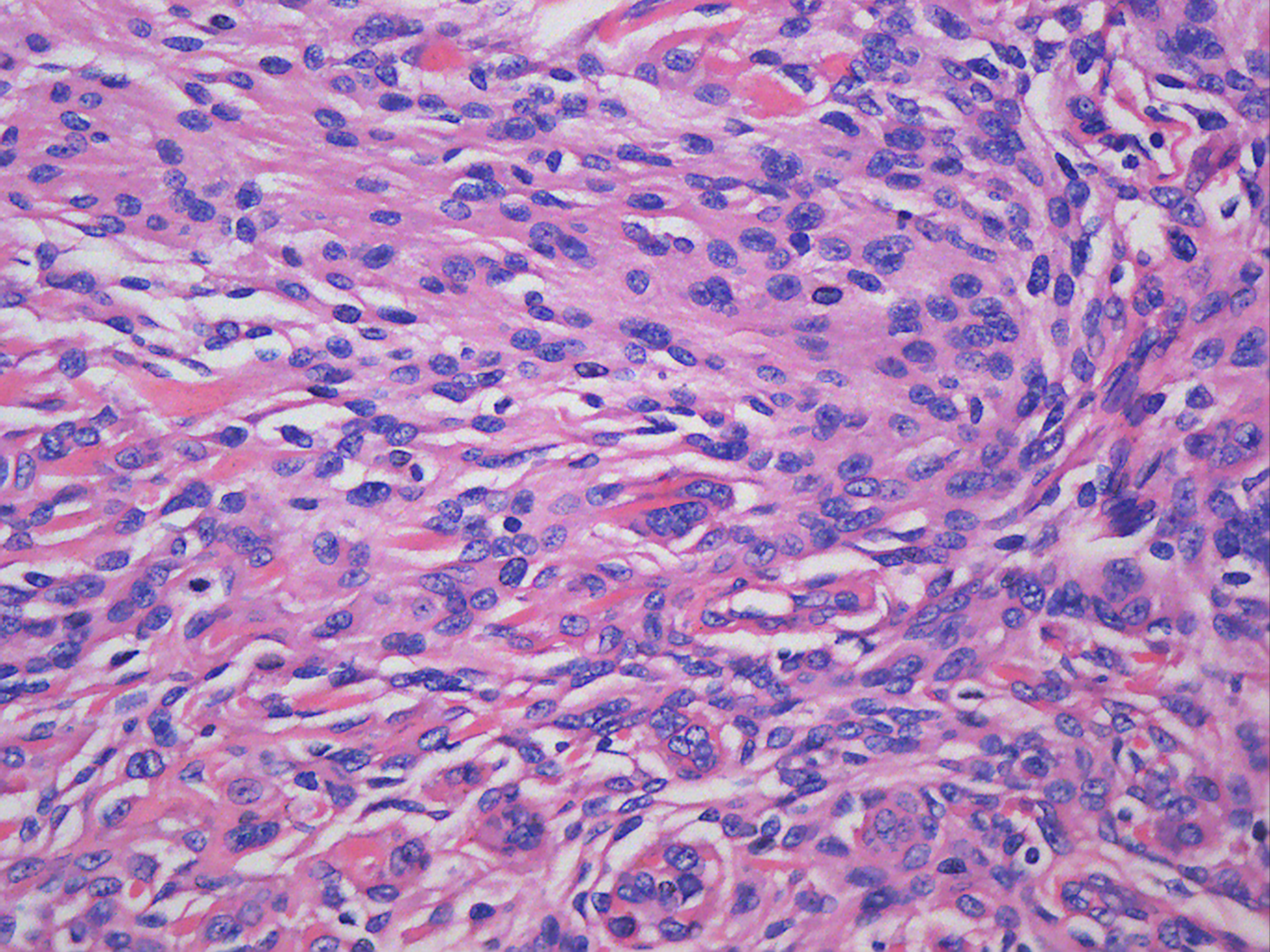

Supplement: S5 Fig — (ZIP) [file pone.0273682.s005.zip › 04.tif]

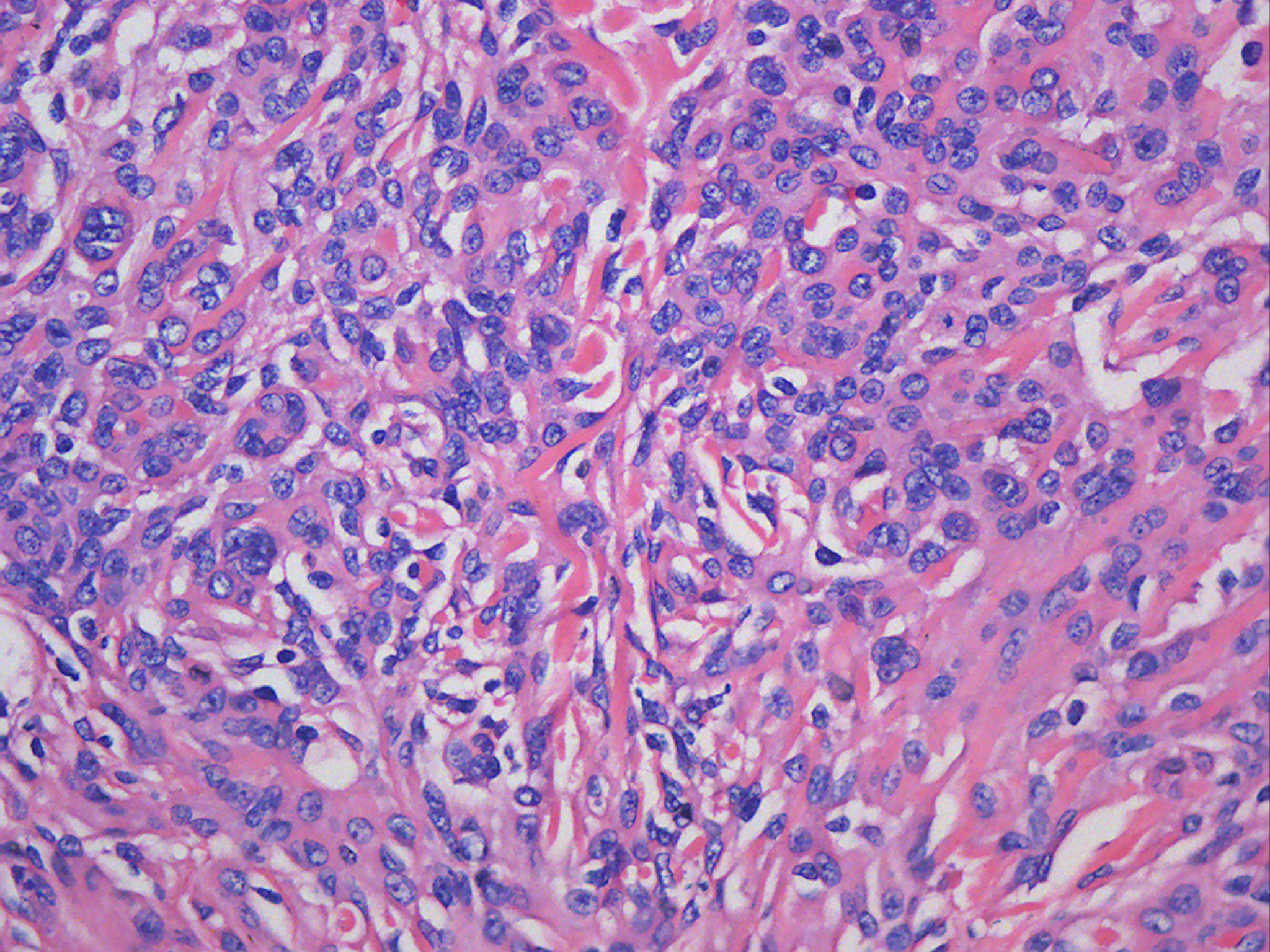

Supplement: S5 Fig — (ZIP) [file pone.0273682.s005.zip › 05.tif]

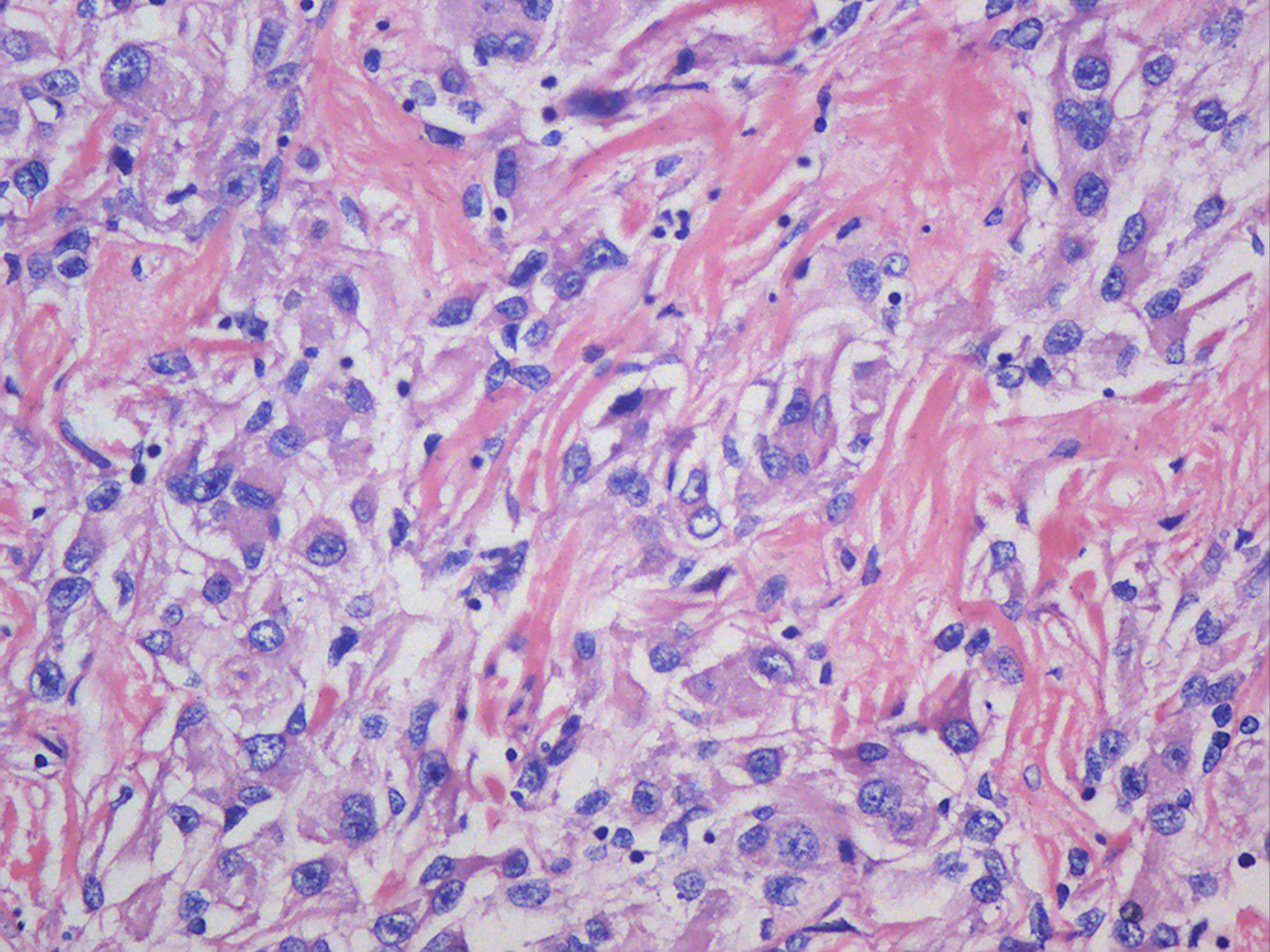

Supplement: S5 Fig — (ZIP) [file pone.0273682.s005.zip › 06.tif]

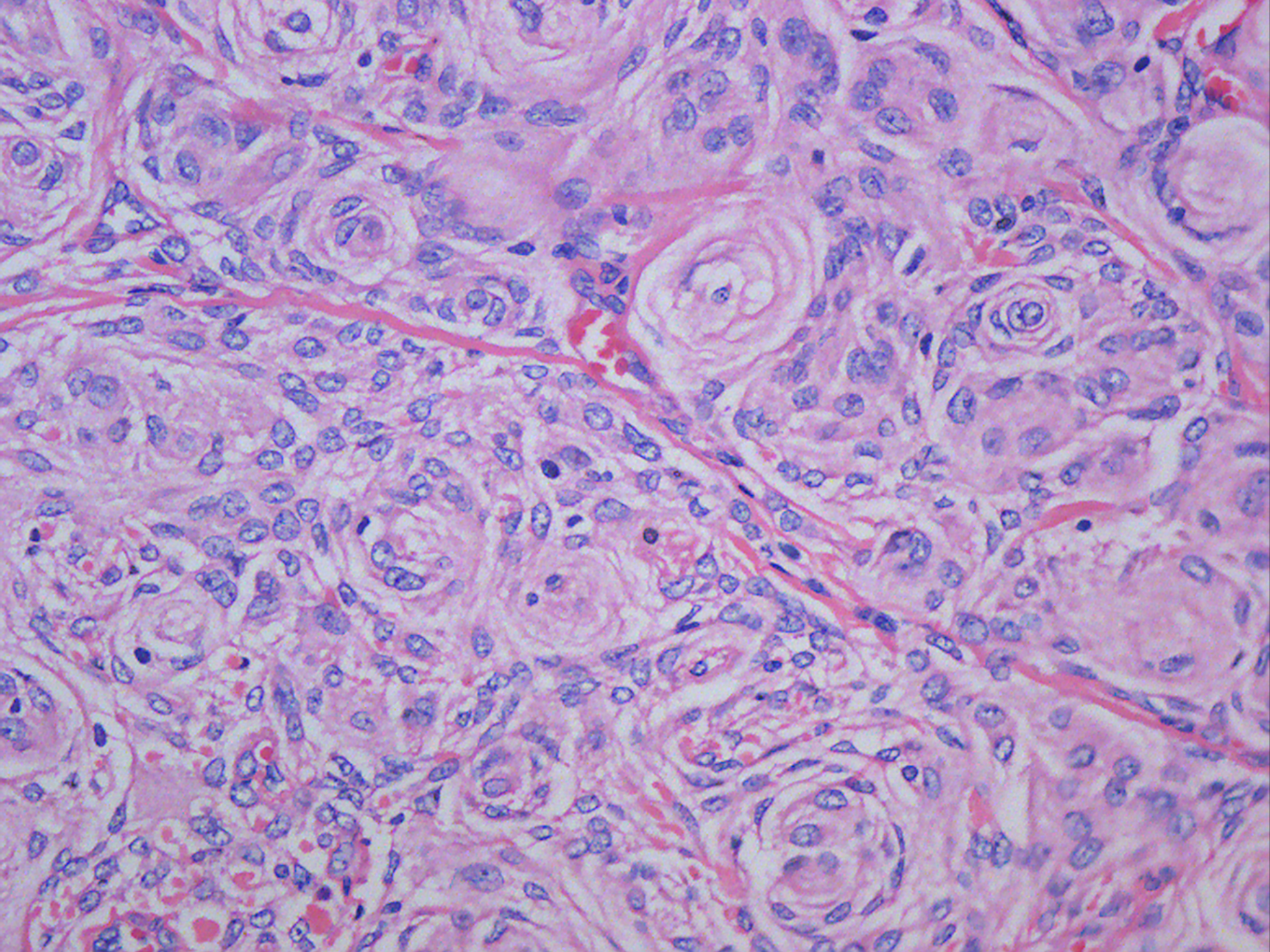

Supplement: S5 Fig — (ZIP) [file pone.0273682.s005.zip › 07.tif]

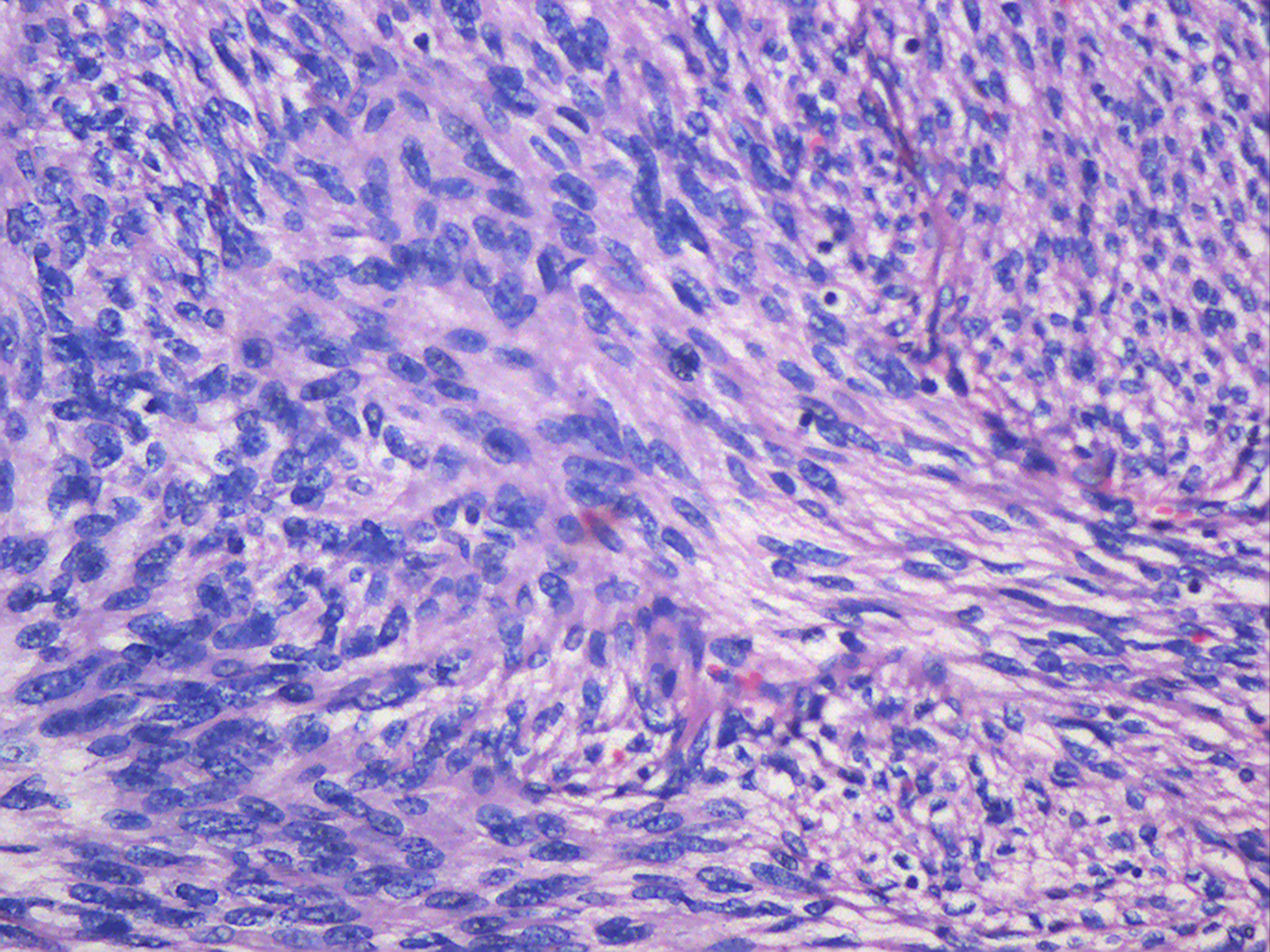

Supplement: S5 Fig — (ZIP) [file pone.0273682.s005.zip › 08.tif]

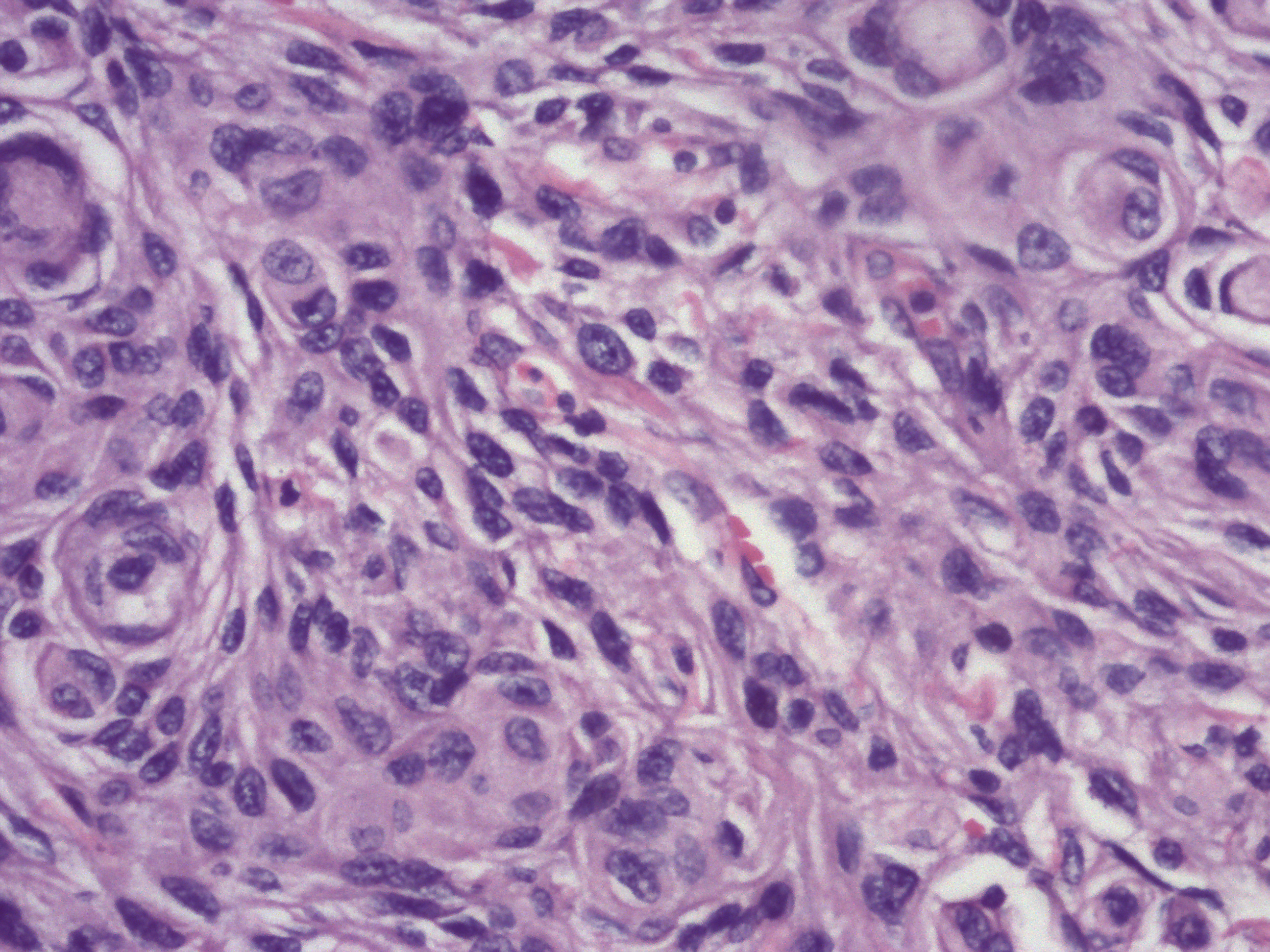

Supplement: S5 Fig — (ZIP) [file pone.0273682.s005.zip › 09.tif]

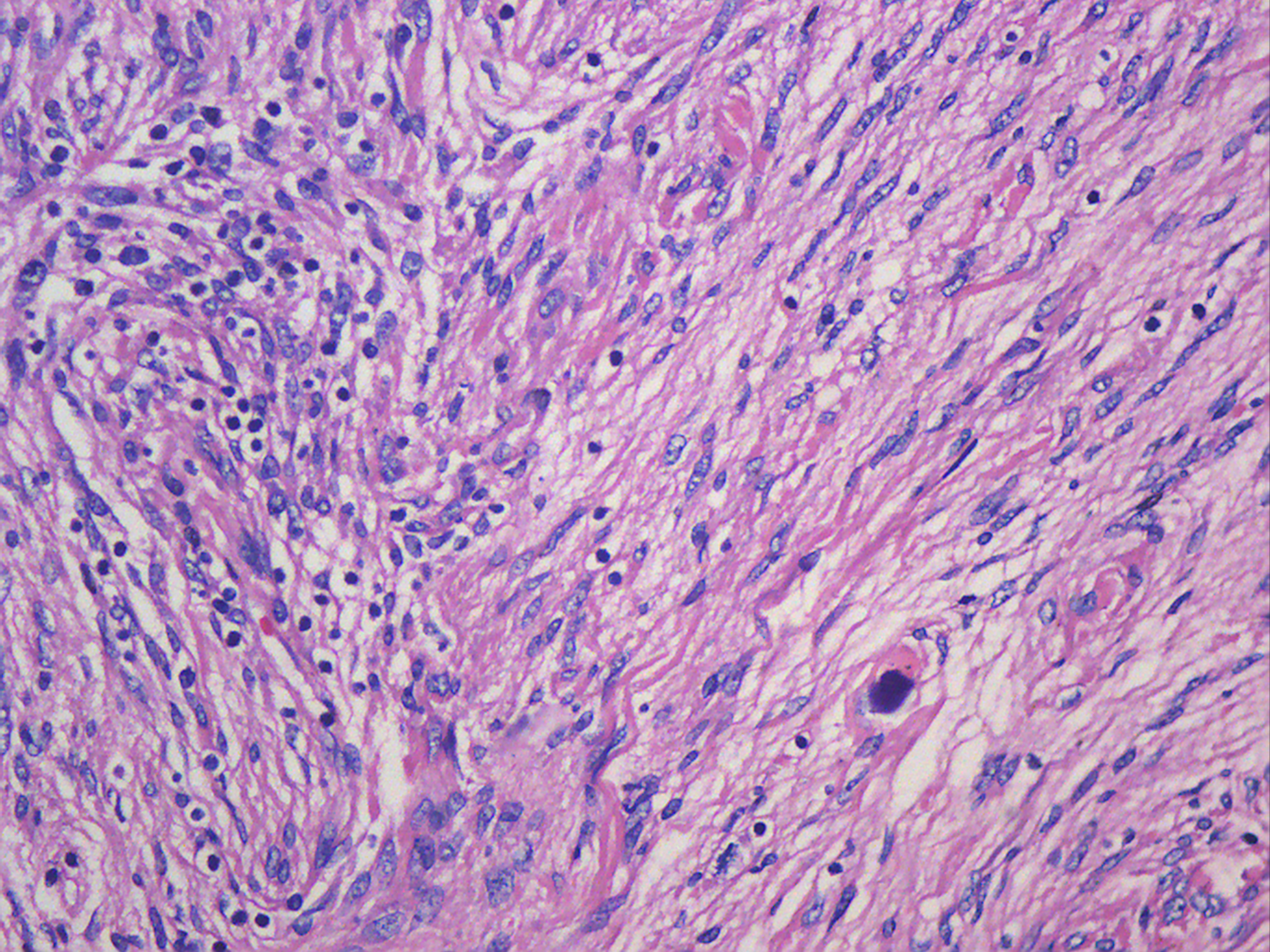

Supplement: S5 Fig — (ZIP) [file pone.0273682.s005.zip › 10.tif]

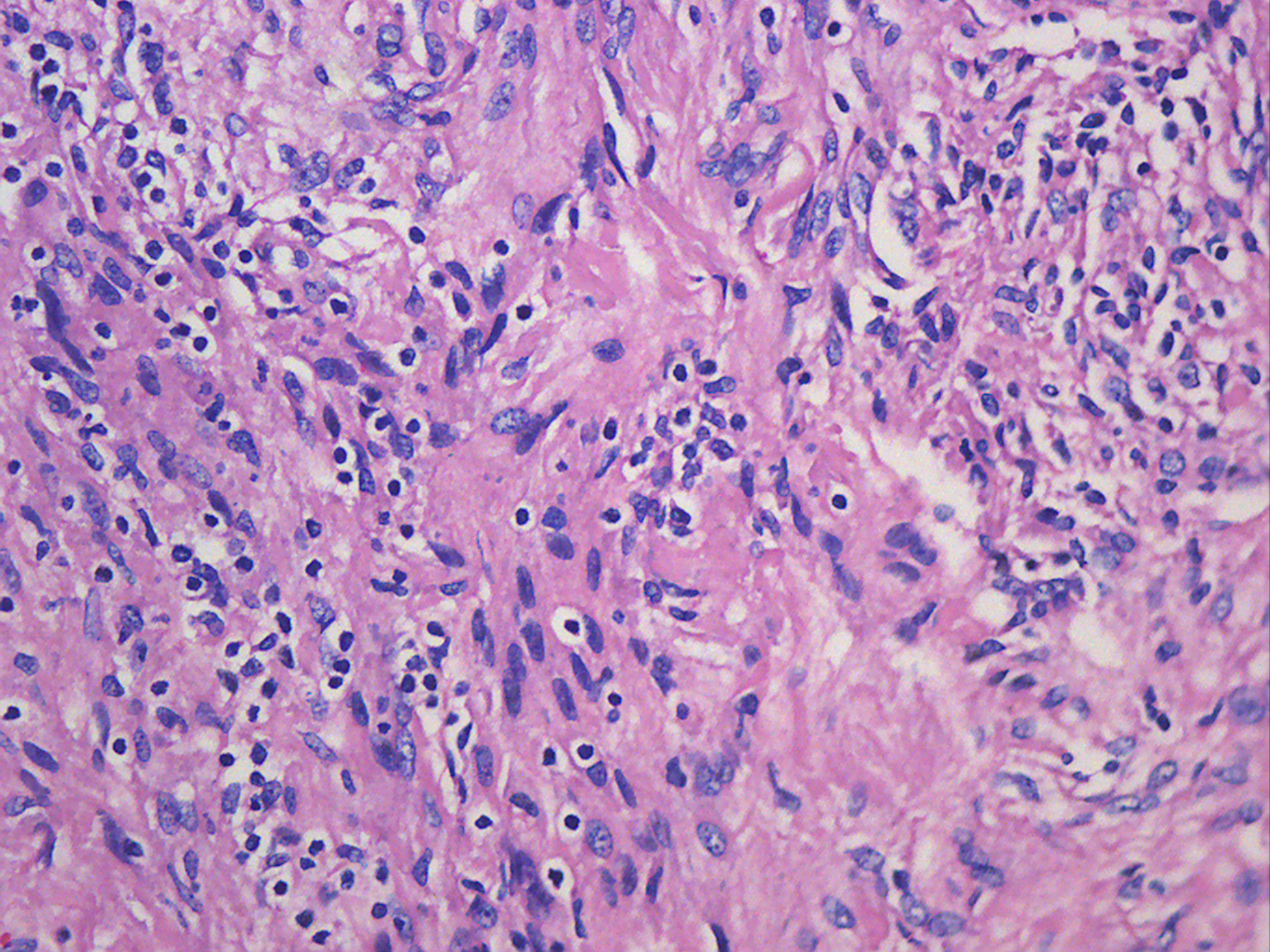

Supplement: S5 Fig — (ZIP) [file pone.0273682.s005.zip › 11.tif]

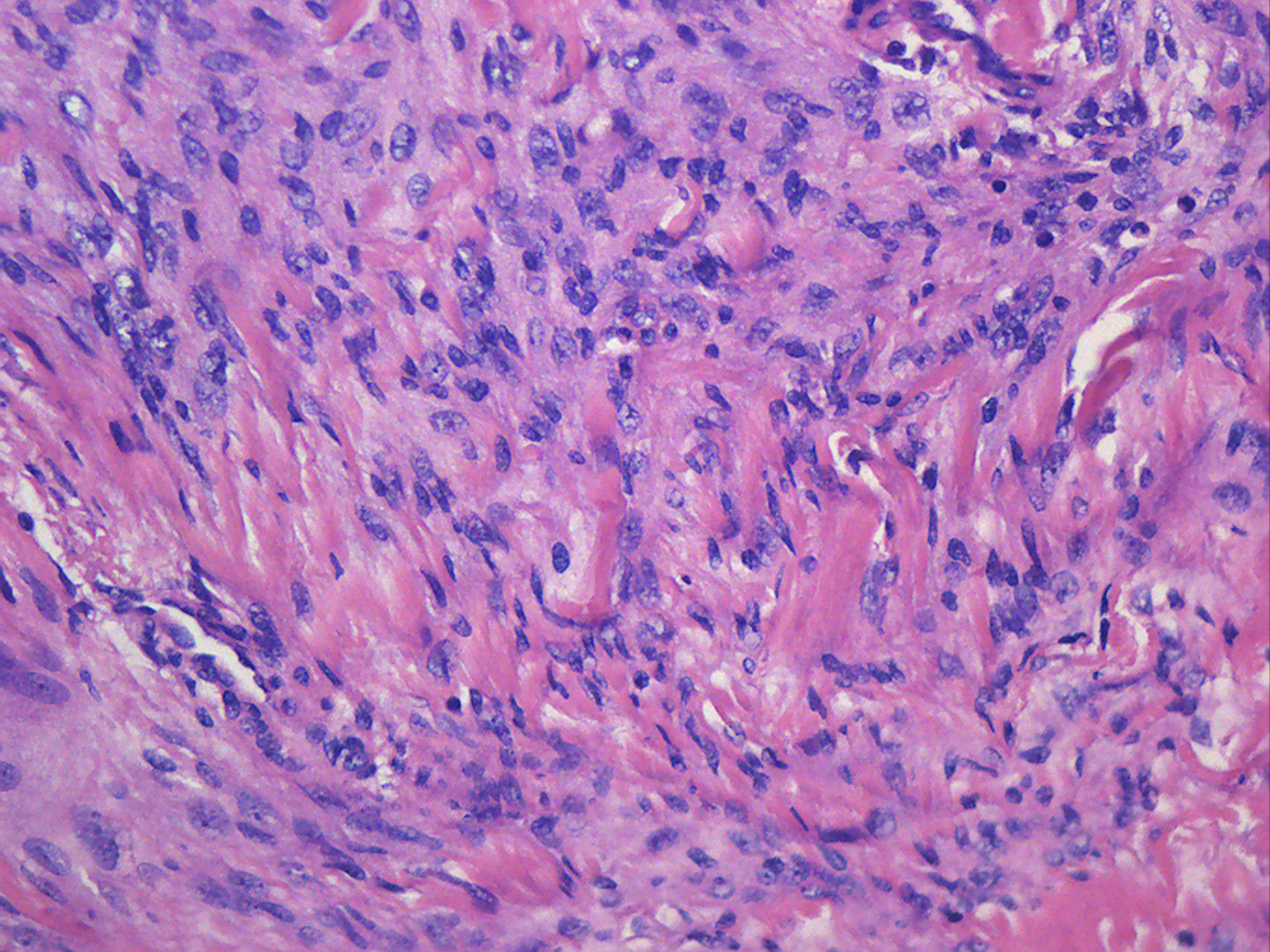

Supplement: S5 Fig — (ZIP) [file pone.0273682.s005.zip › 12.tif]

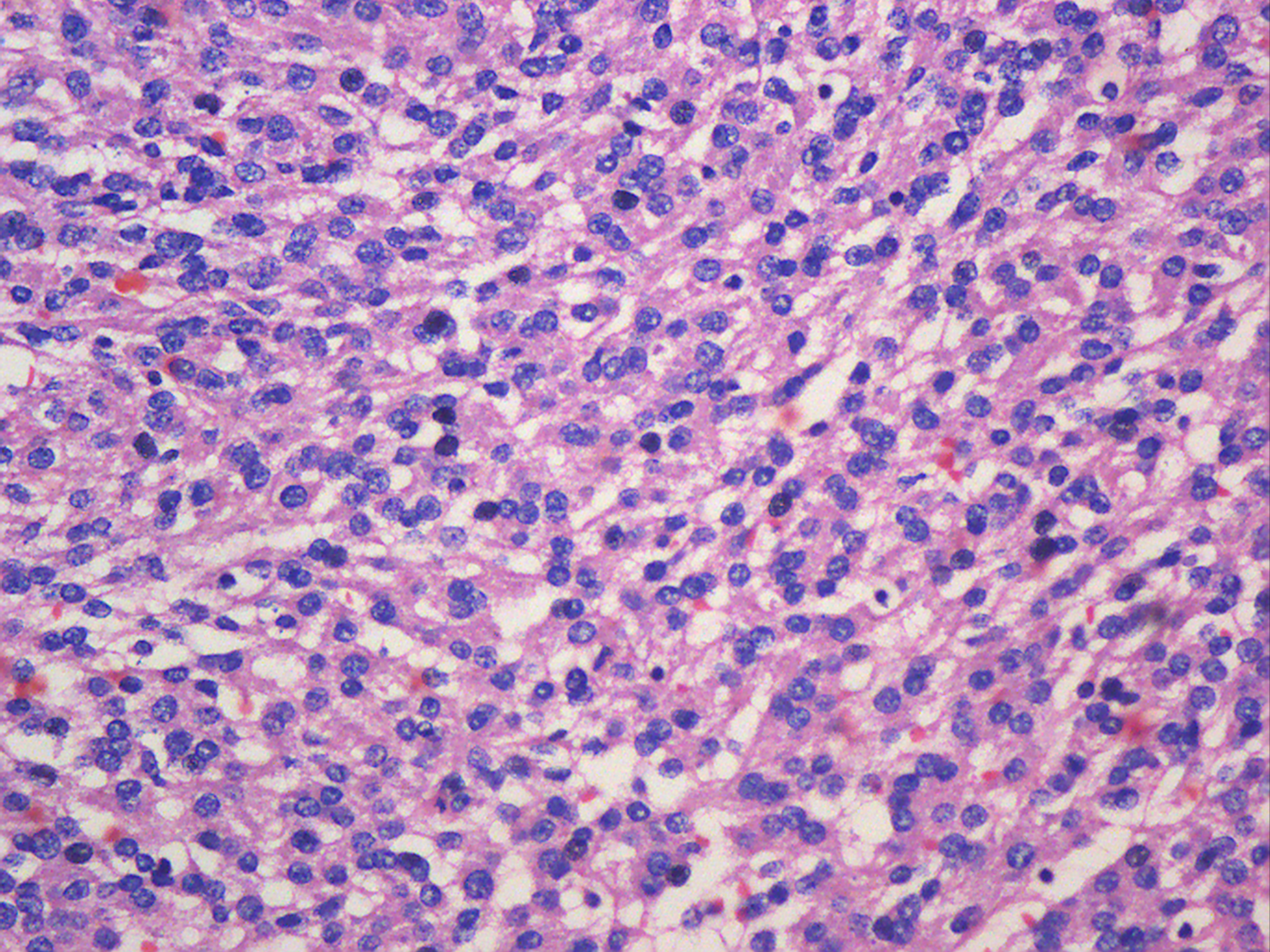

Supplement: S6 Fig — (ZIP) [file pone.0273682.s006.zip › 13.tif]

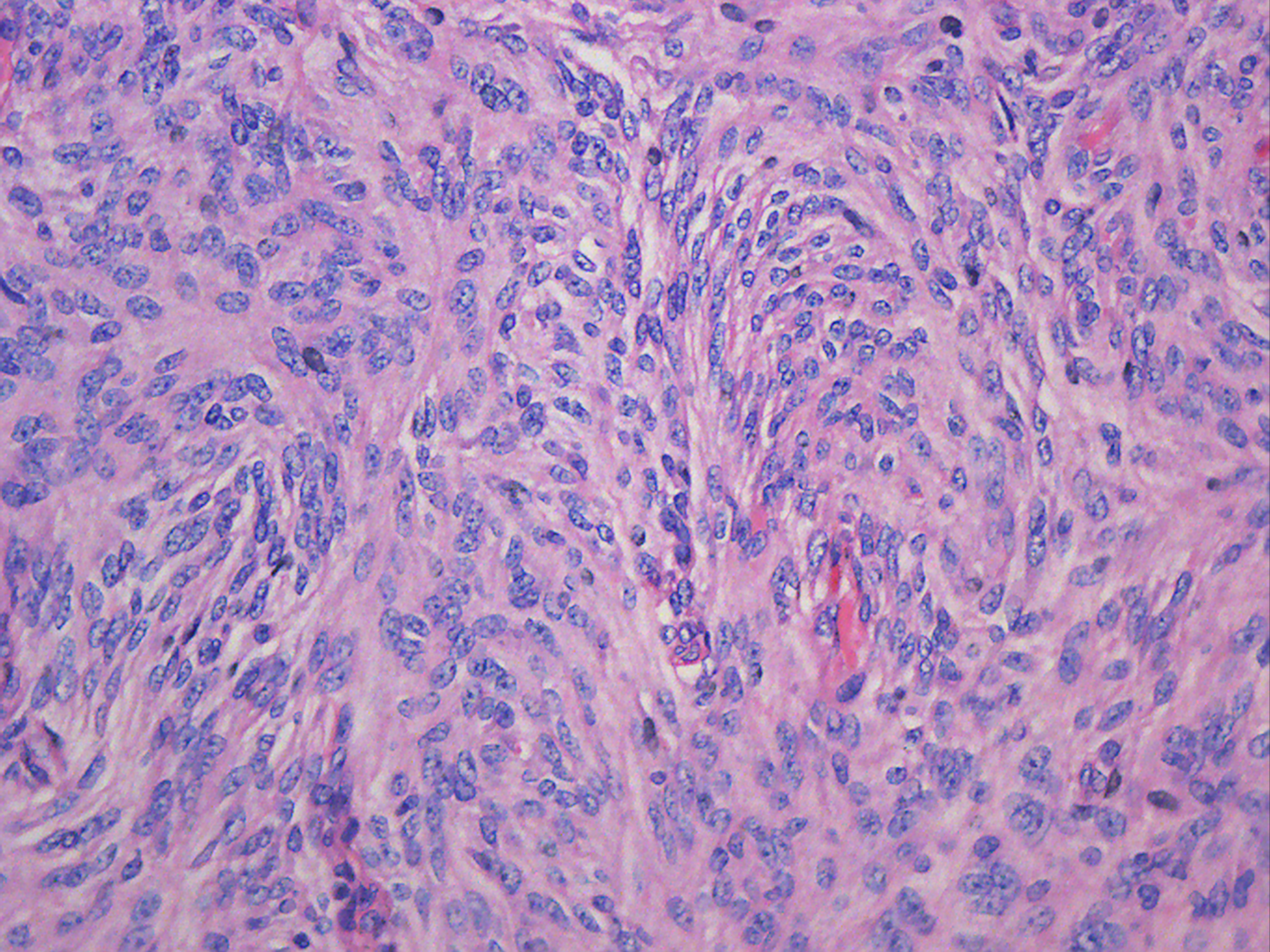

Supplement: S6 Fig — (ZIP) [file pone.0273682.s006.zip › 14.tif]

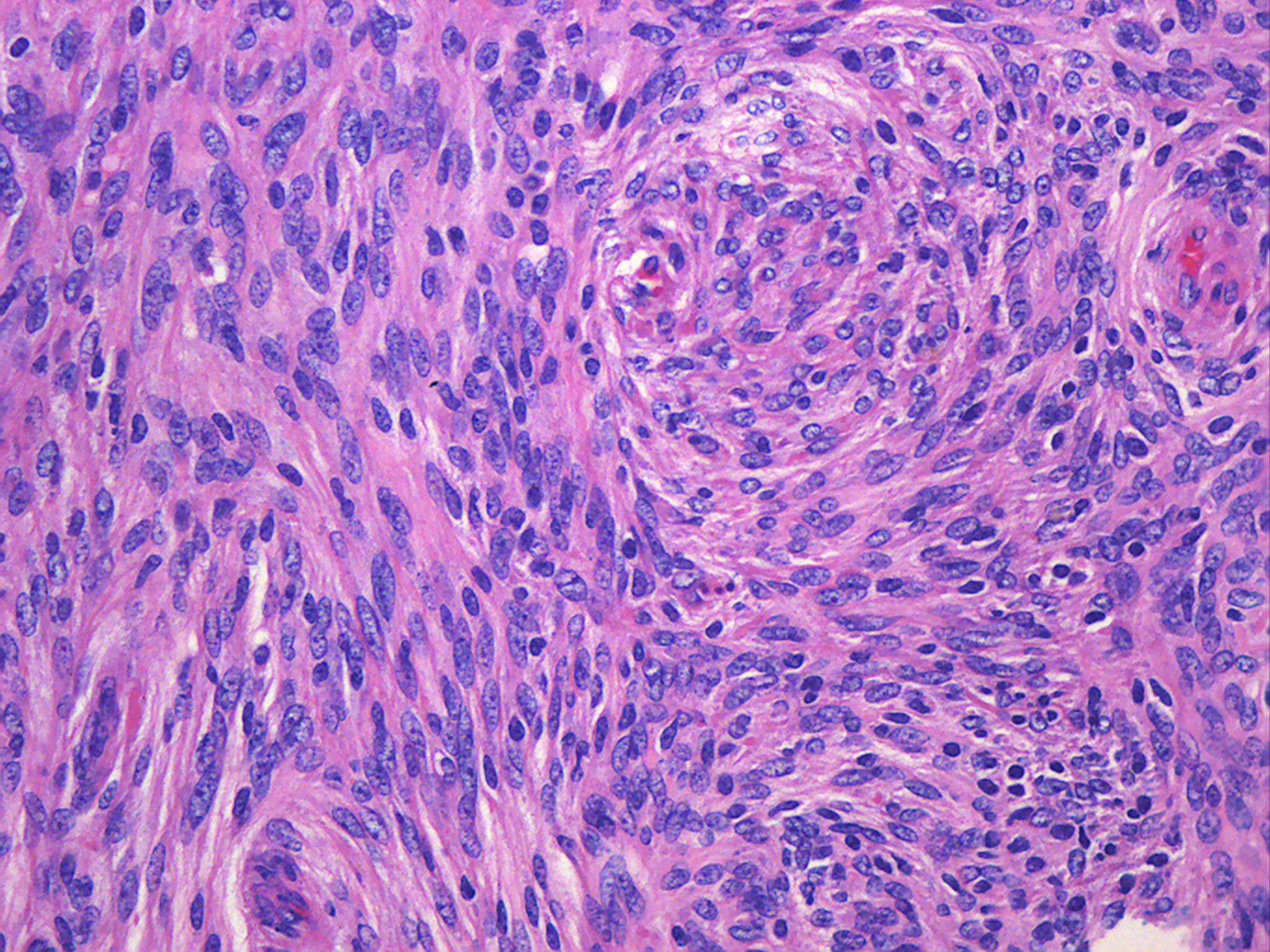

Supplement: S6 Fig — (ZIP) [file pone.0273682.s006.zip › 15.tif]

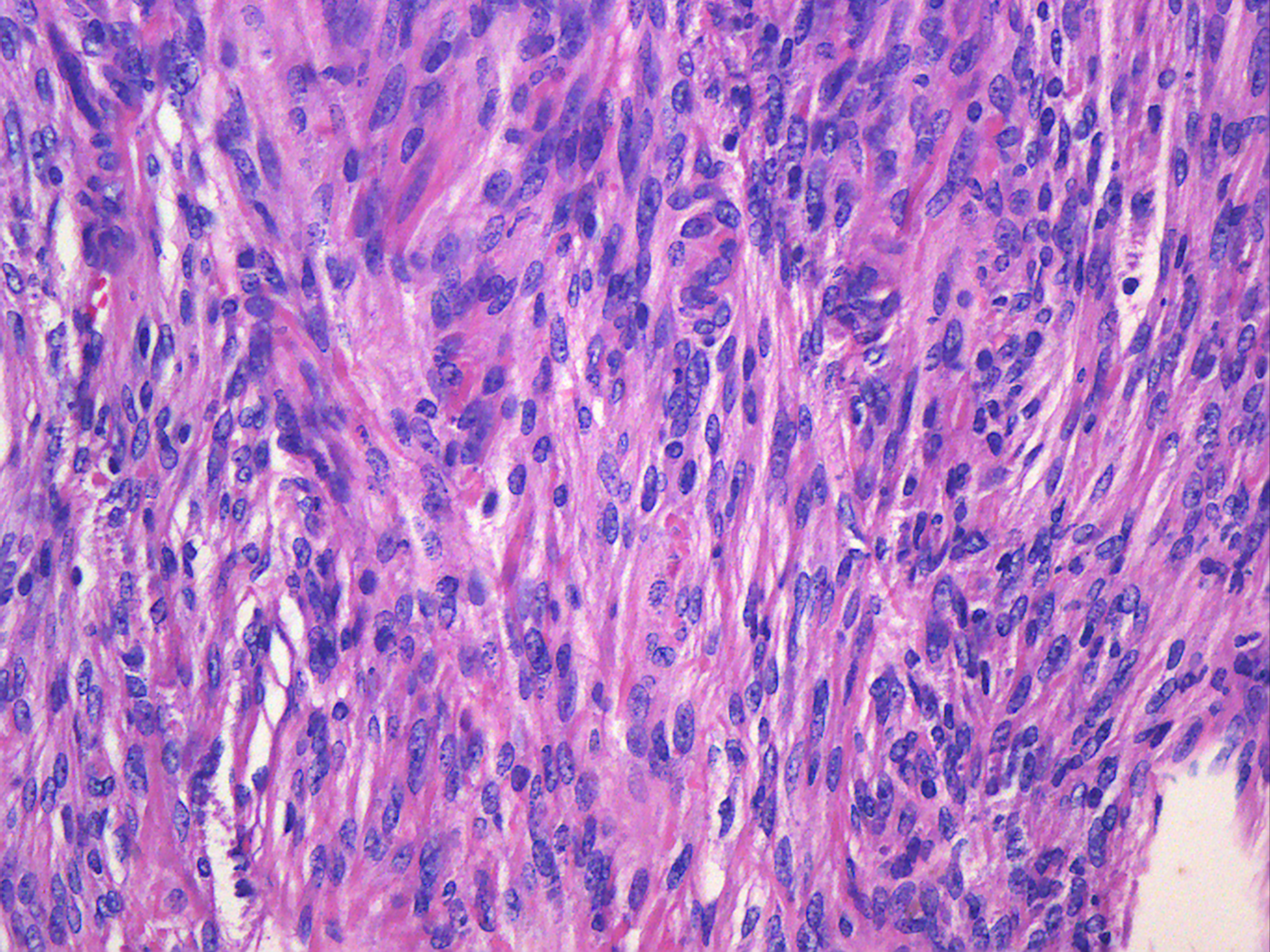

Supplement: S6 Fig — (ZIP) [file pone.0273682.s006.zip › 16.tif]

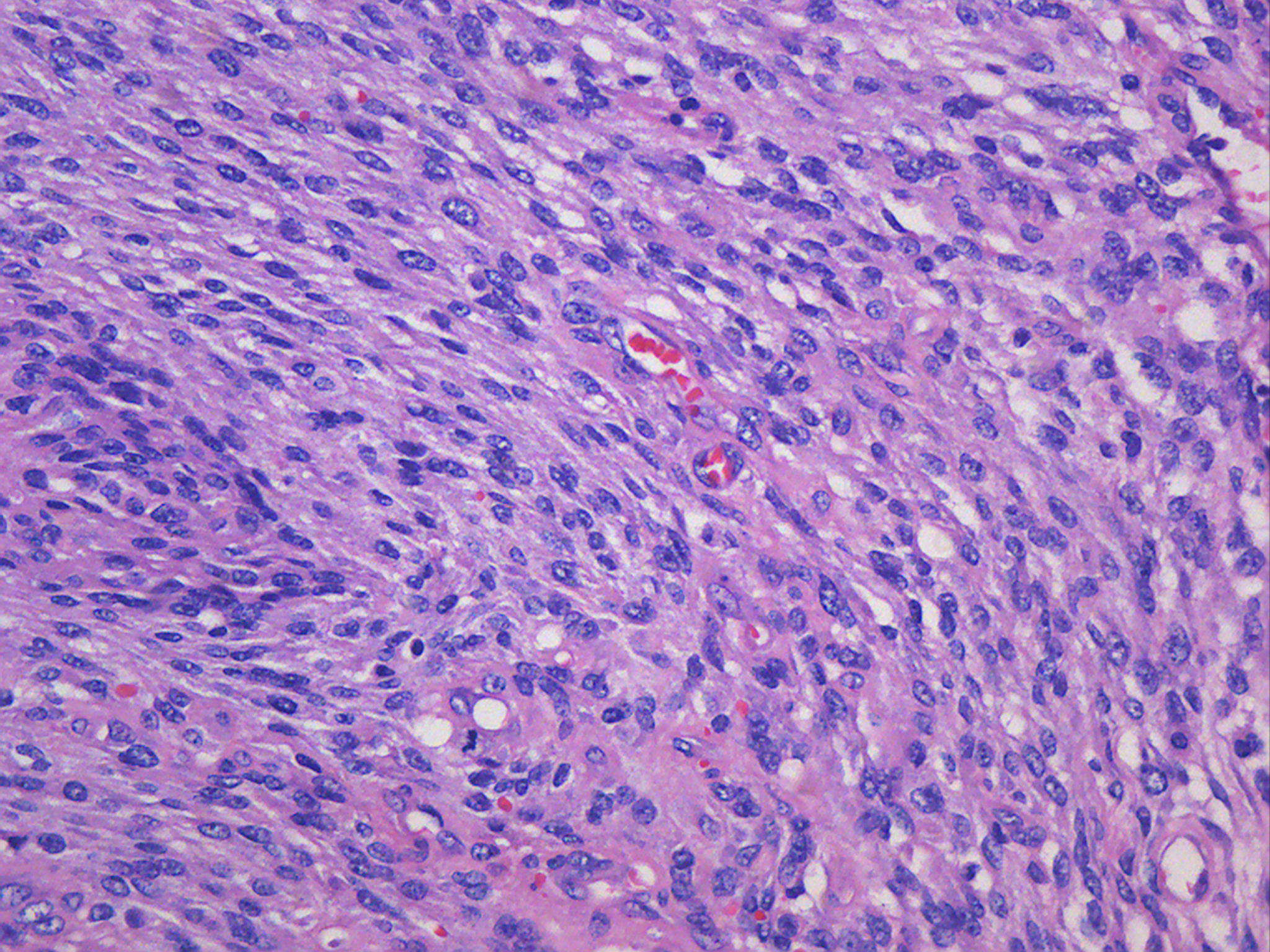

Supplement: S6 Fig — (ZIP) [file pone.0273682.s006.zip › 17.tif]

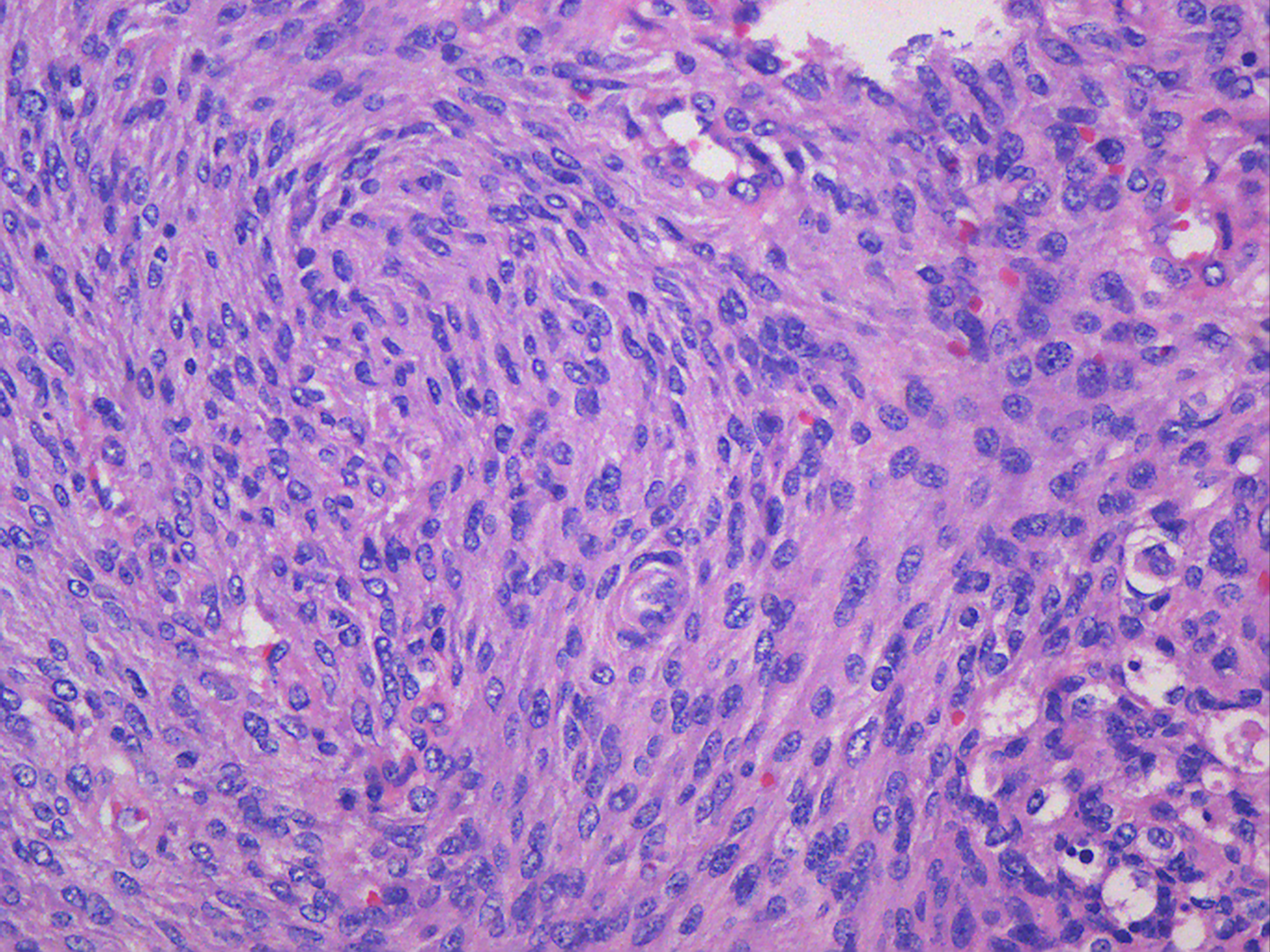

Supplement: S6 Fig — (ZIP) [file pone.0273682.s006.zip › 18.tif]

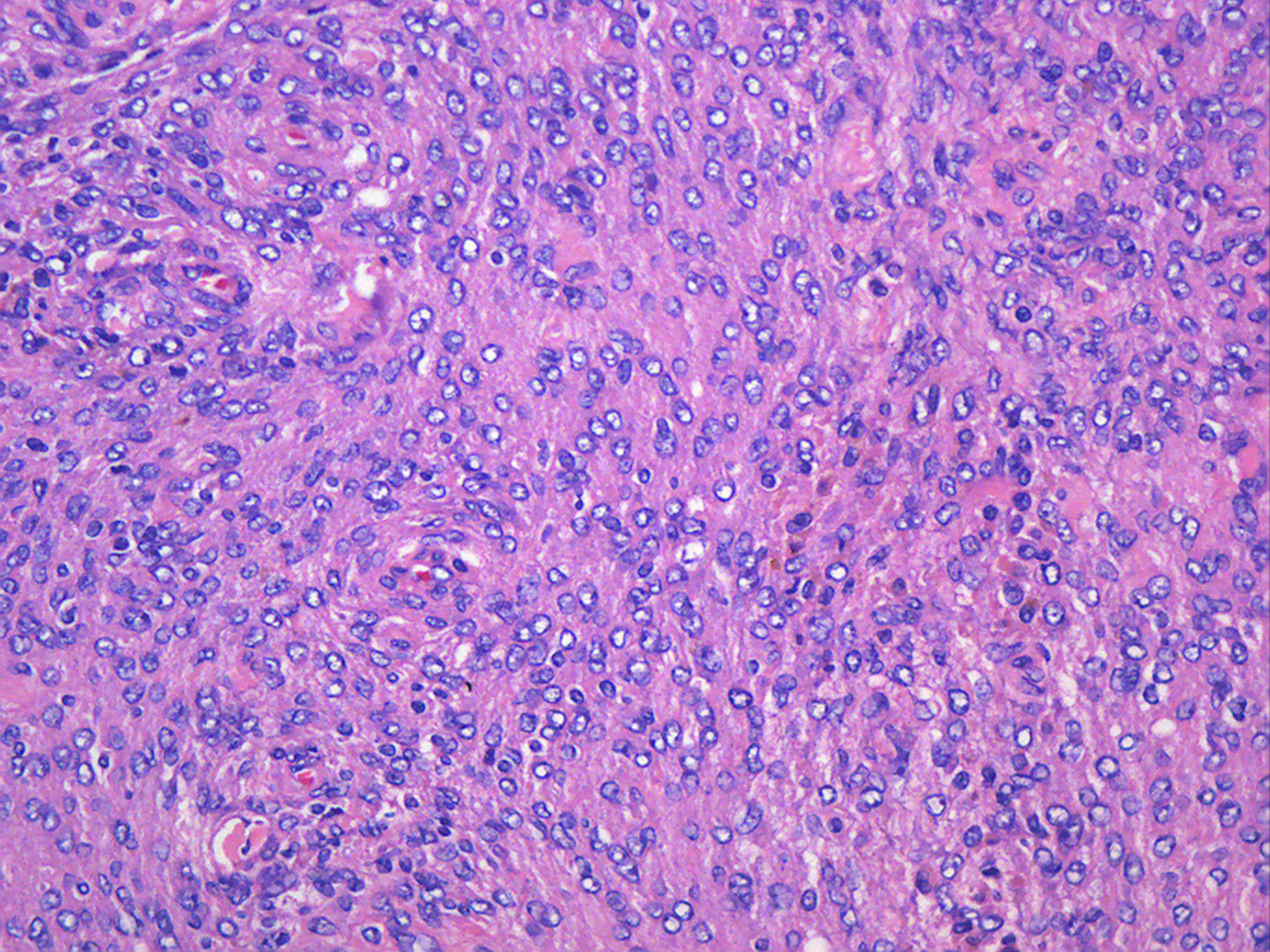

Supplement: S6 Fig — (ZIP) [file pone.0273682.s006.zip › 19.tif]

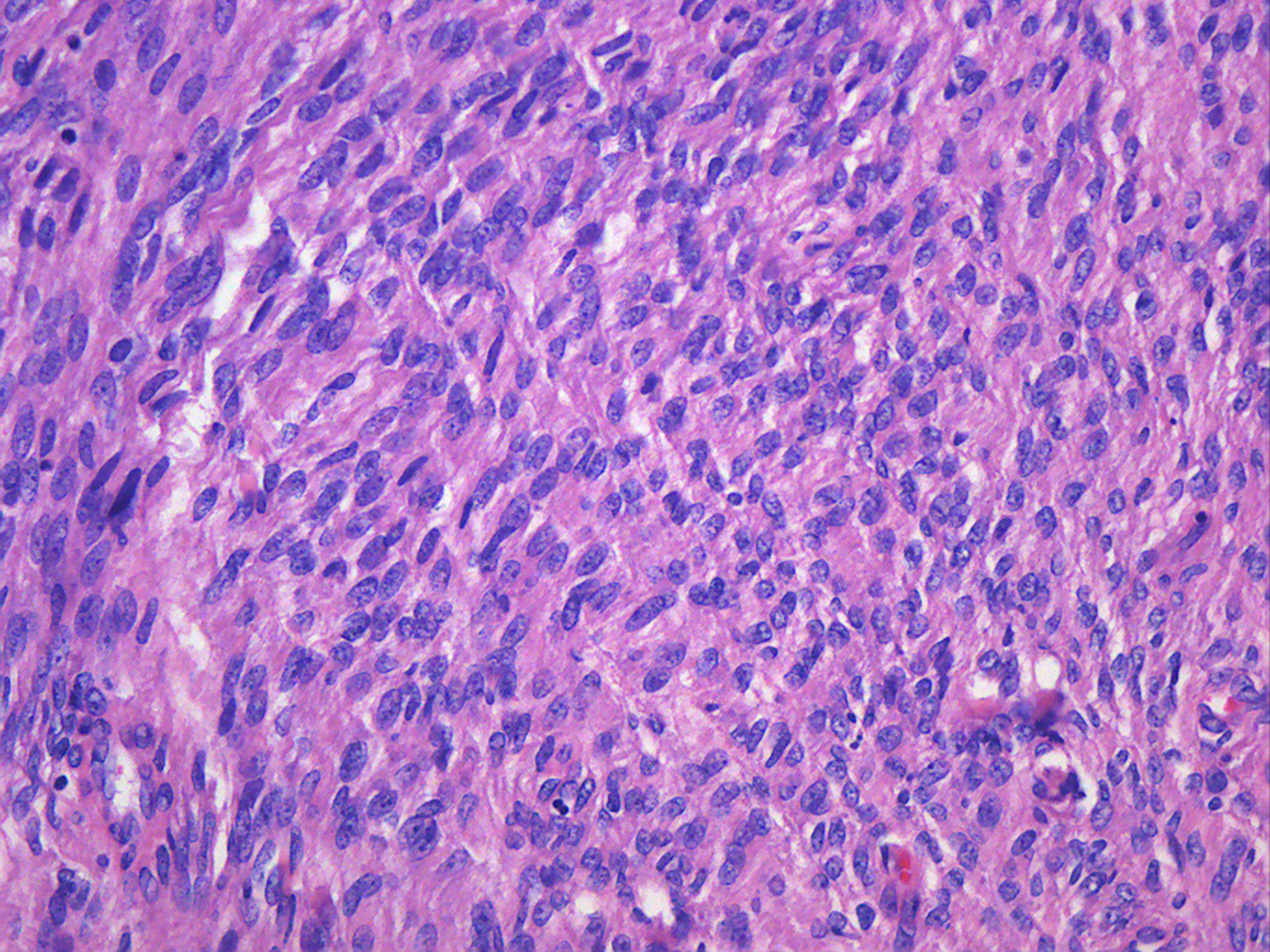

Supplement: S6 Fig — (ZIP) [file pone.0273682.s006.zip › 20.tif]

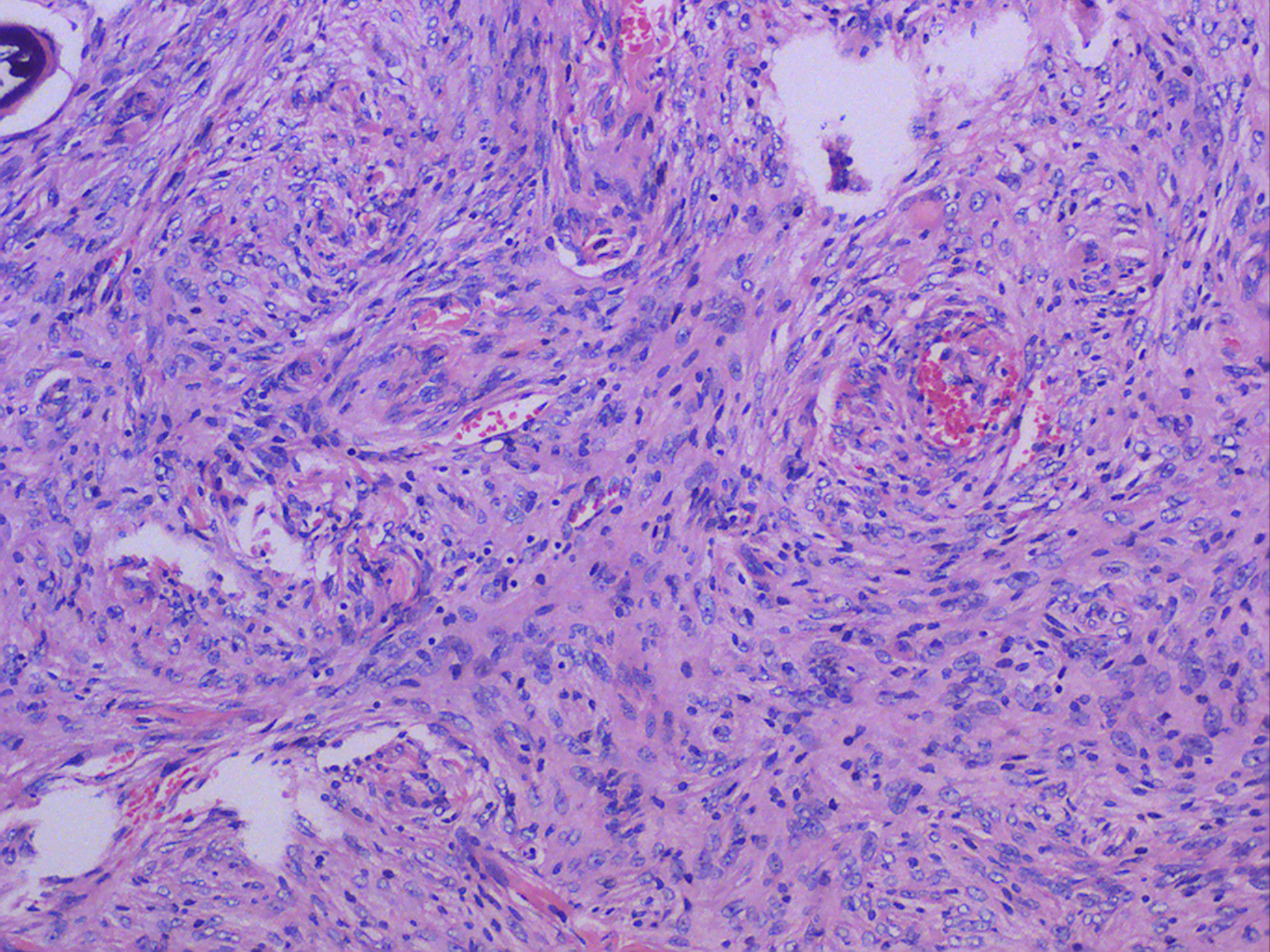

Supplement: S6 Fig — (ZIP) [file pone.0273682.s006.zip › 21.tif]

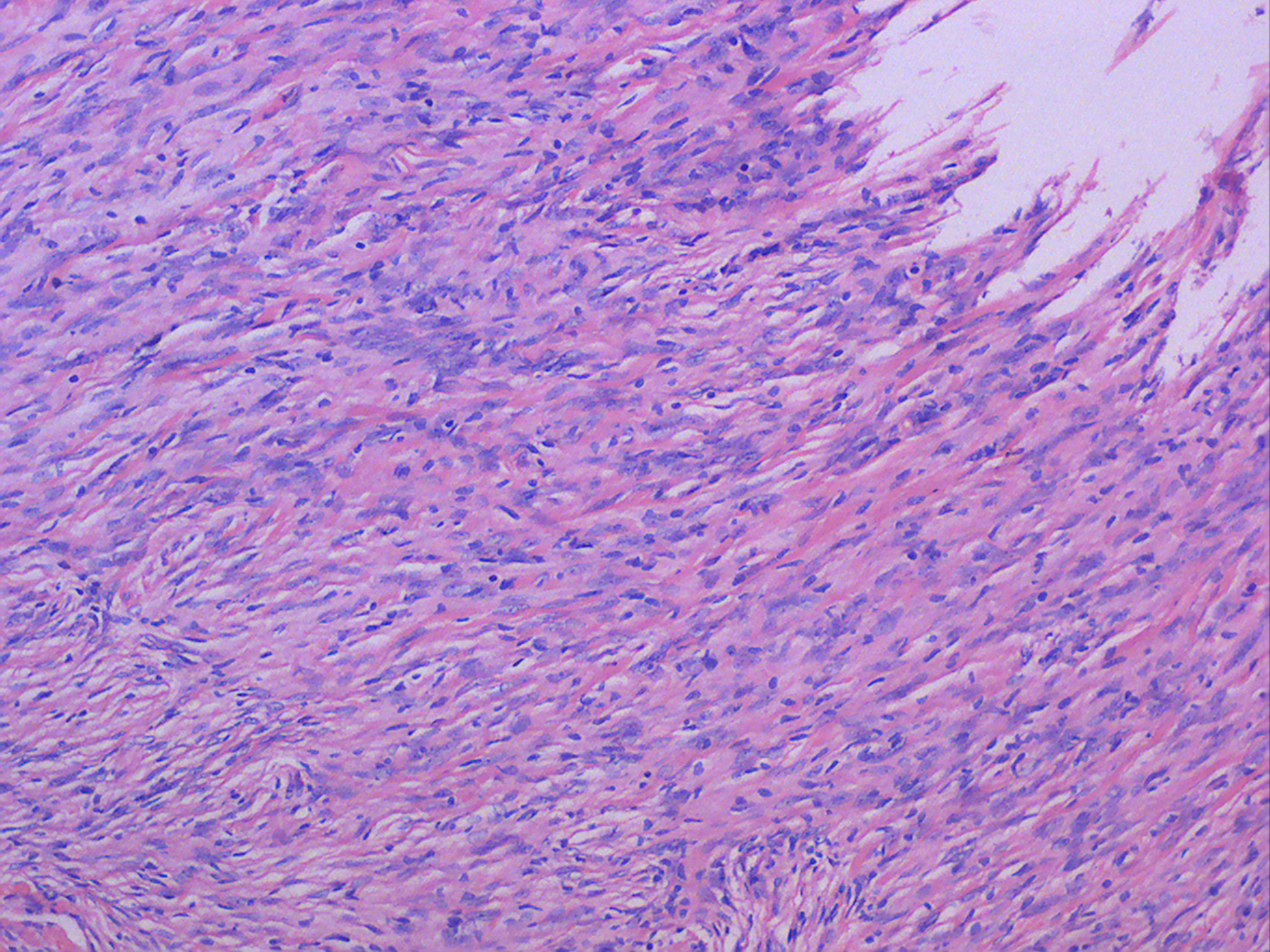

Supplement: S6 Fig — (ZIP) [file pone.0273682.s006.zip › 22.tif]

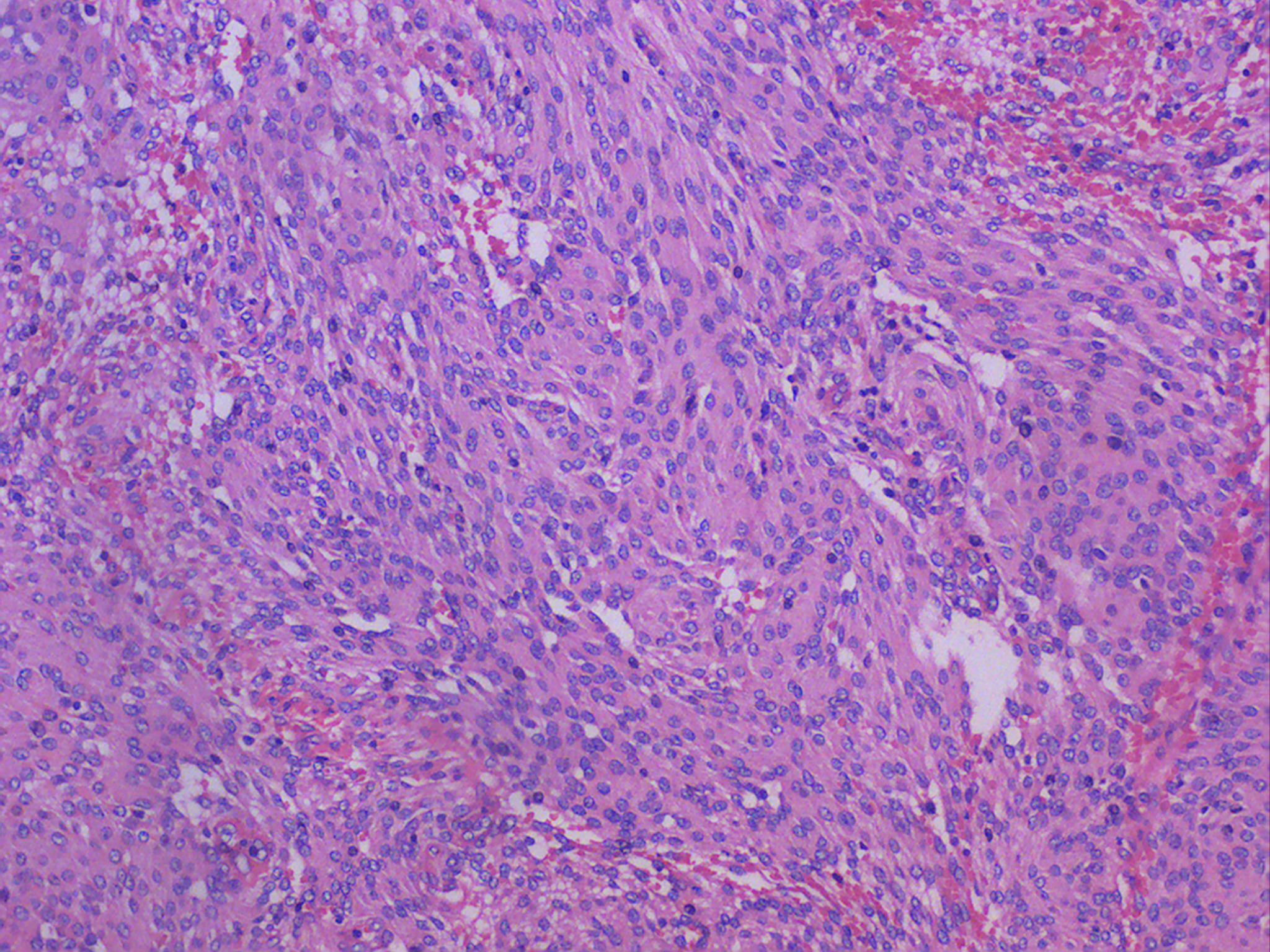

Supplement: S6 Fig — (ZIP) [file pone.0273682.s006.zip › 23.tif]

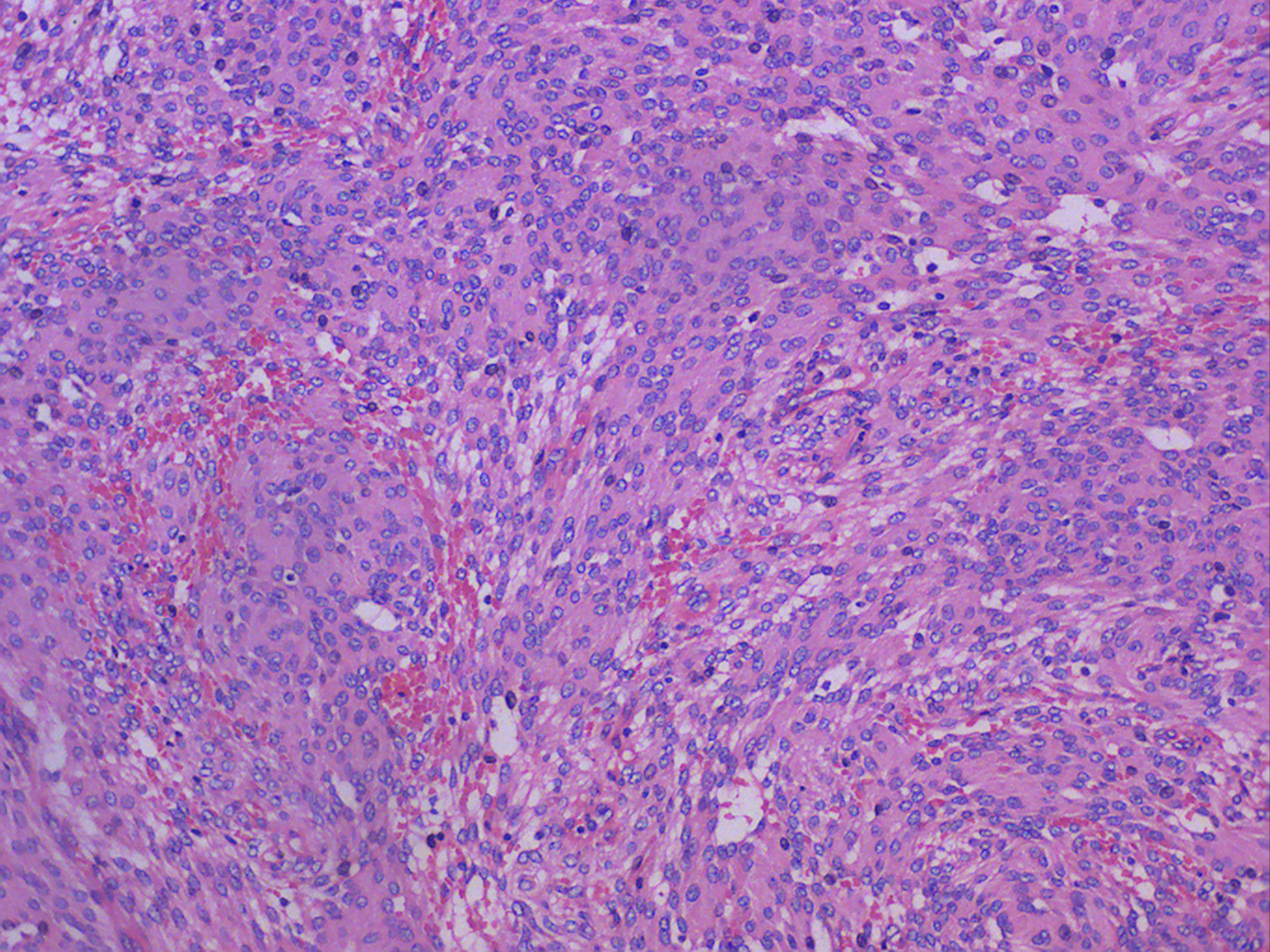

Supplement: S6 Fig — (ZIP) [file pone.0273682.s006.zip › 24.tif]

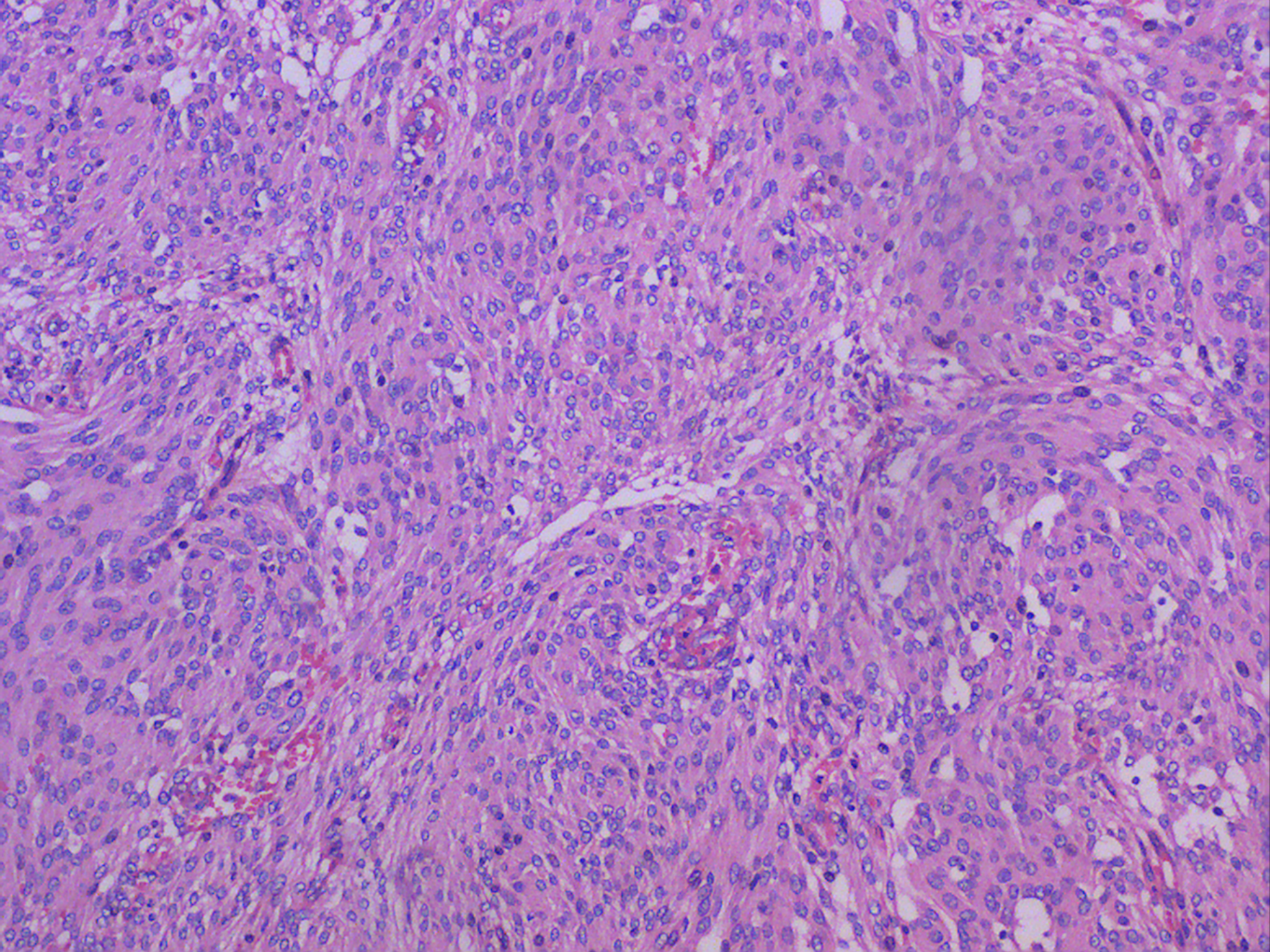

Supplement: S7 Fig — (ZIP) [file pone.0273682.s007.zip › 25.tif]

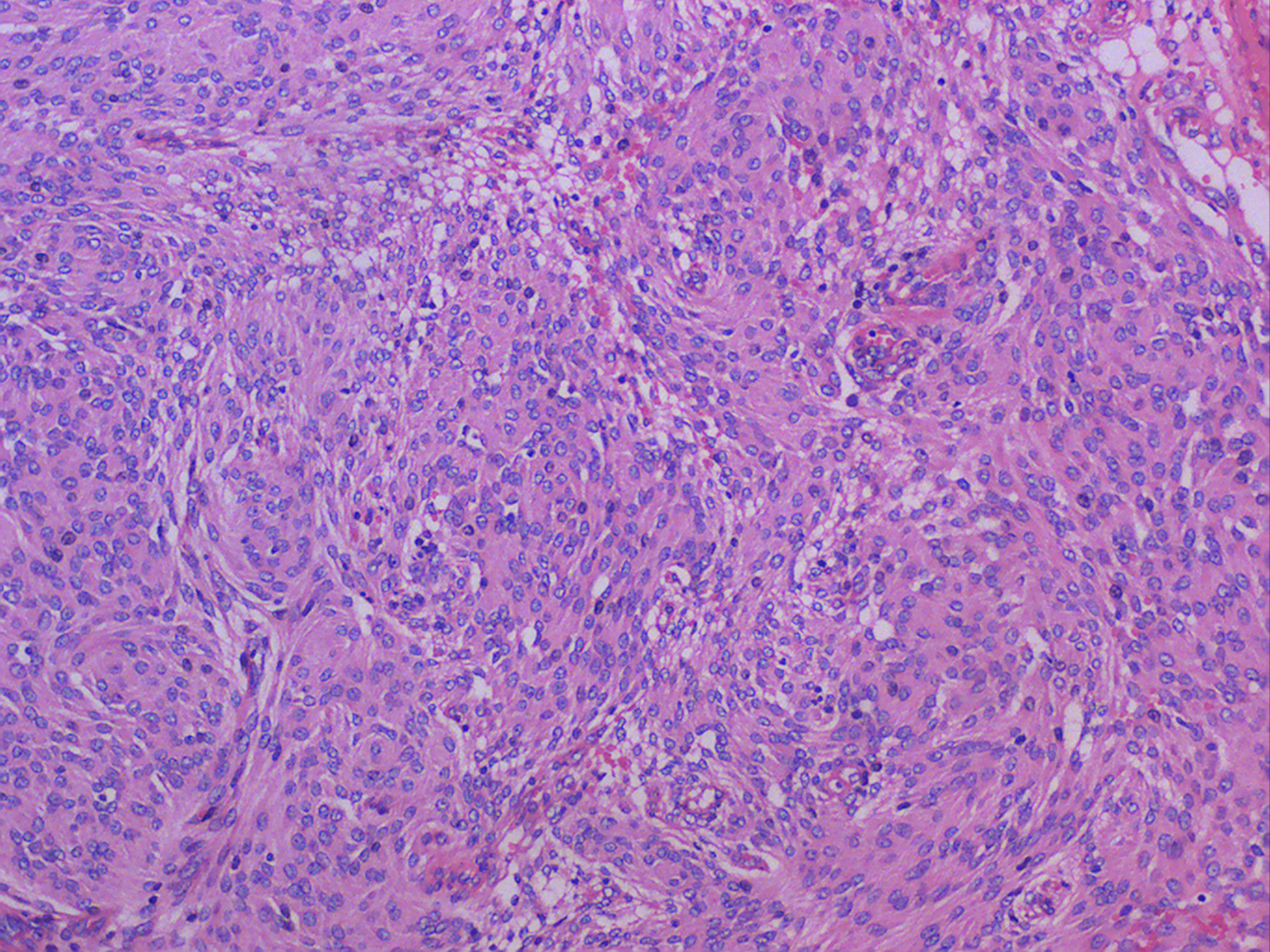

Supplement: S7 Fig — (ZIP) [file pone.0273682.s007.zip › 26.tif]

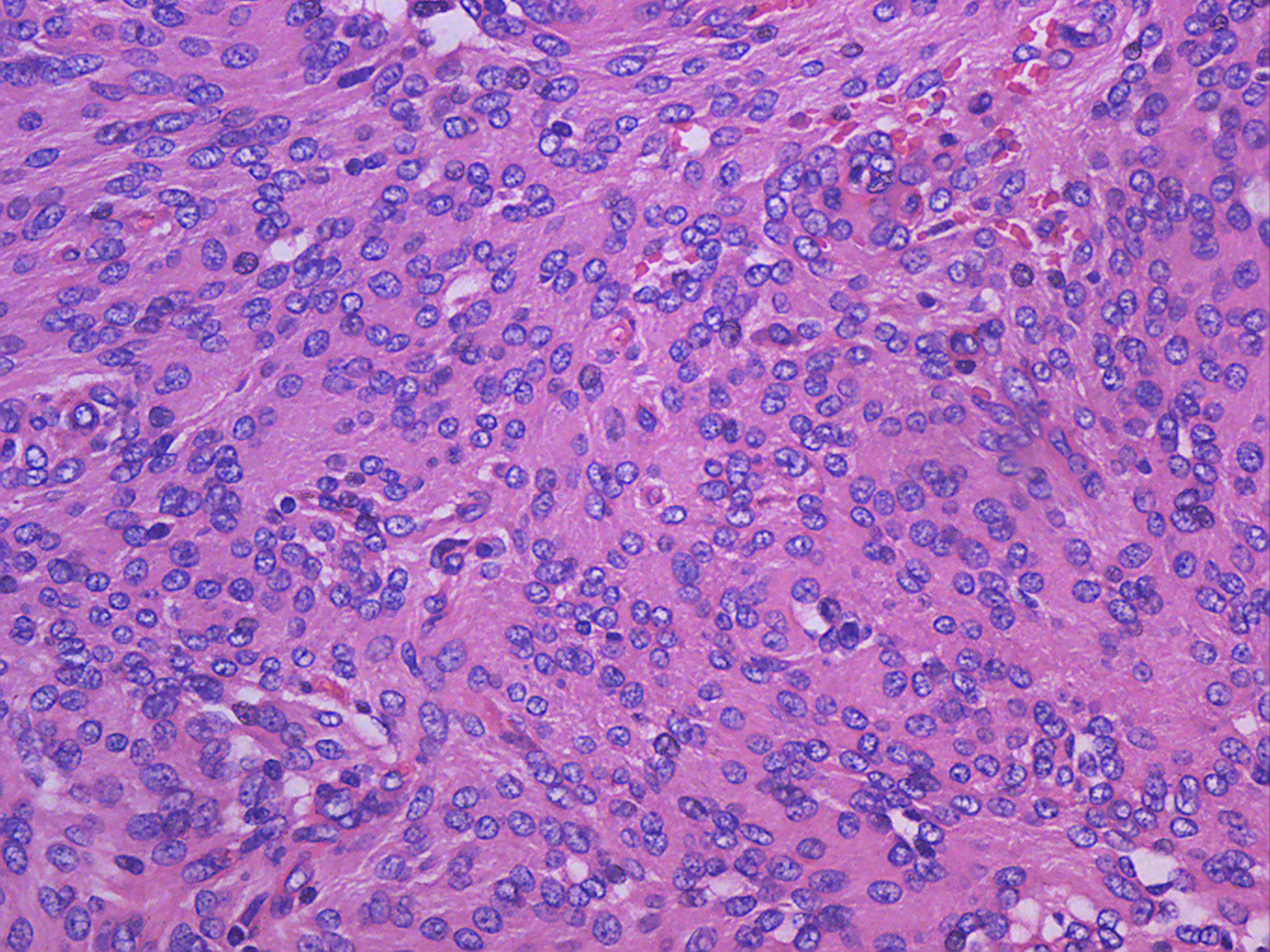

Supplement: S7 Fig — (ZIP) [file pone.0273682.s007.zip › 27.tif]

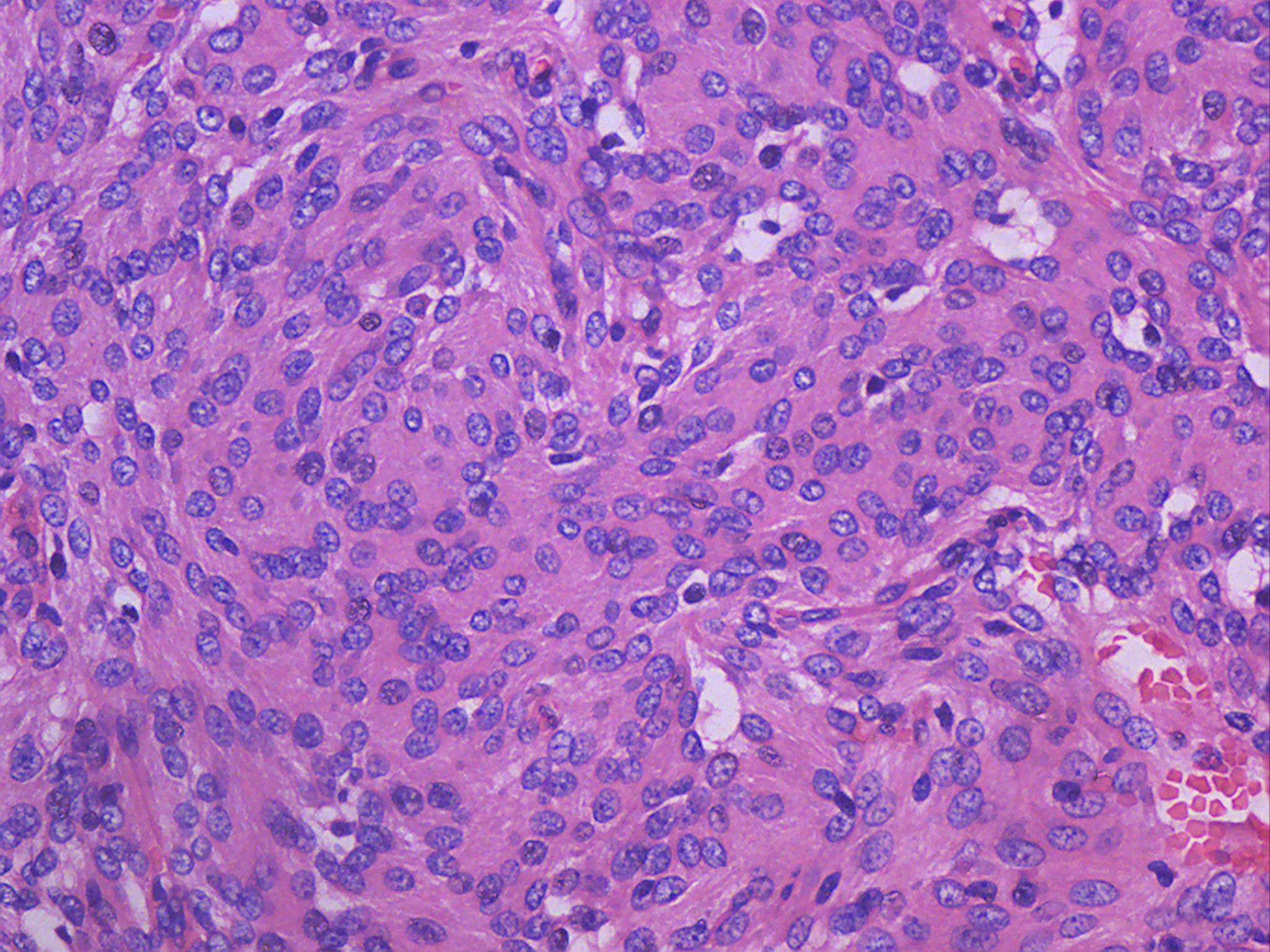

Supplement: S7 Fig — (ZIP) [file pone.0273682.s007.zip › 28.tif]

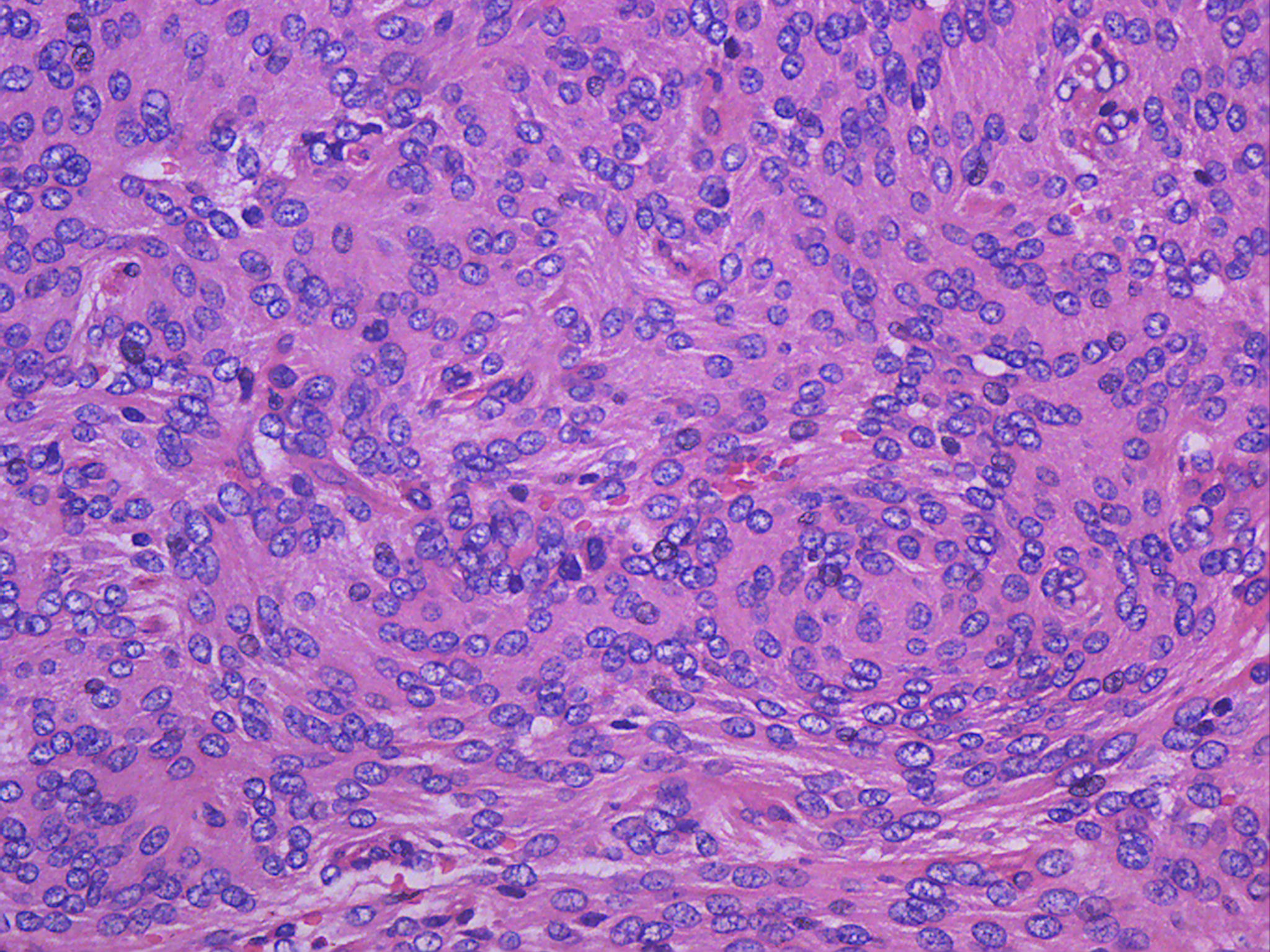

Supplement: S7 Fig — (ZIP) [file pone.0273682.s007.zip › 29.tif]

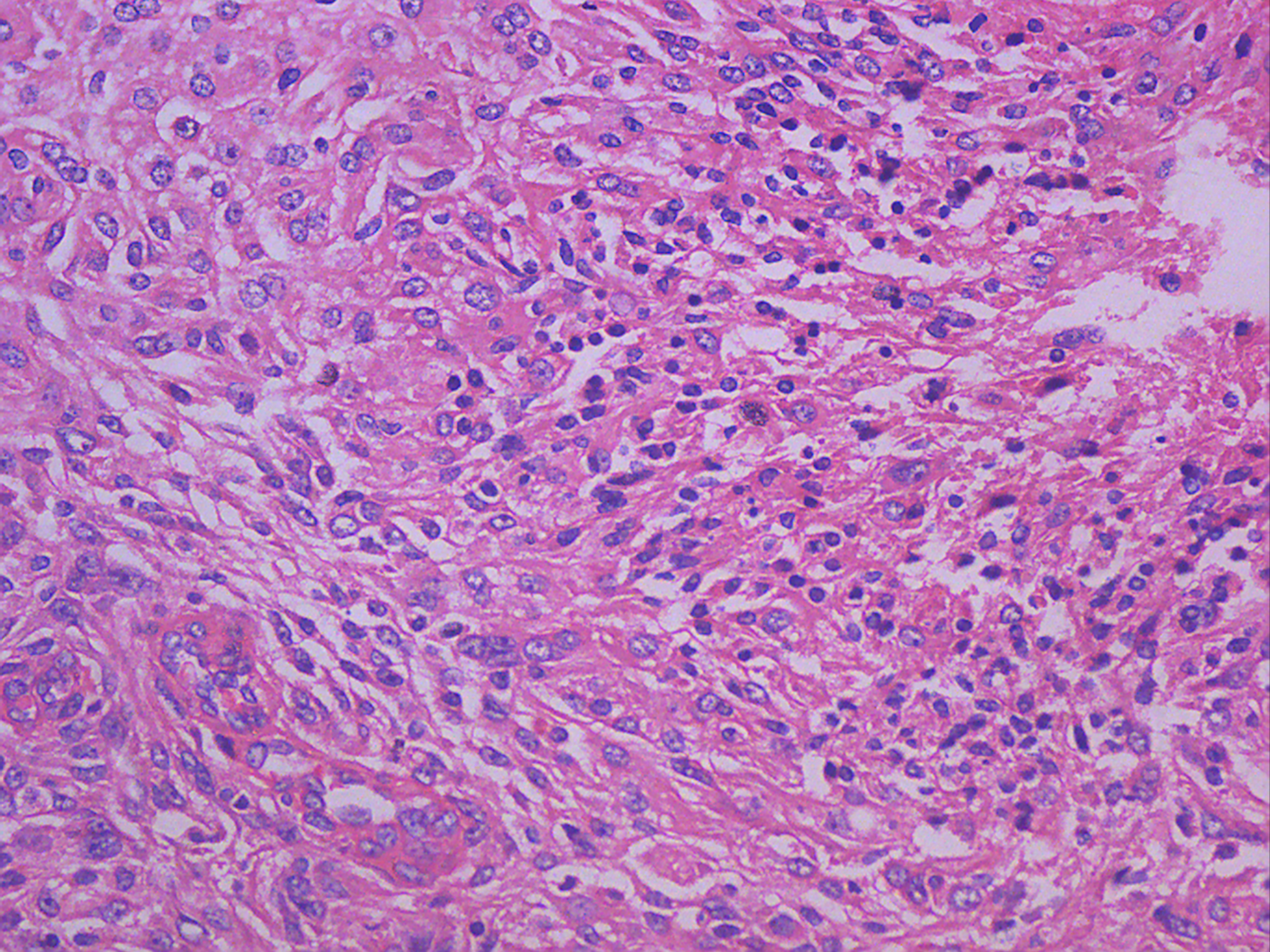

Supplement: S7 Fig — (ZIP) [file pone.0273682.s007.zip › 30.tif]

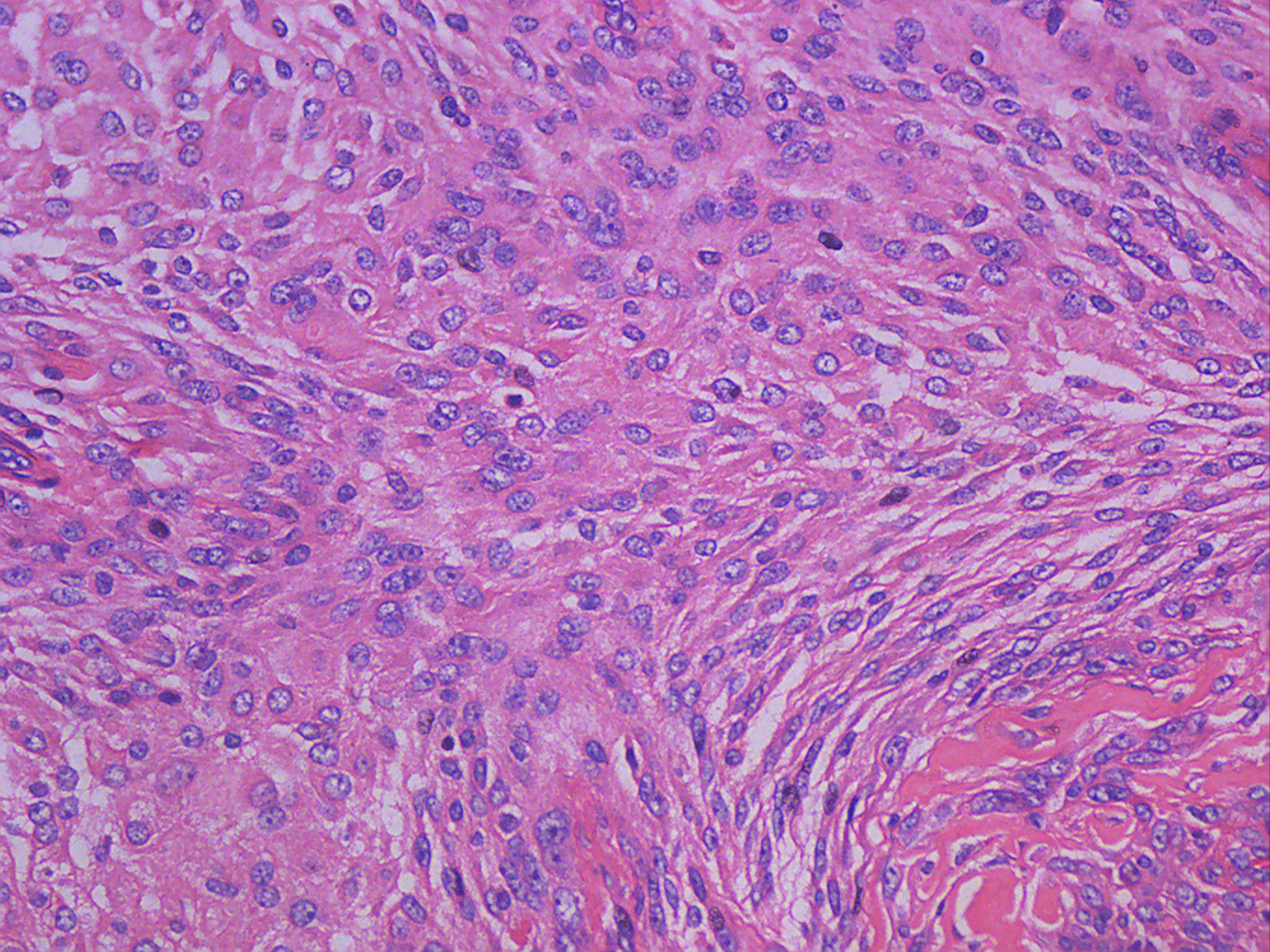

Supplement: S7 Fig — (ZIP) [file pone.0273682.s007.zip › 31.tif]

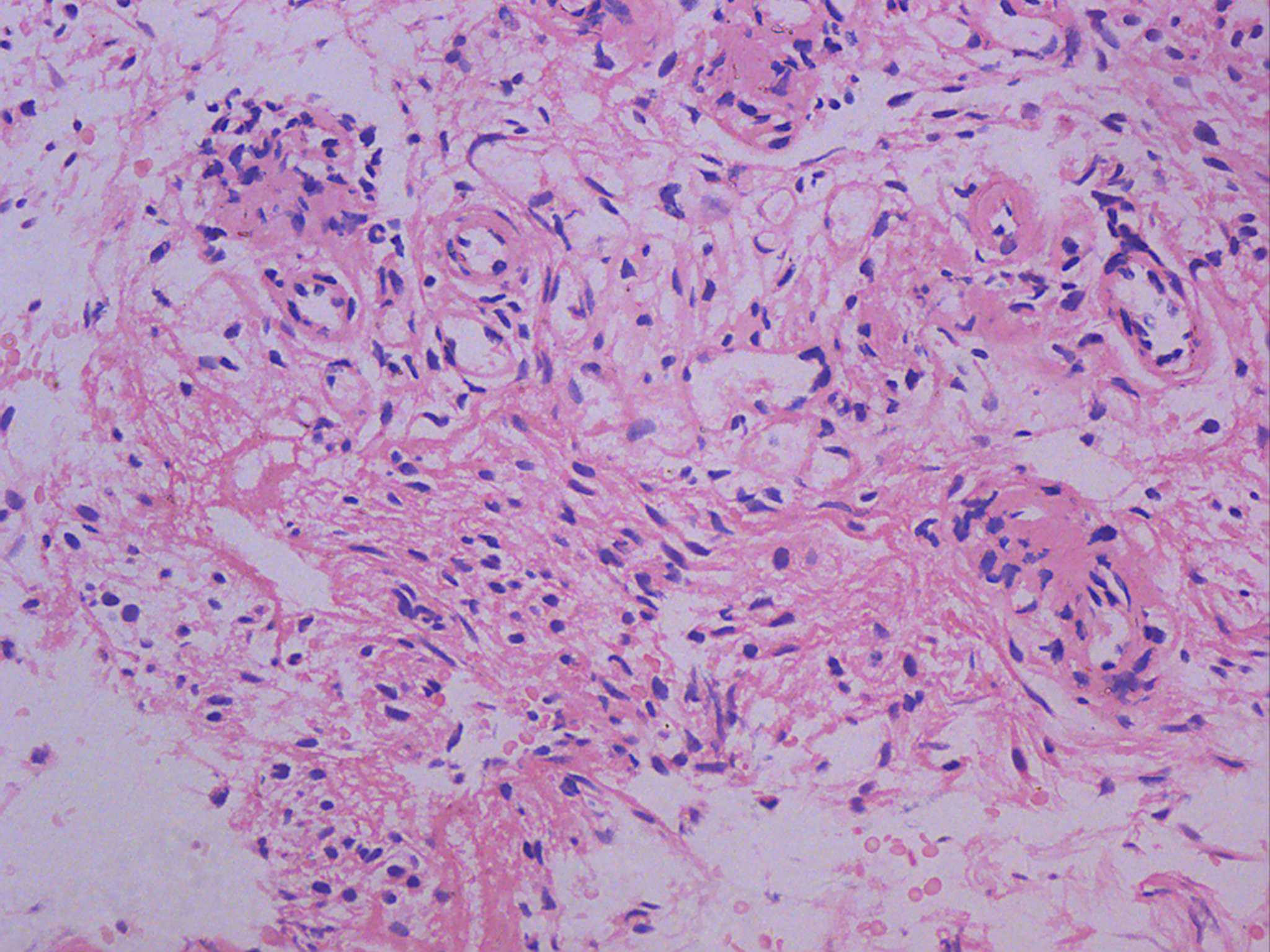

Supplement: S7 Fig — (ZIP) [file pone.0273682.s007.zip › 32.tif]

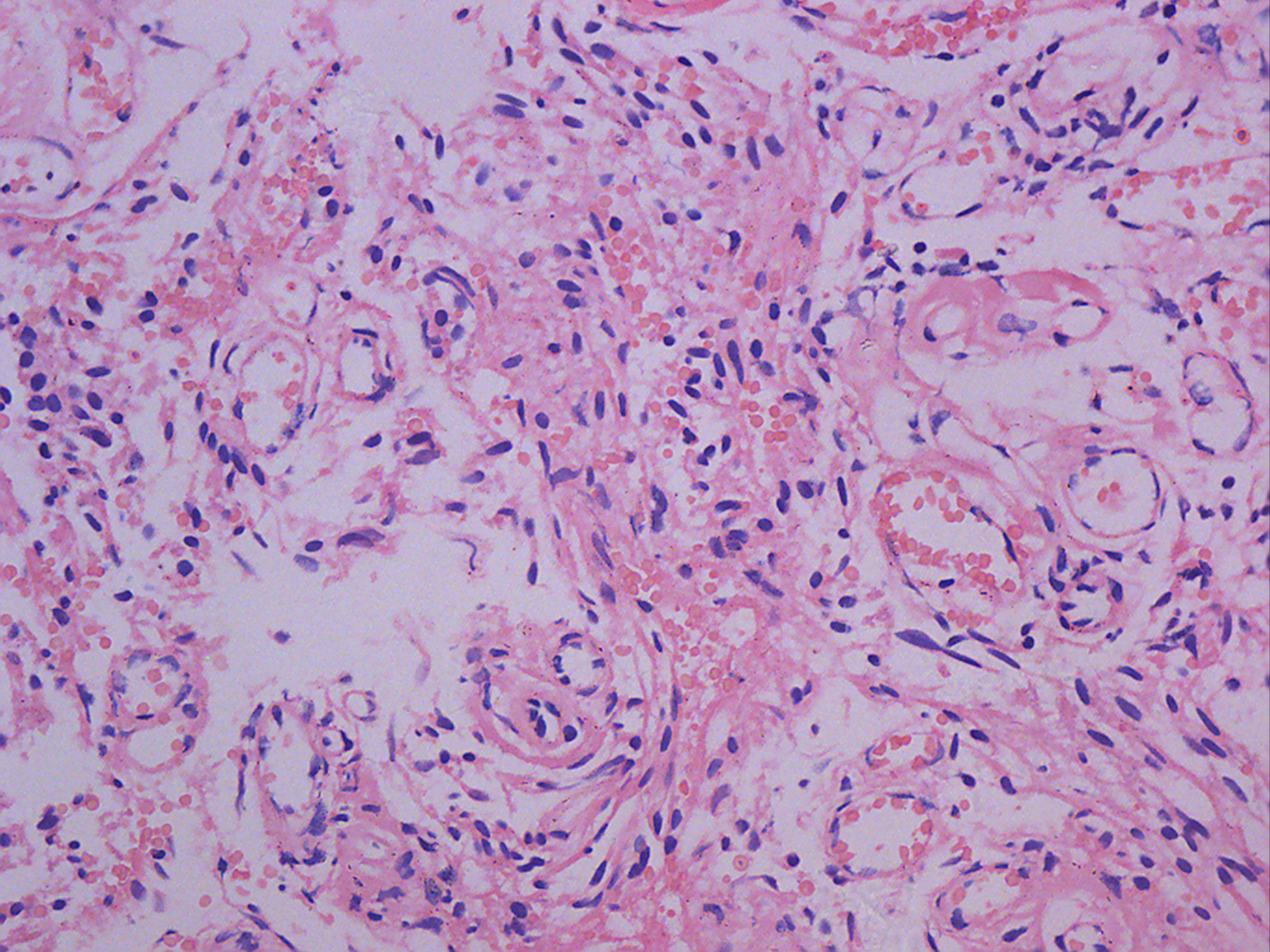

Supplement: S7 Fig — (ZIP) [file pone.0273682.s007.zip › 33.tif]

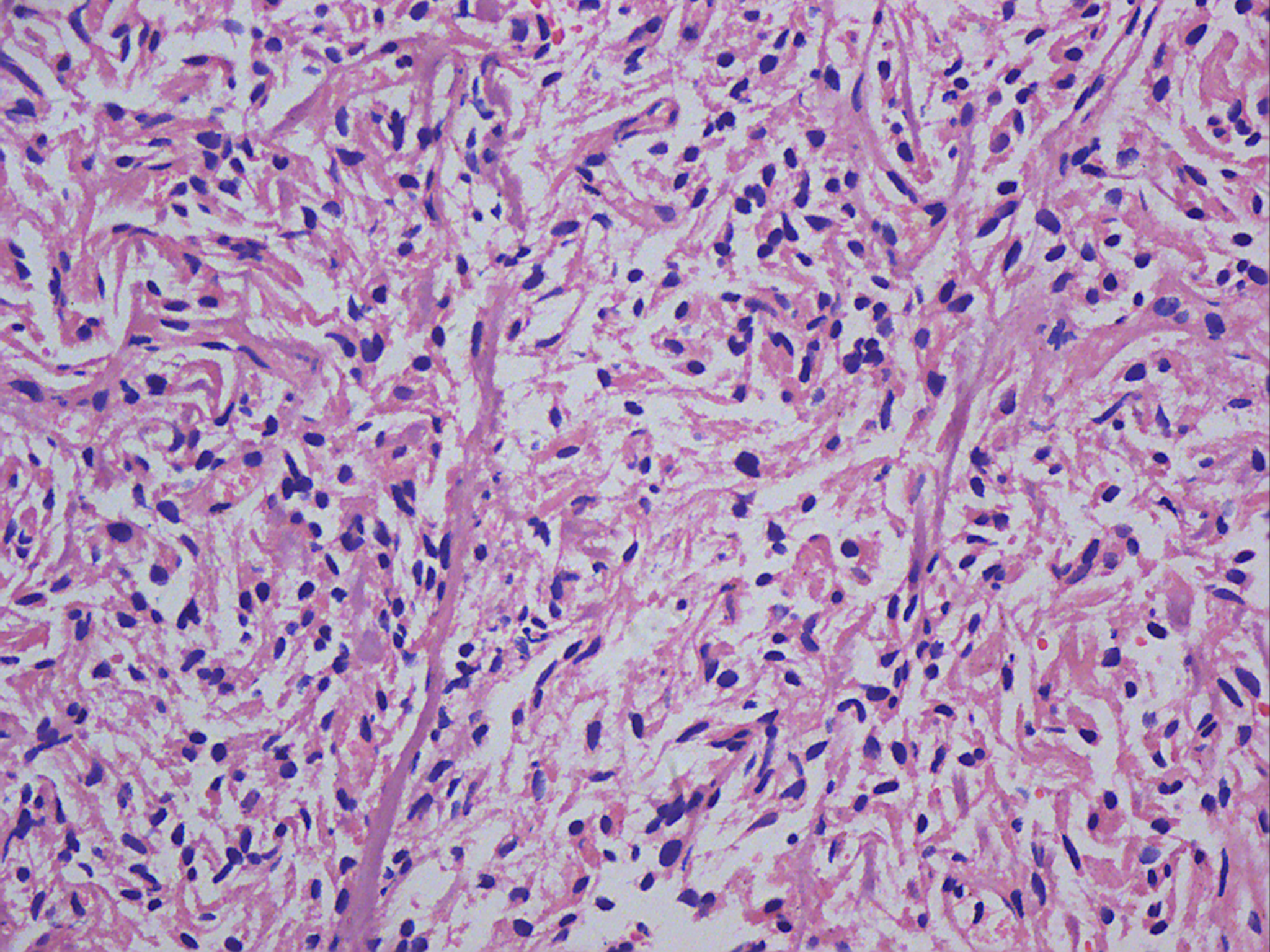

Supplement: S7 Fig — (ZIP) [file pone.0273682.s007.zip › 34.tif]

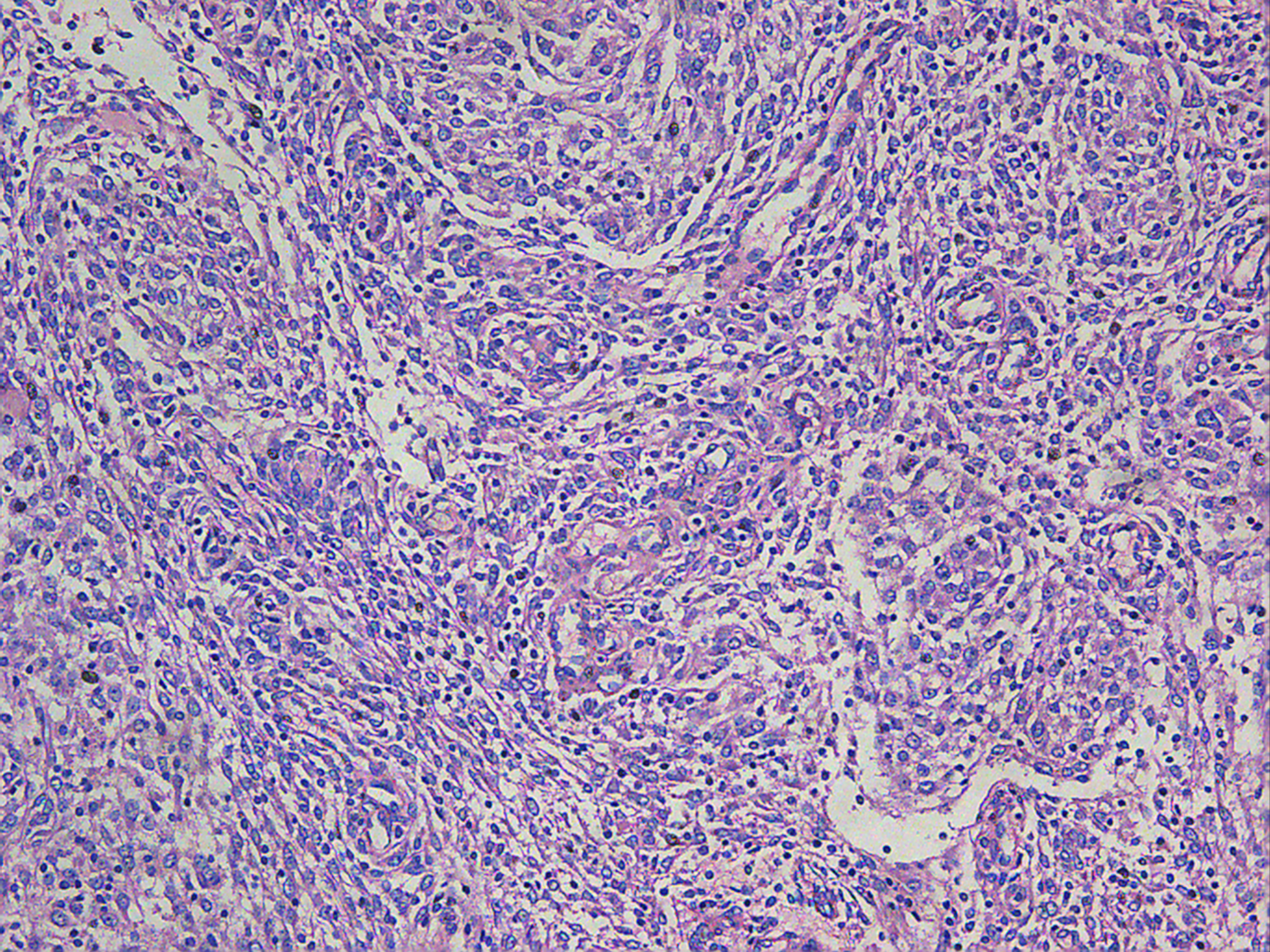

Supplement: S7 Fig — (ZIP) [file pone.0273682.s007.zip › 35.tif]

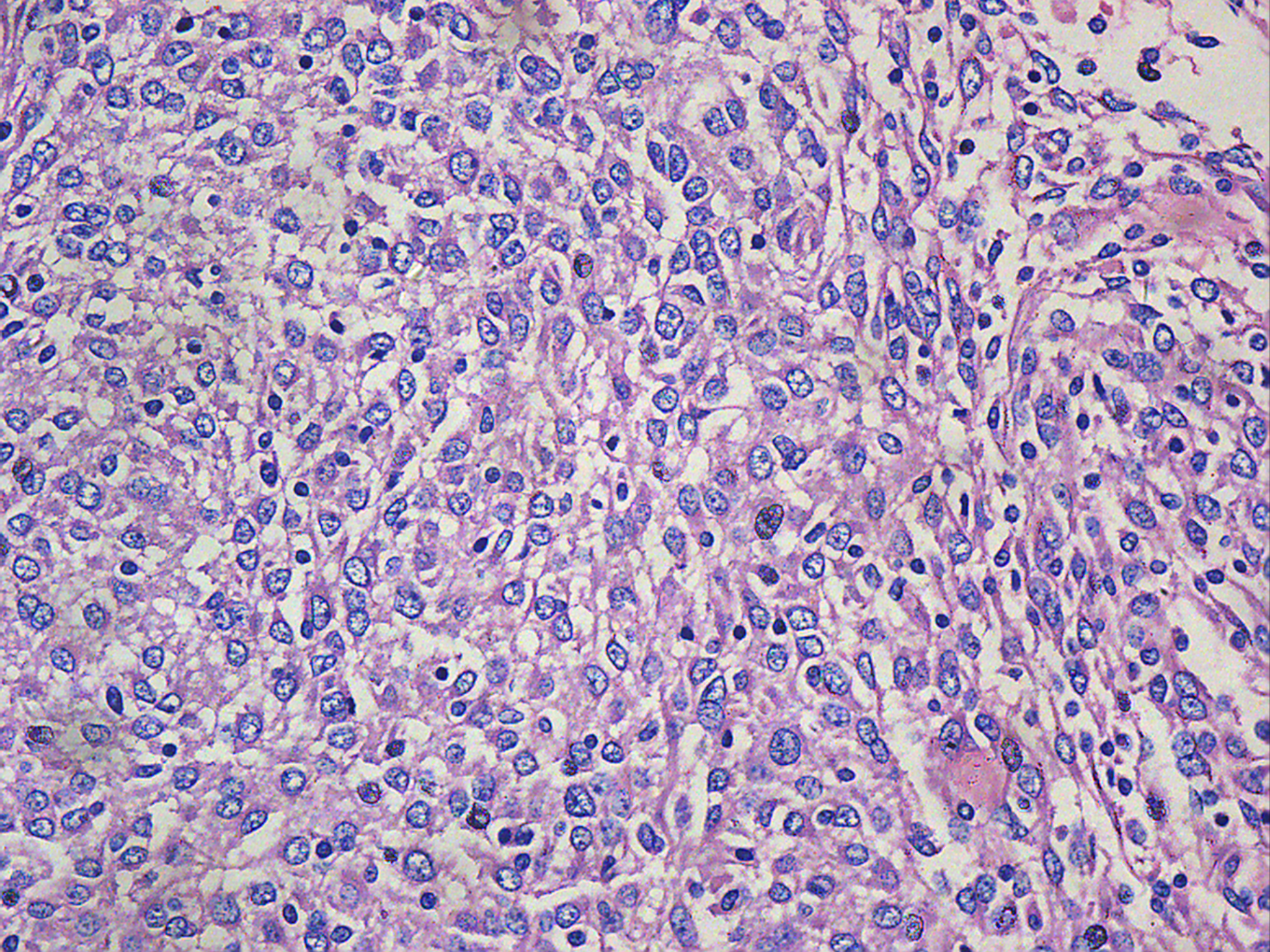

Supplement: S7 Fig — (ZIP) [file pone.0273682.s007.zip › 36.tif]

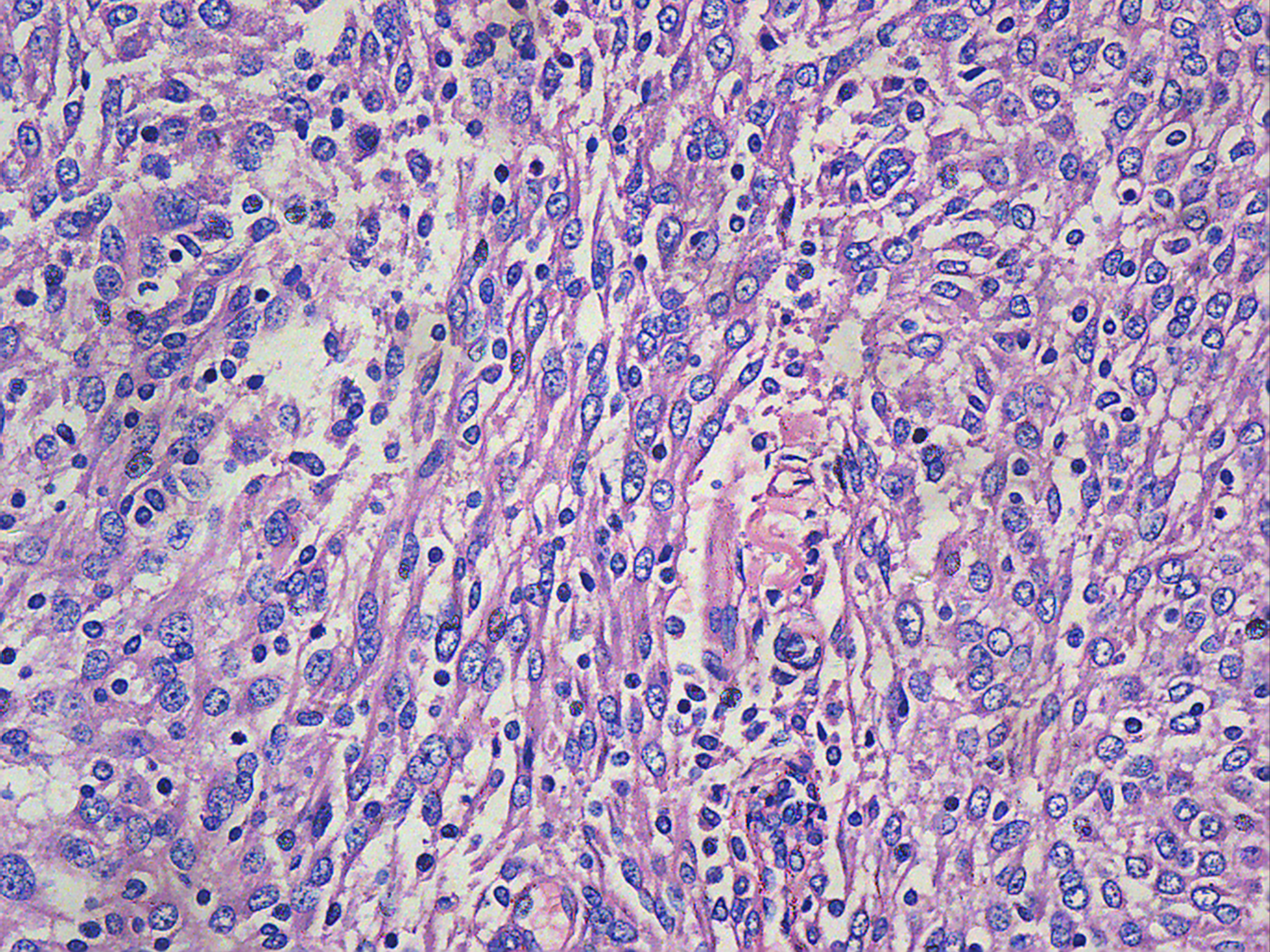

Supplement: S8 Fig — (ZIP) [file pone.0273682.s008.zip › 37.tif]

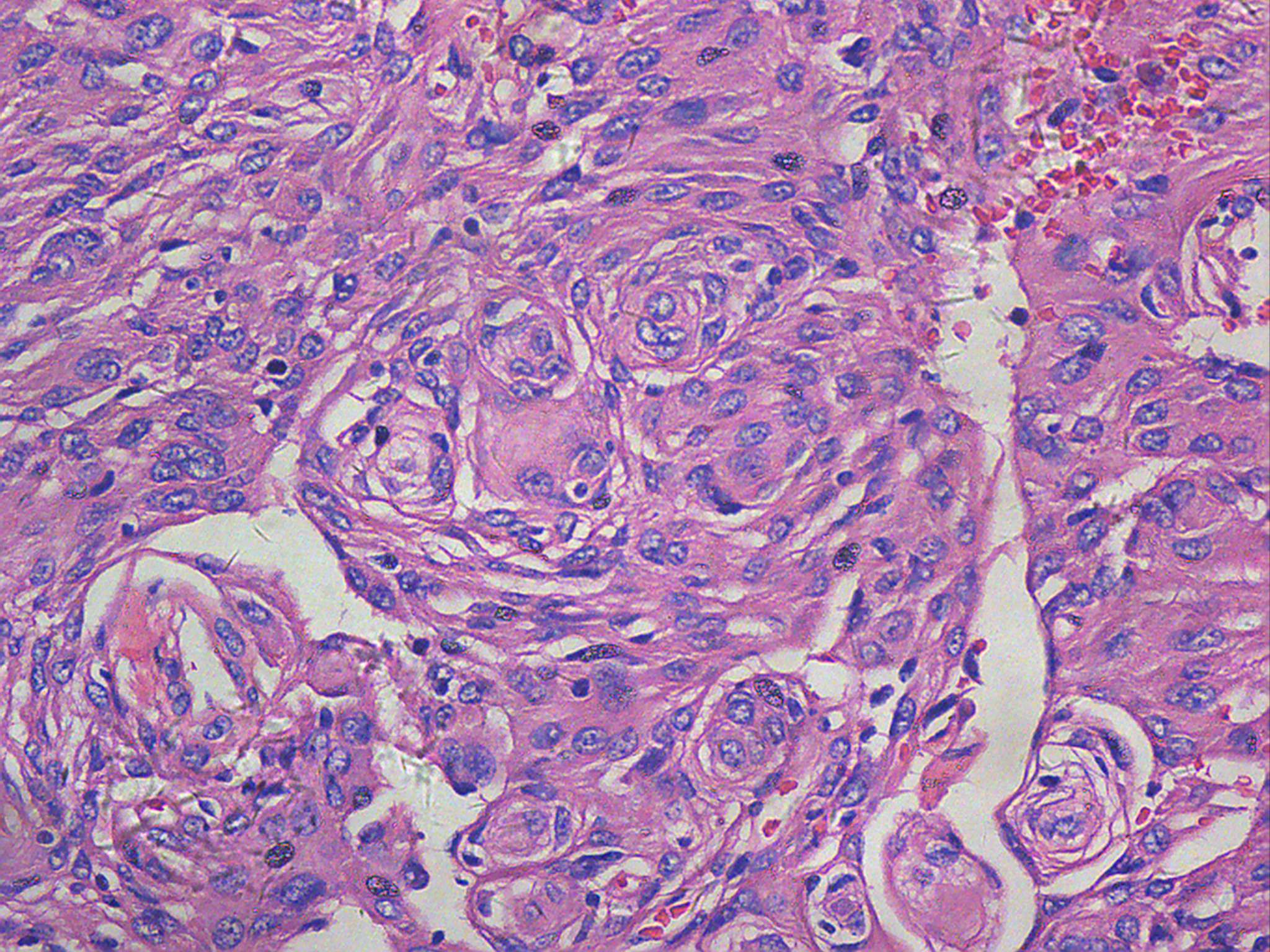

Supplement: S8 Fig — (ZIP) [file pone.0273682.s008.zip › 38.tif]

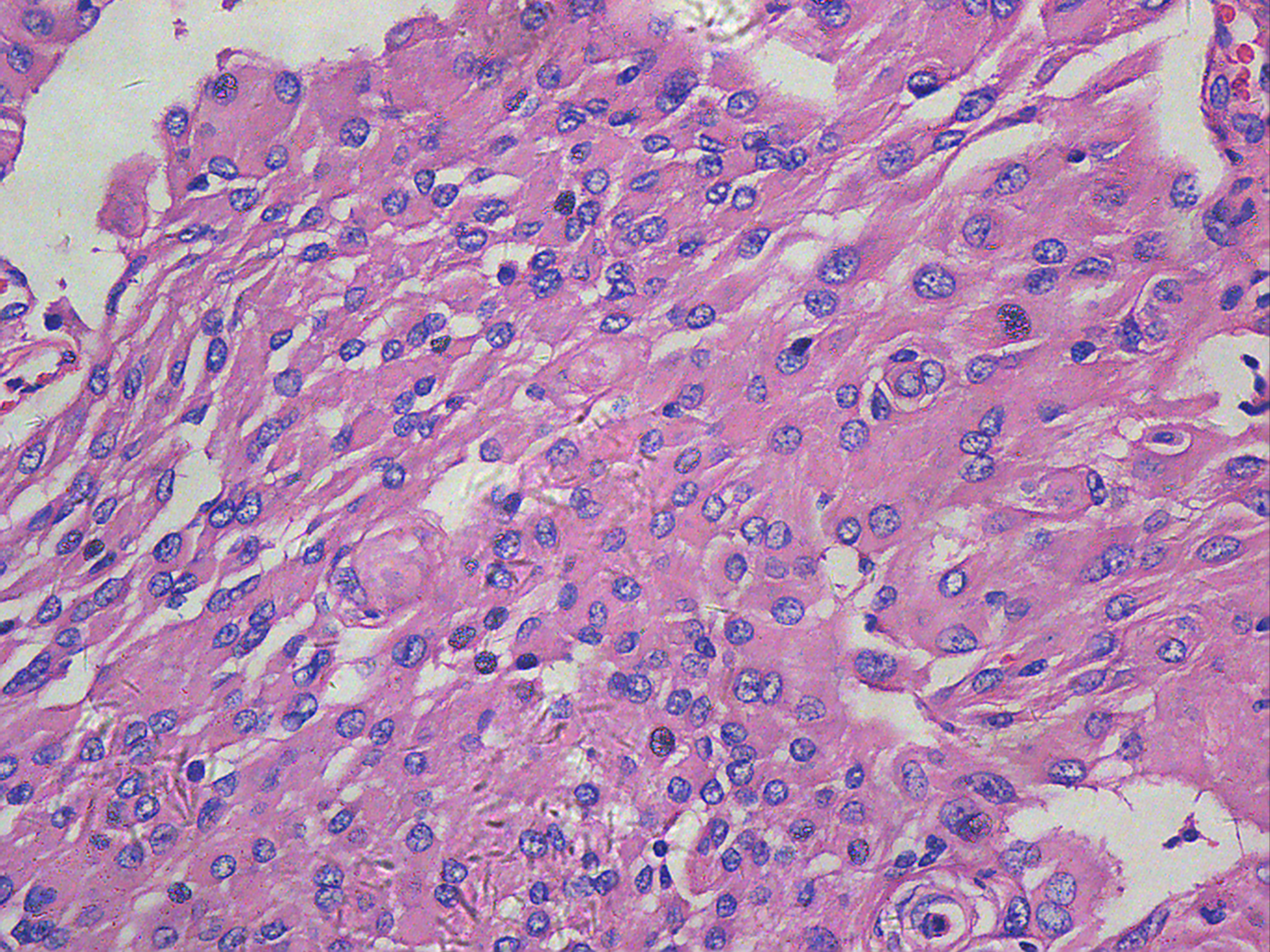

Supplement: S8 Fig — (ZIP) [file pone.0273682.s008.zip › 39.tif]

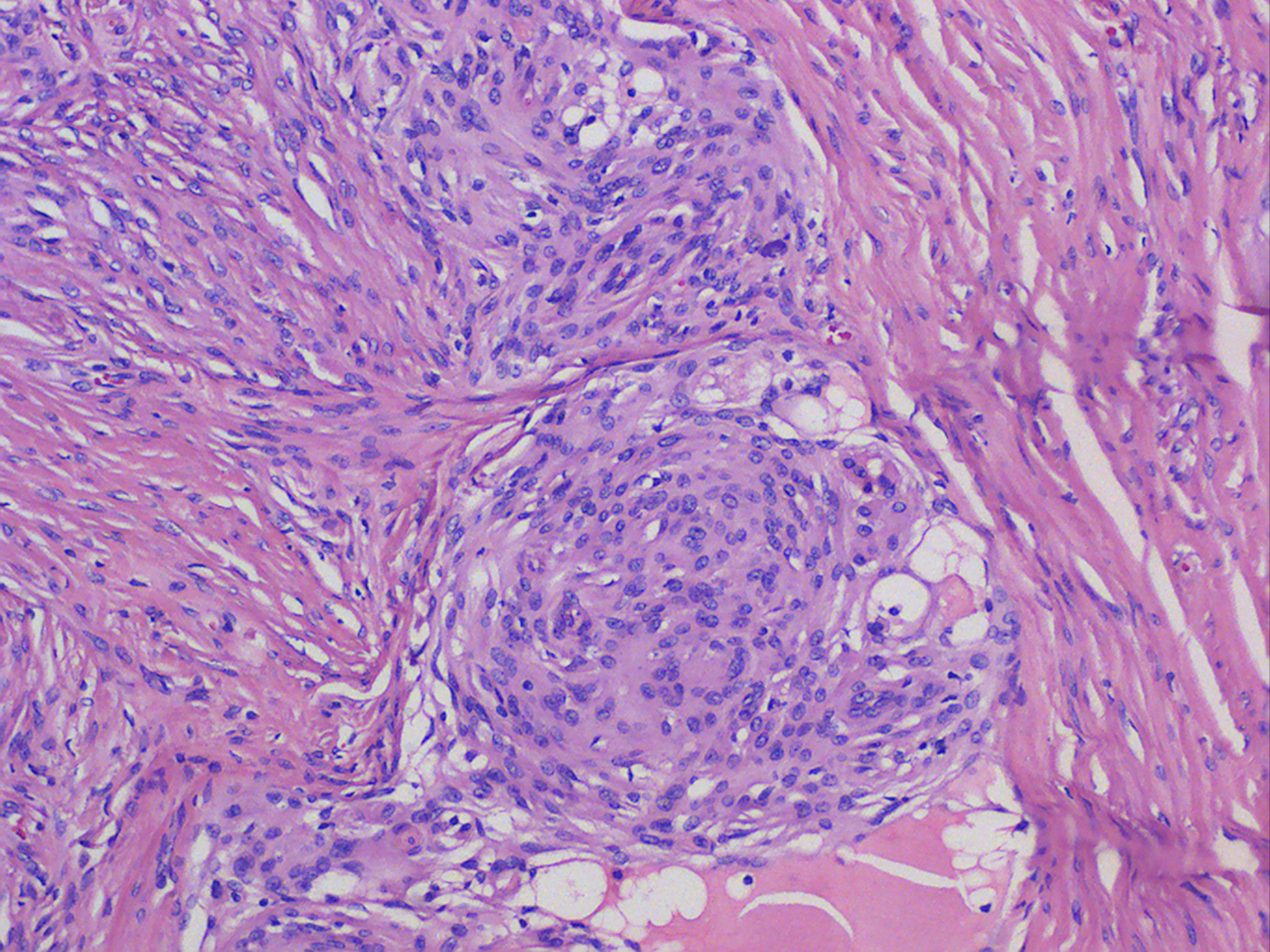

Supplement: S8 Fig — (ZIP) [file pone.0273682.s008.zip › 40.tif]

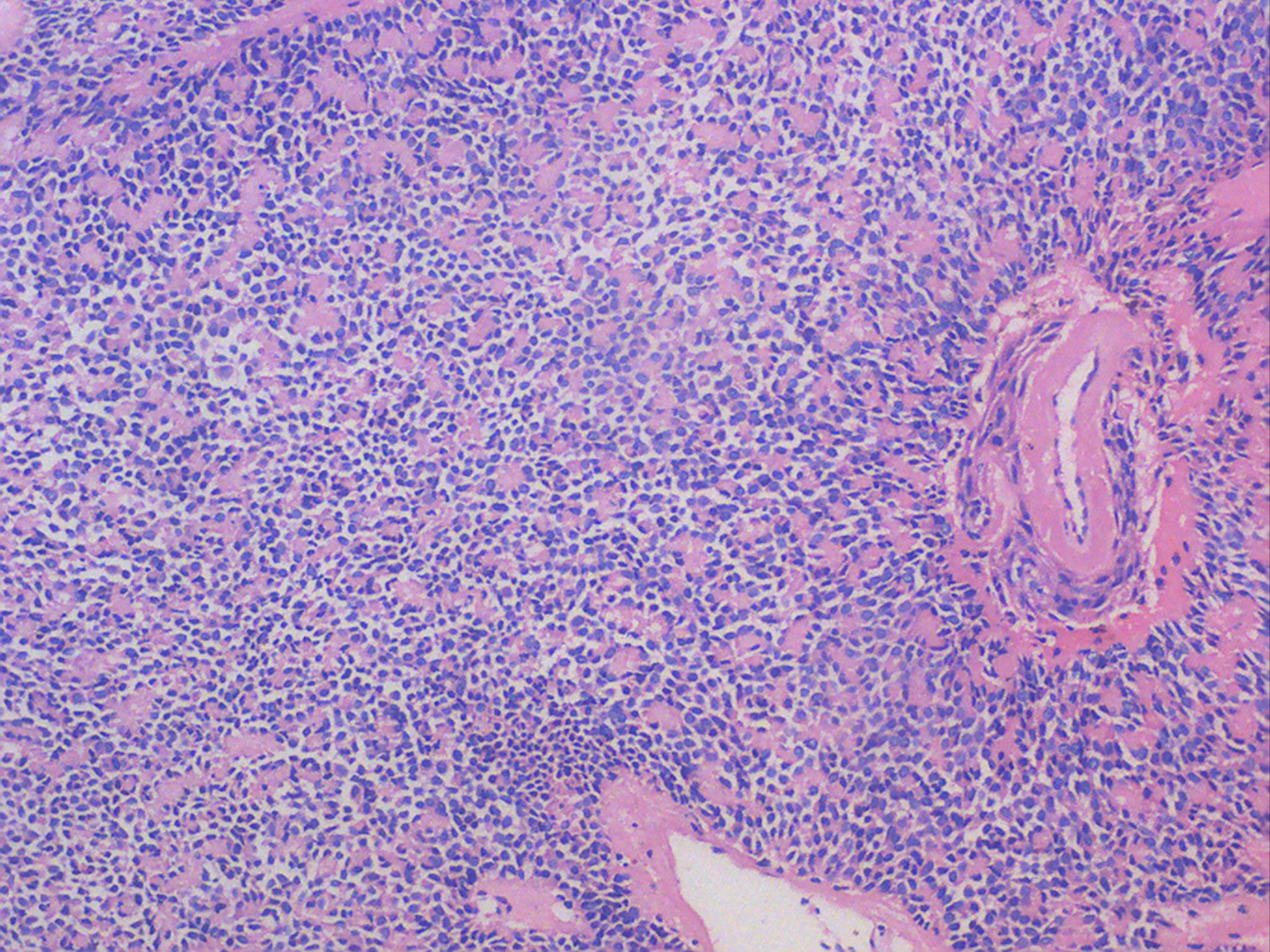

Supplement: S8 Fig — (ZIP) [file pone.0273682.s008.zip › 42.tif]

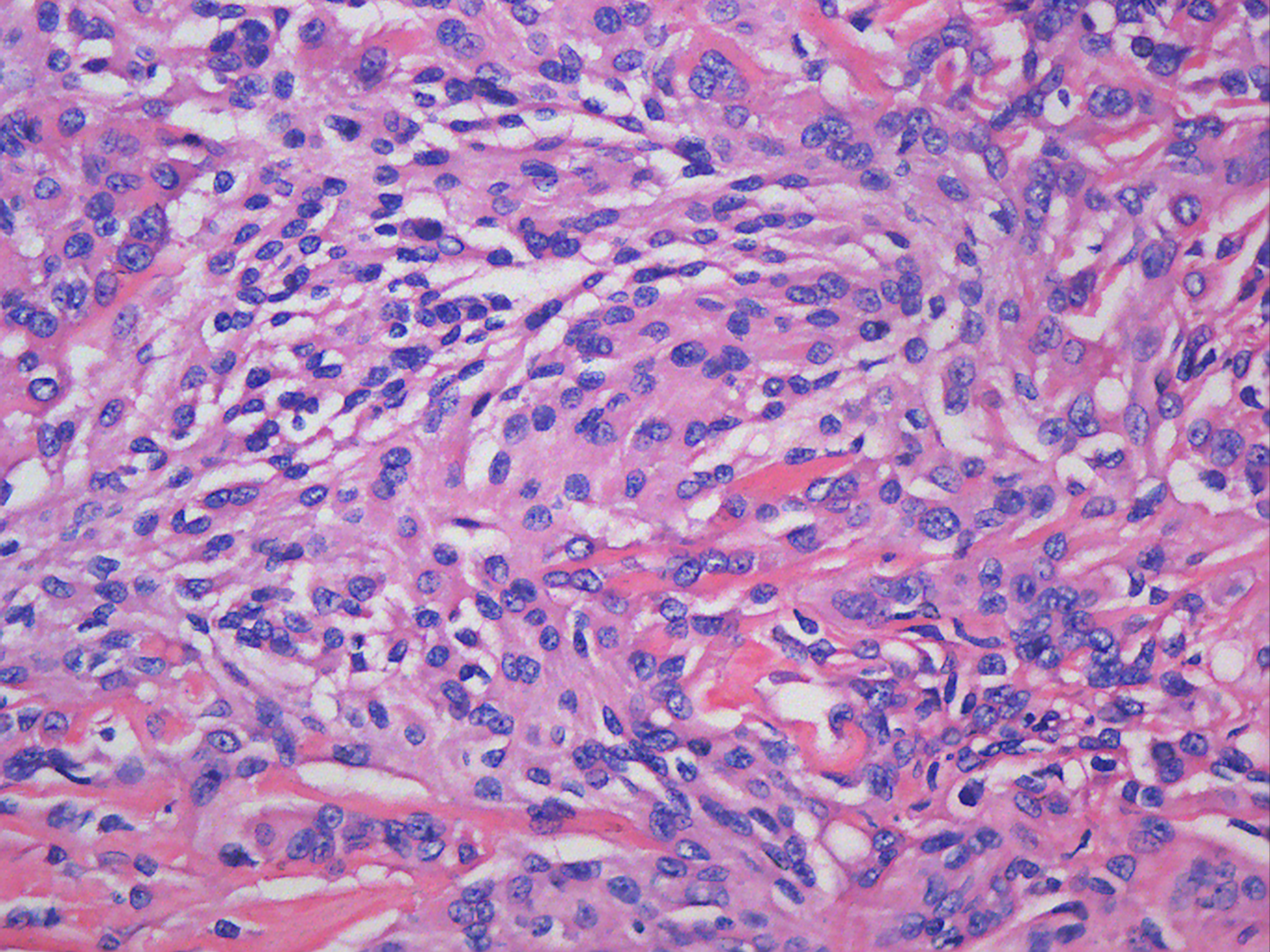

Supplement: S8 Fig — (ZIP) [file pone.0273682.s008.zip › 43.tif]

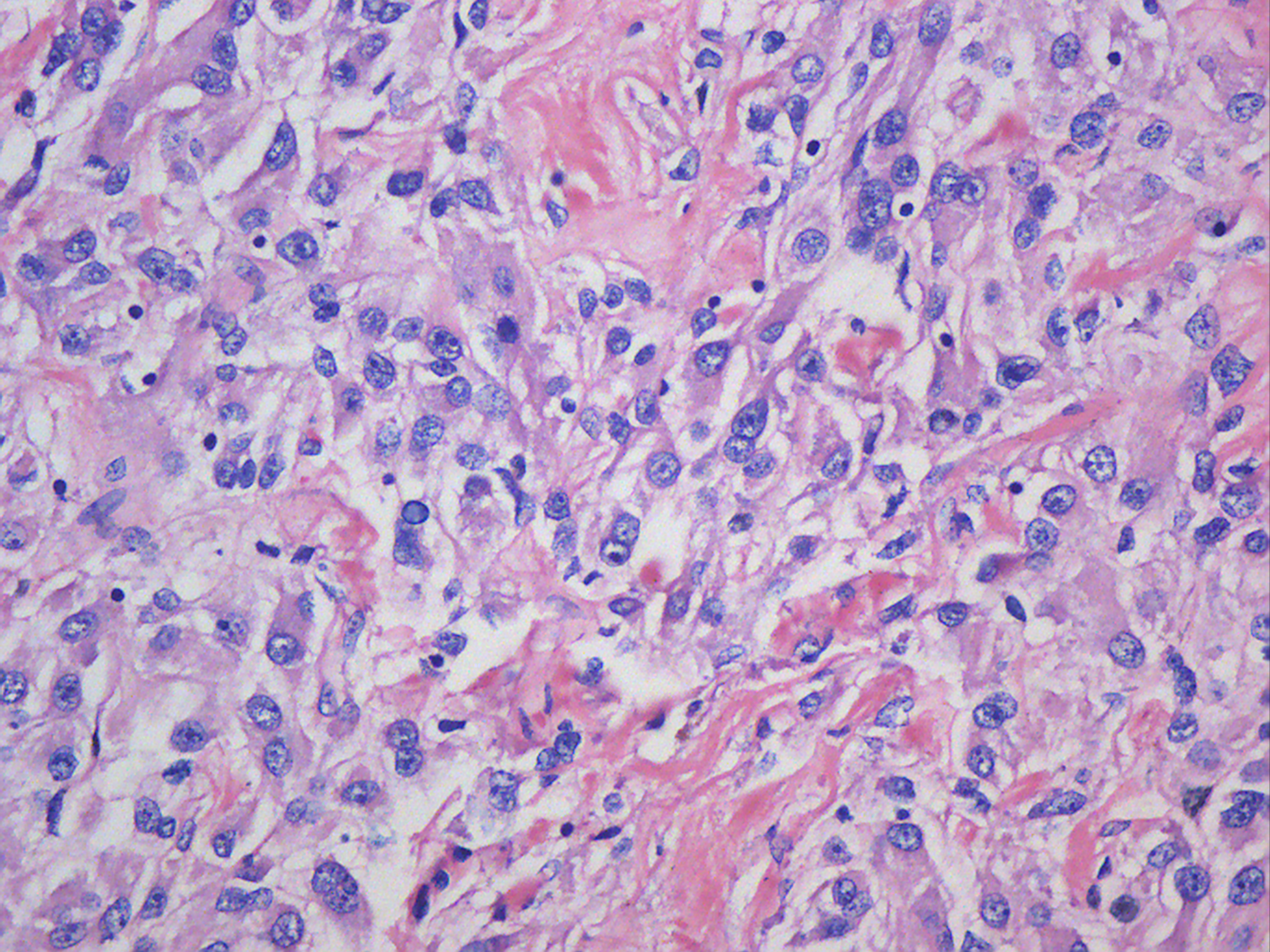

Supplement: S8 Fig — (ZIP) [file pone.0273682.s008.zip › 44.tif]

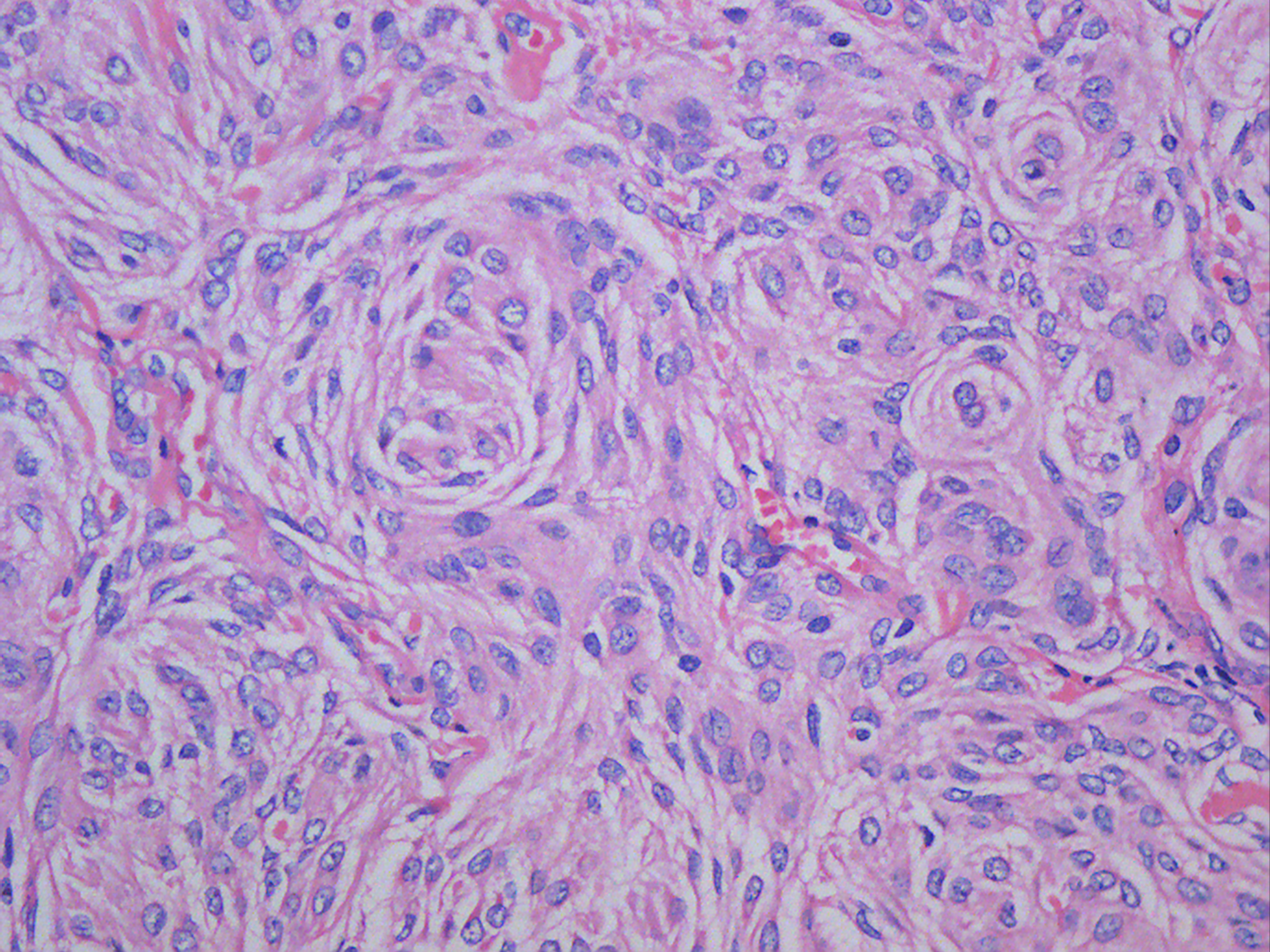

Supplement: S8 Fig — (ZIP) [file pone.0273682.s008.zip › 45.tif]

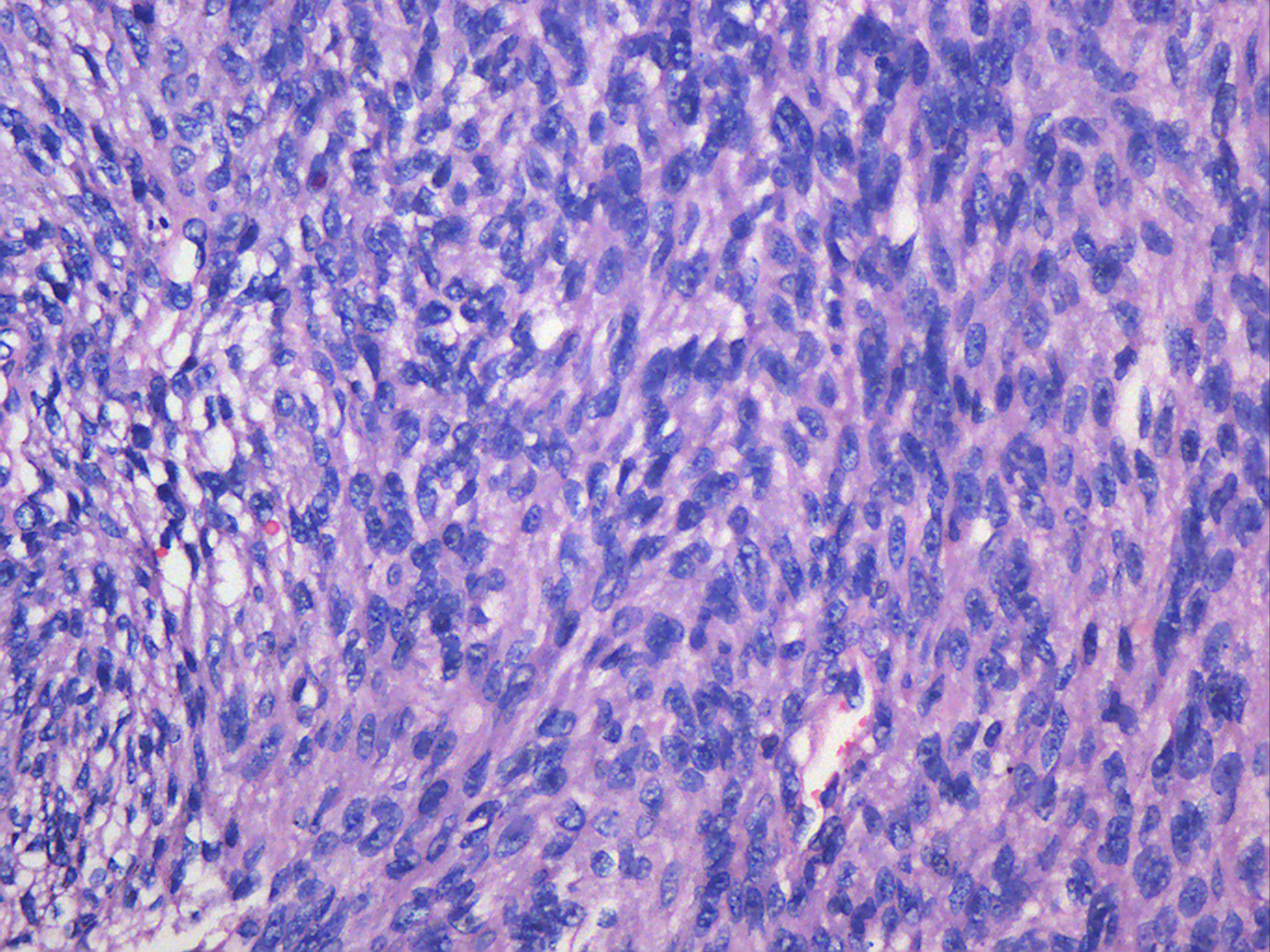

Supplement: S8 Fig — (ZIP) [file pone.0273682.s008.zip › 46.tif]

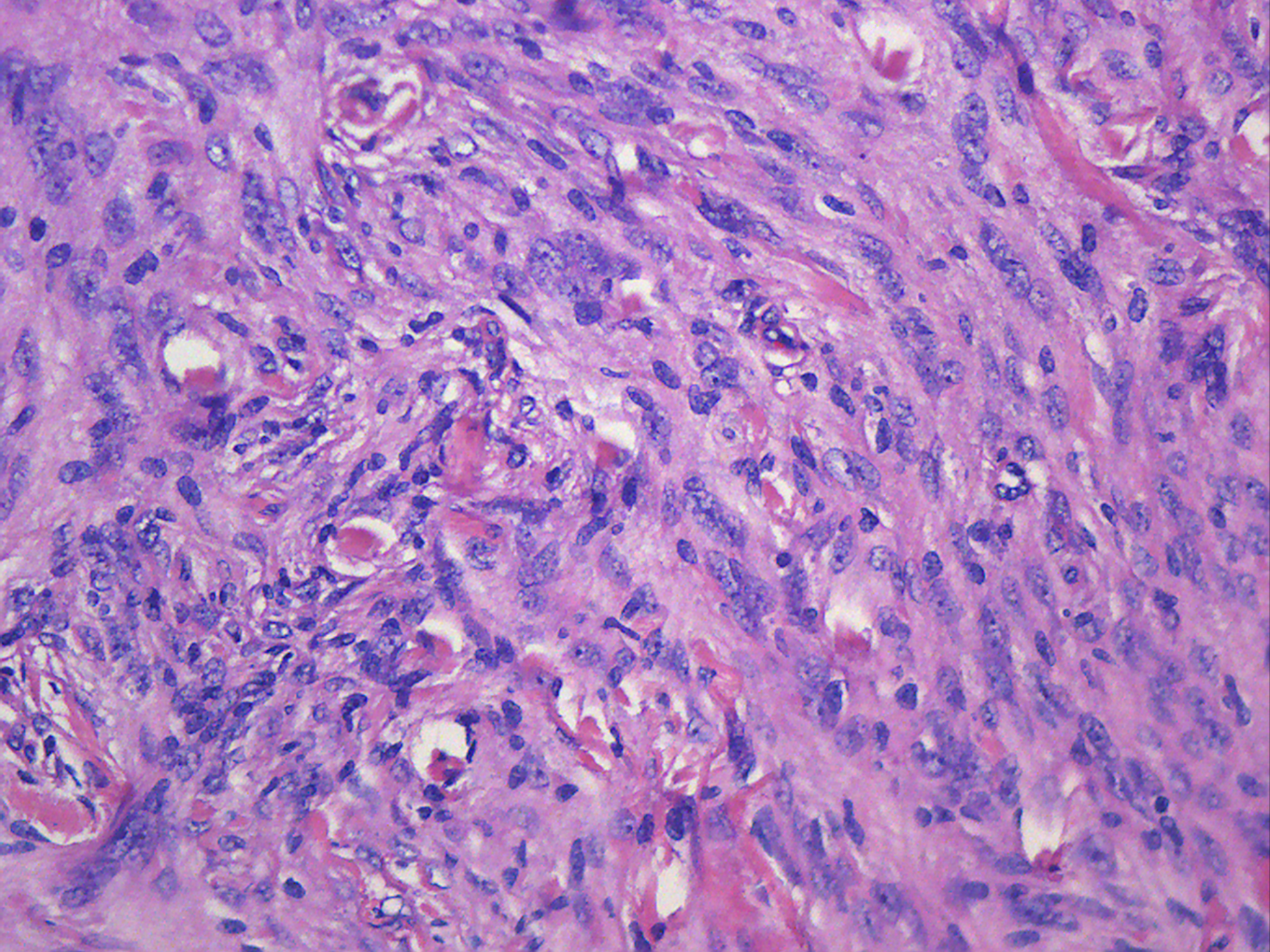

Supplement: S8 Fig — (ZIP) [file pone.0273682.s008.zip › 47.tif]

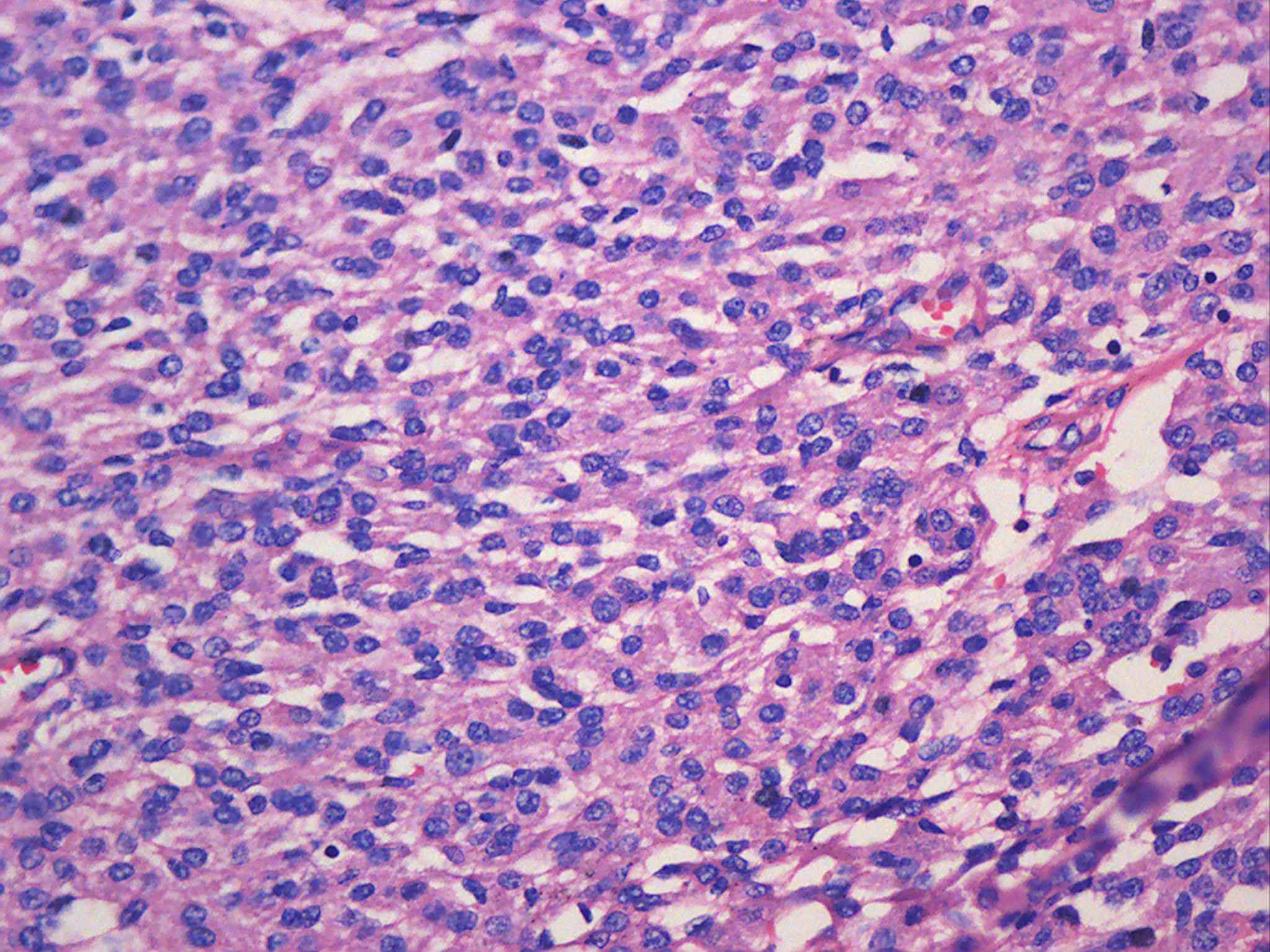

Supplement: S8 Fig — (ZIP) [file pone.0273682.s008.zip › 48.tif]

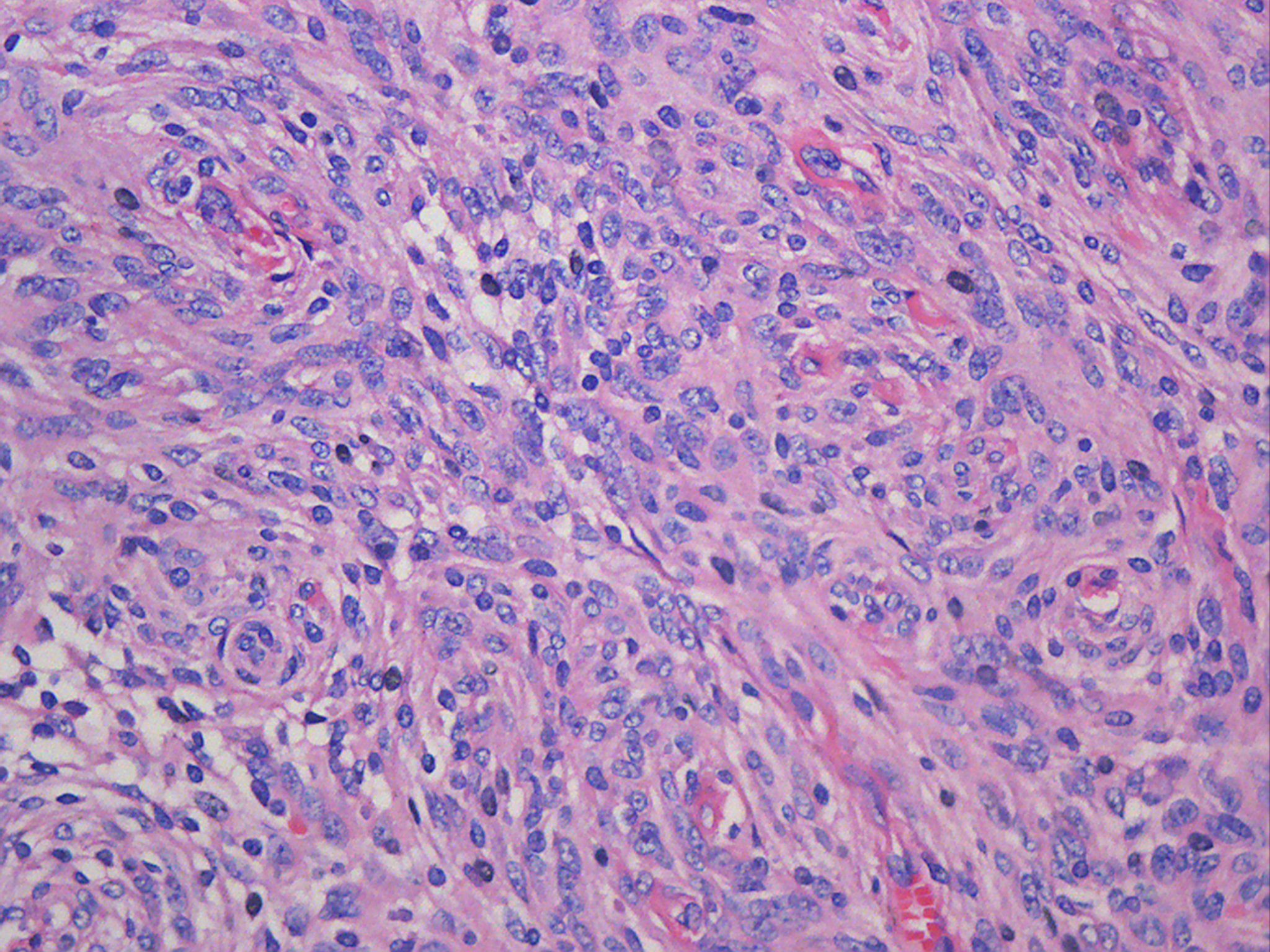

Supplement: S9 Fig — (ZIP) [file pone.0273682.s009.zip › 49.tif]

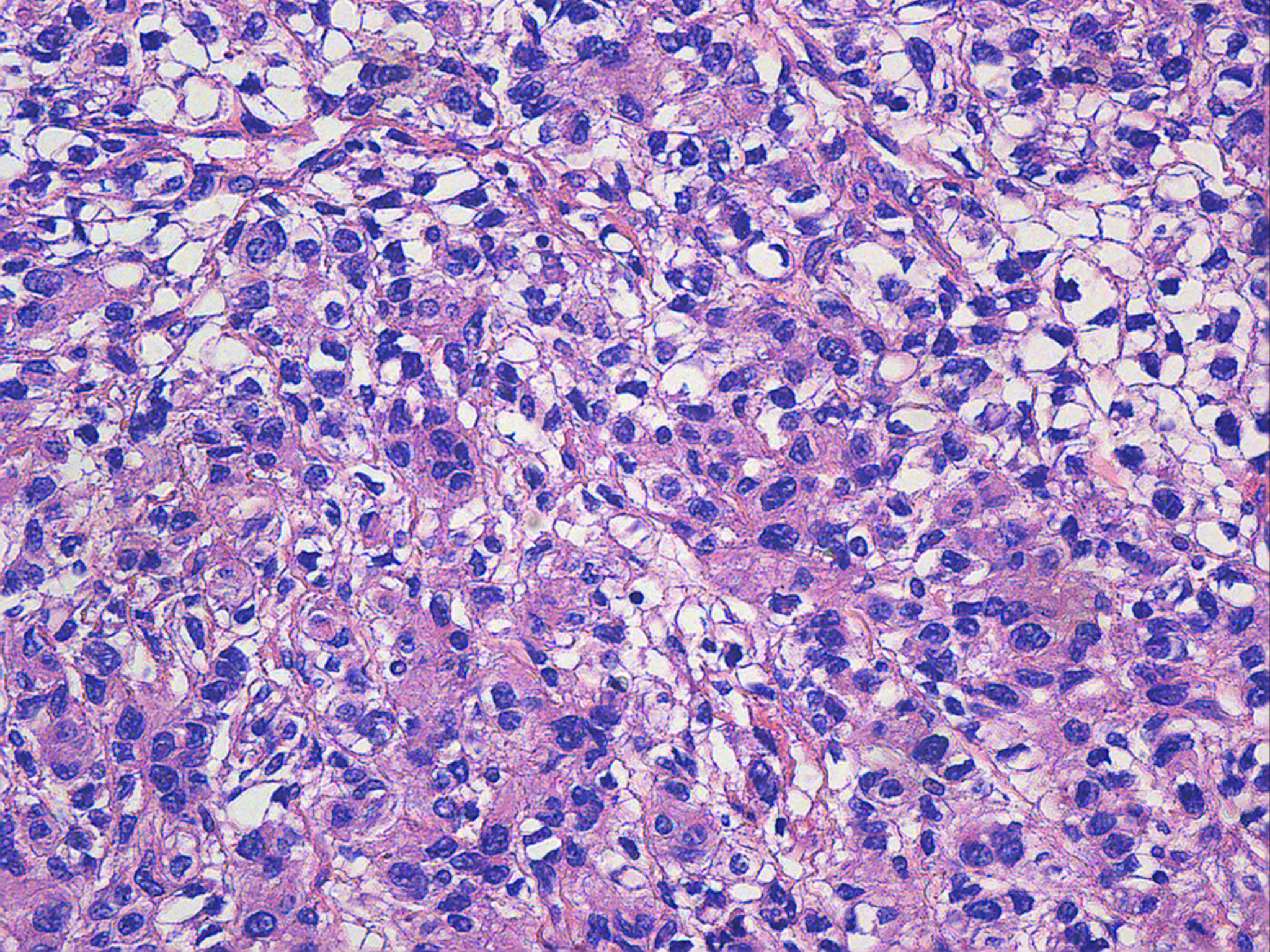

Supplement: S9 Fig — (ZIP) [file pone.0273682.s009.zip › 50.tif]

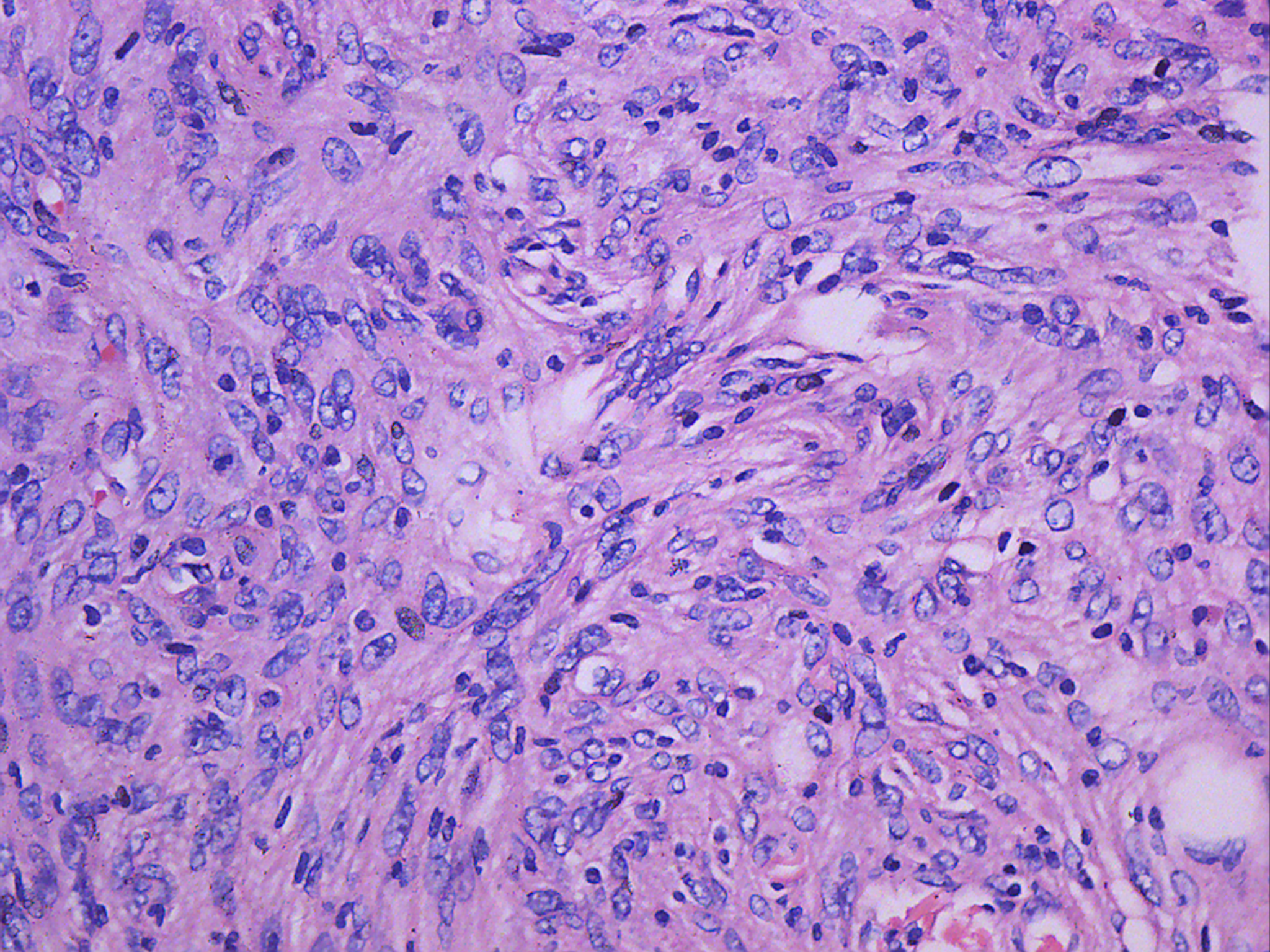

Supplement: S9 Fig — (ZIP) [file pone.0273682.s009.zip › 52.tif]

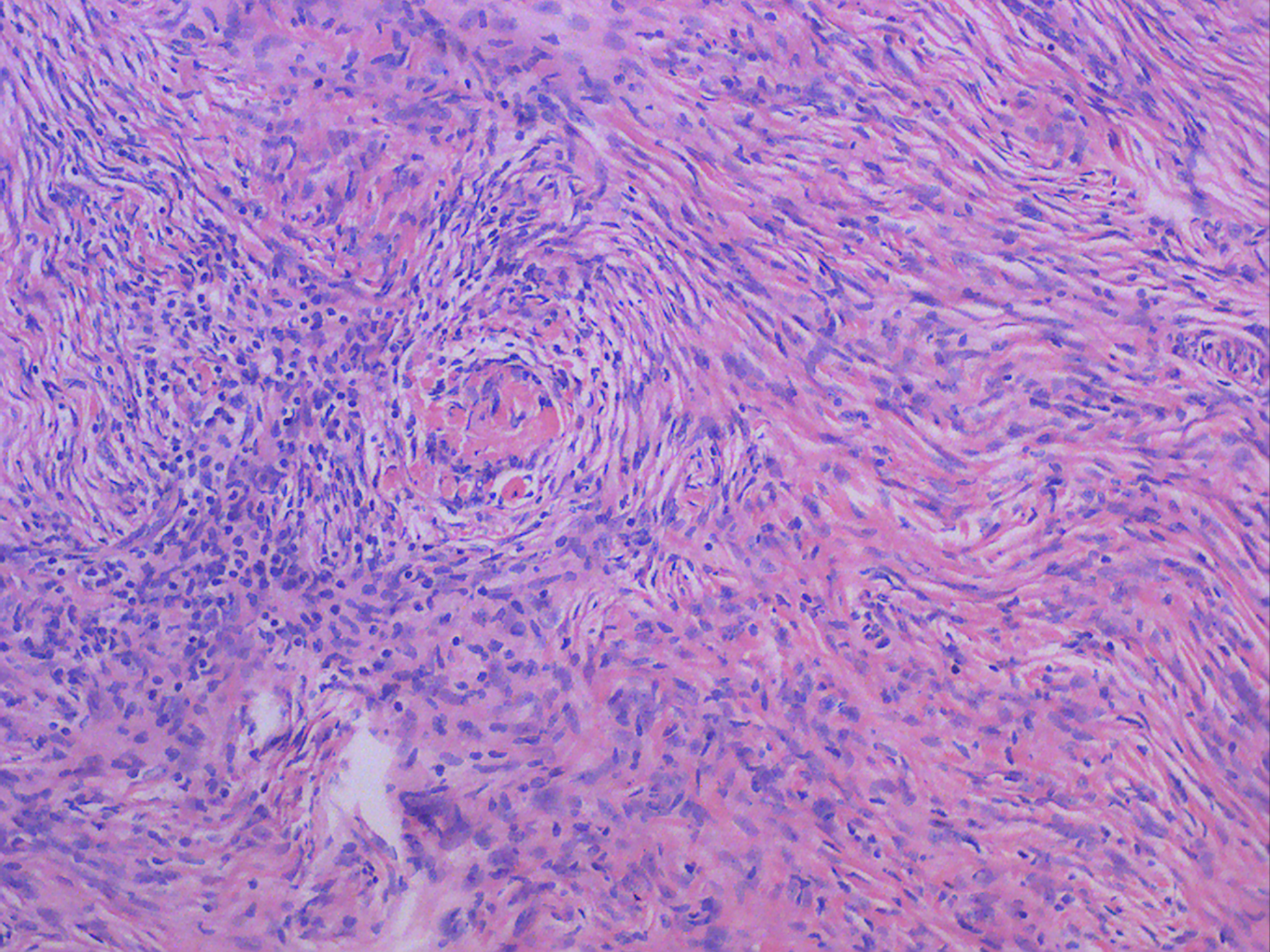

Supplement: S9 Fig — (ZIP) [file pone.0273682.s009.zip › 53.tif]

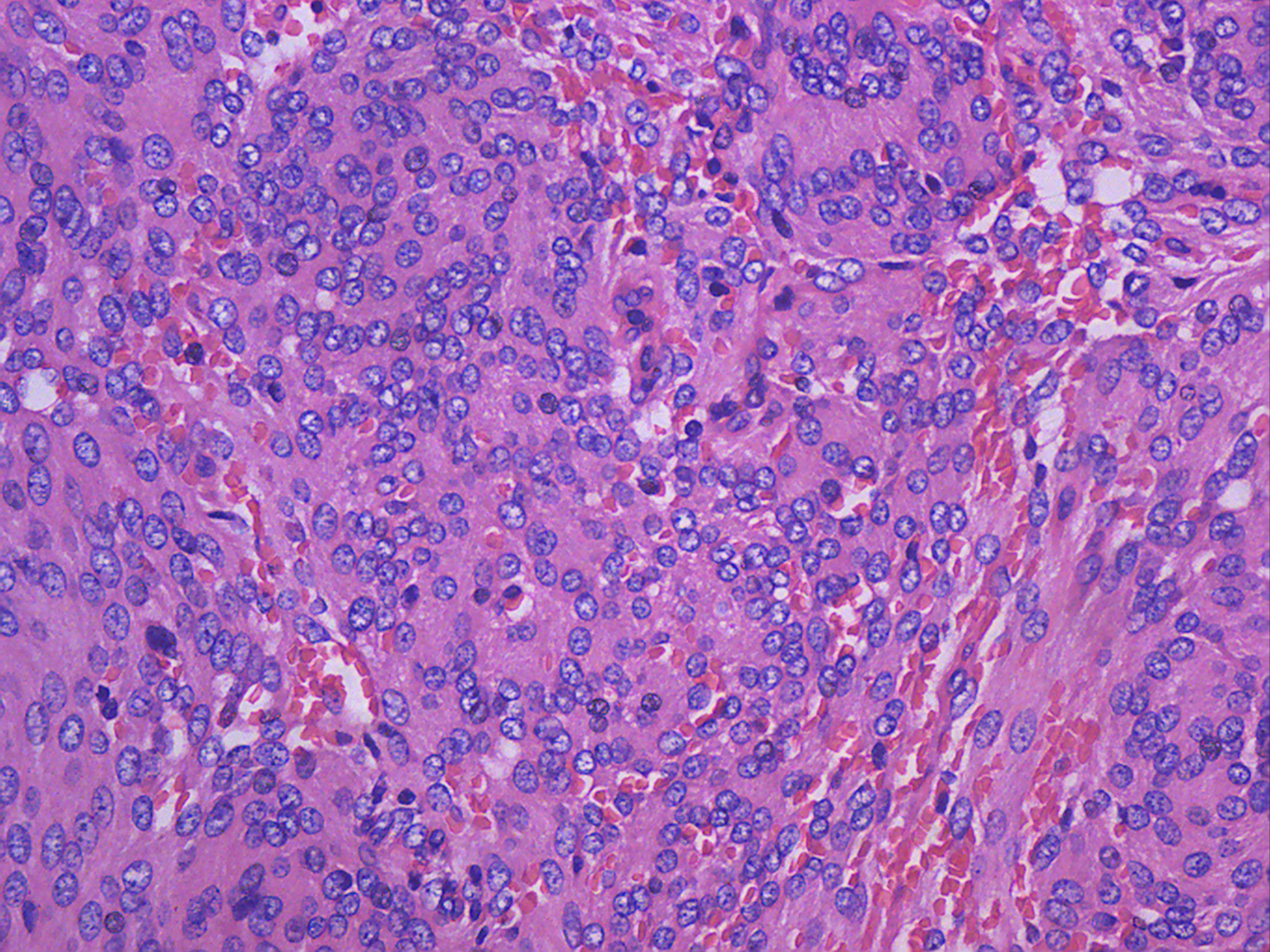

Supplement: S9 Fig — (ZIP) [file pone.0273682.s009.zip › 54.tif]

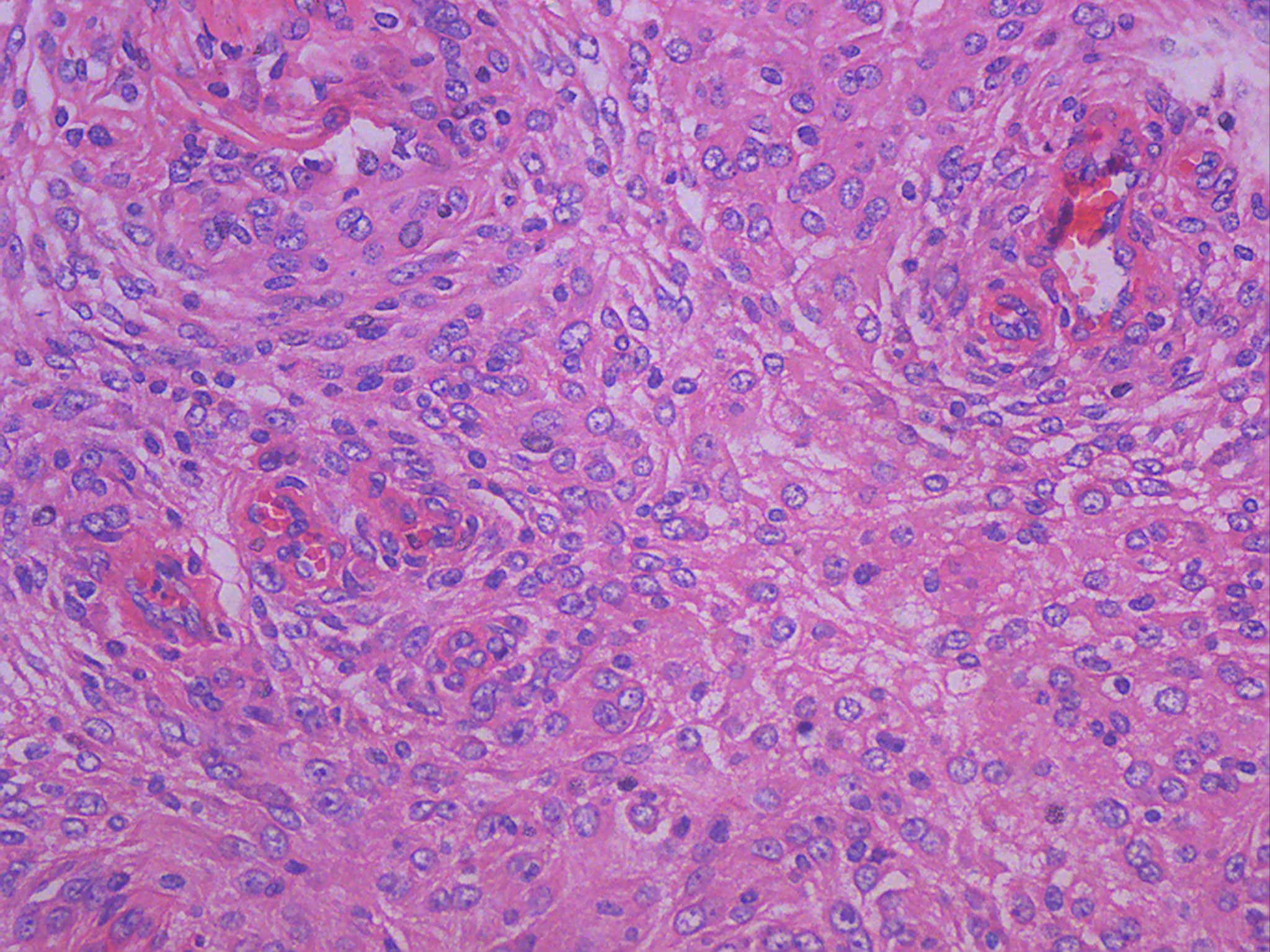

Supplement: S9 Fig — (ZIP) [file pone.0273682.s009.zip › 55.tif]

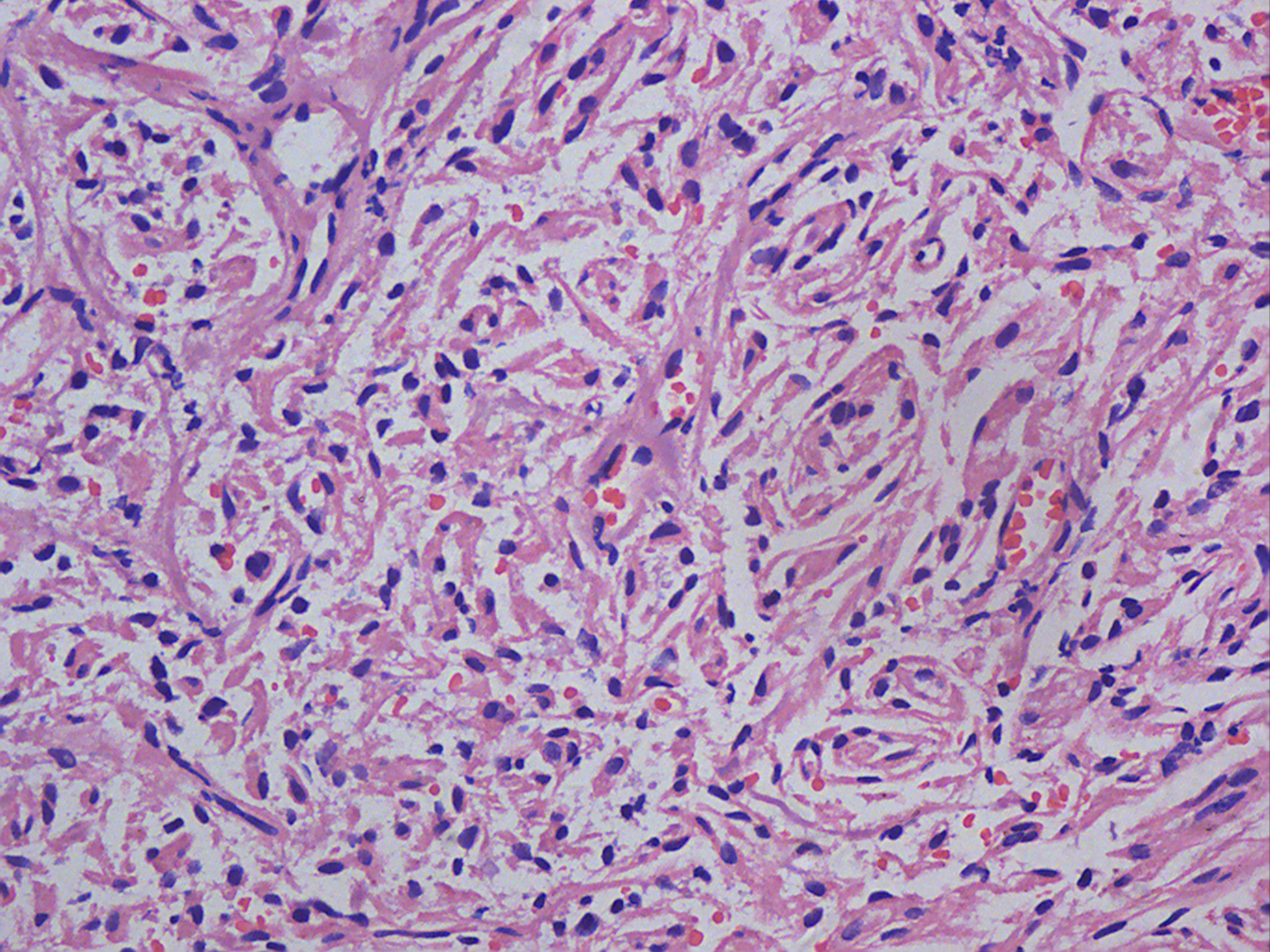

Supplement: S9 Fig — (ZIP) [file pone.0273682.s009.zip › 56.tif]

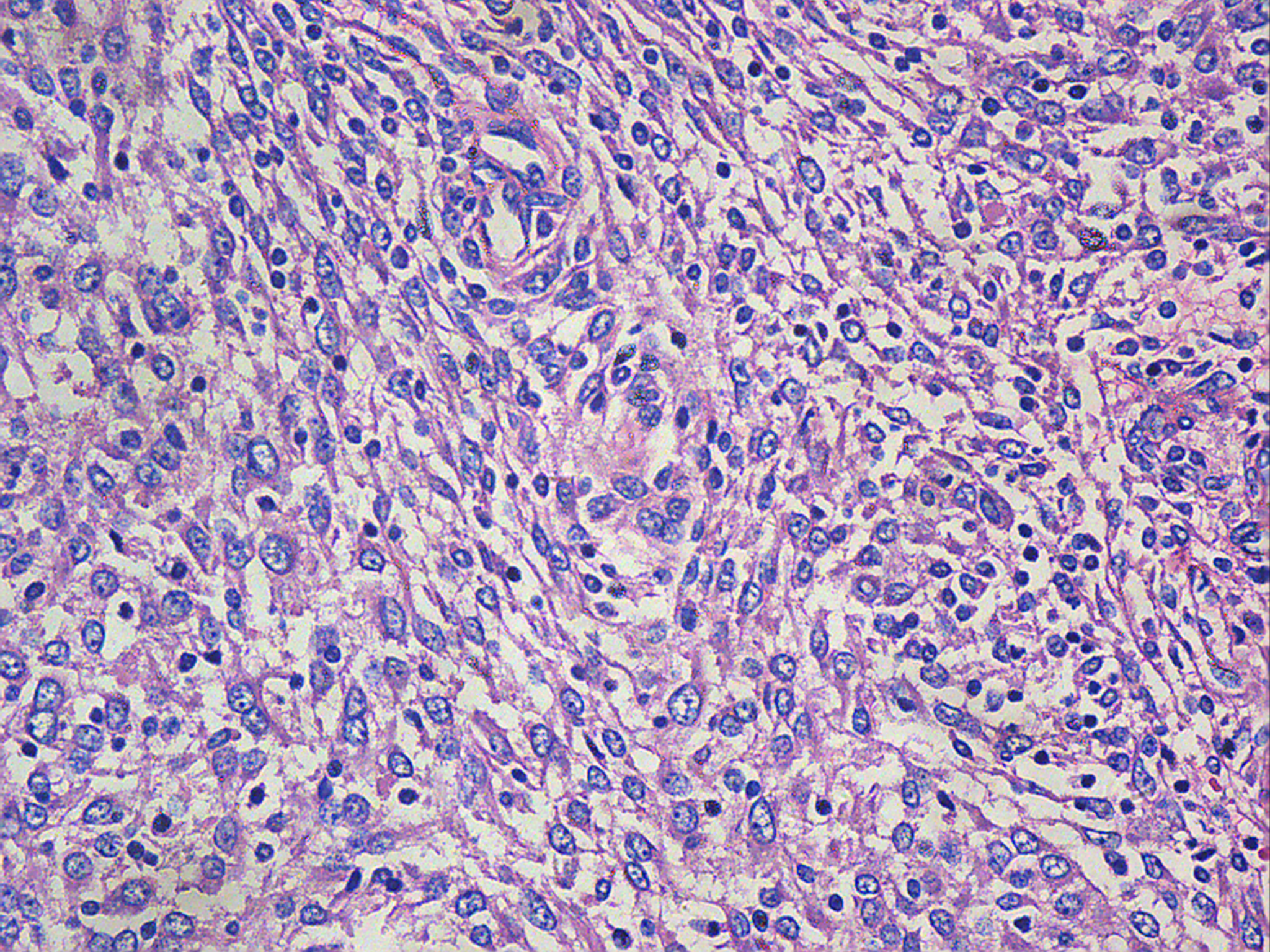

Supplement: S9 Fig — (ZIP) [file pone.0273682.s009.zip › 57.tif]

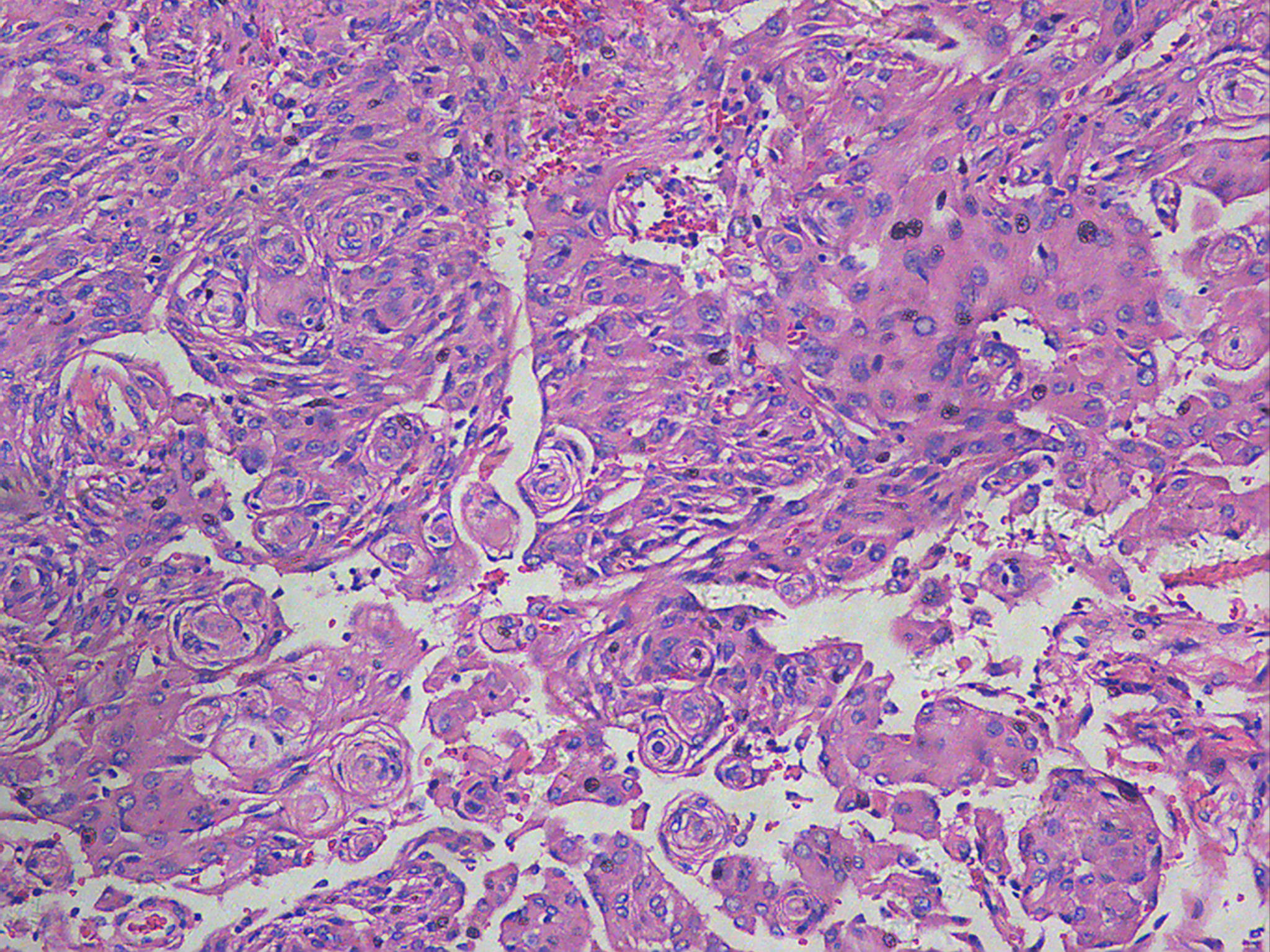

Supplement: S9 Fig — (ZIP) [file pone.0273682.s009.zip › 58.tif]

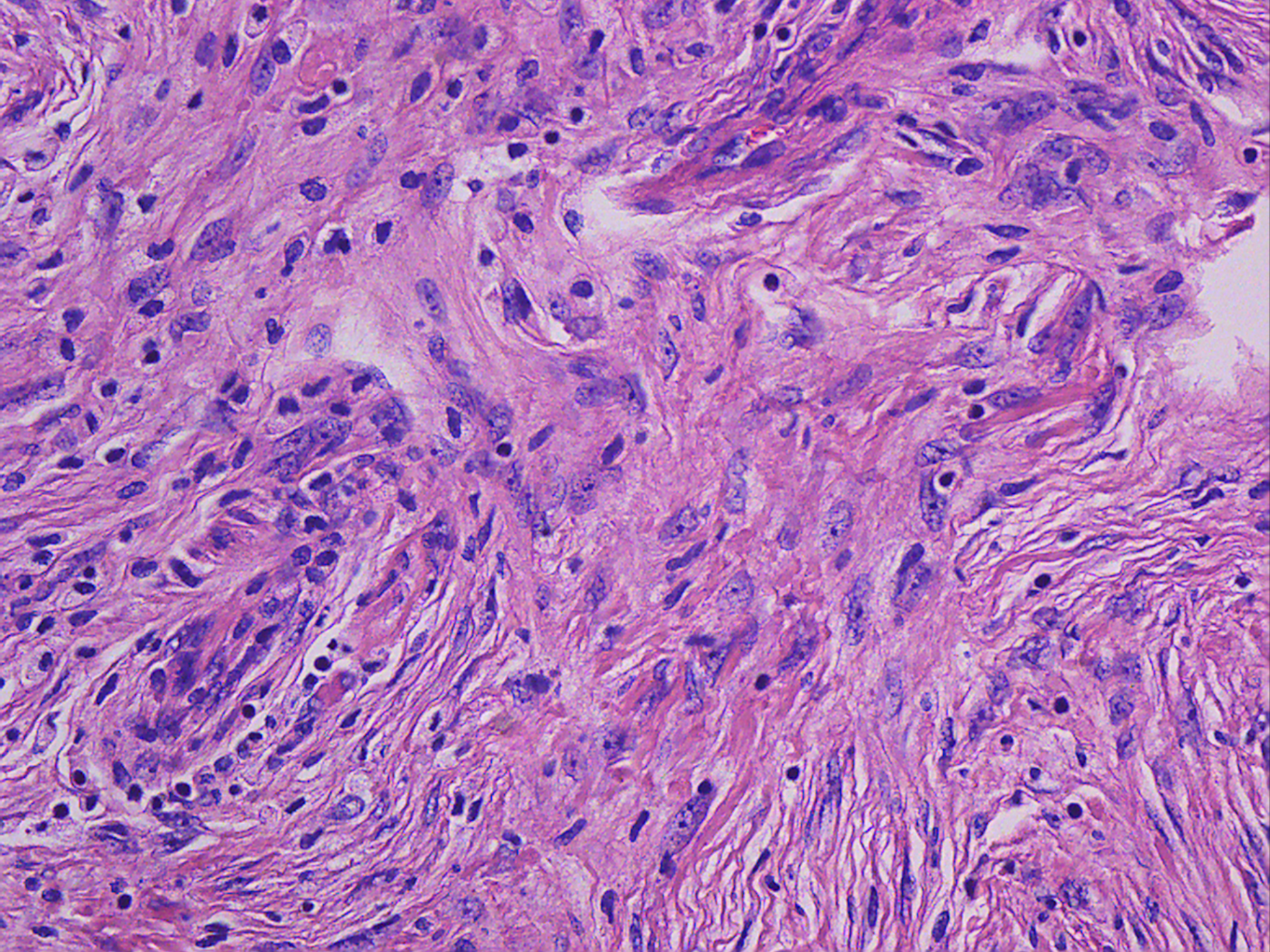

Supplement: S9 Fig — (ZIP) [file pone.0273682.s009.zip › 59.tif]

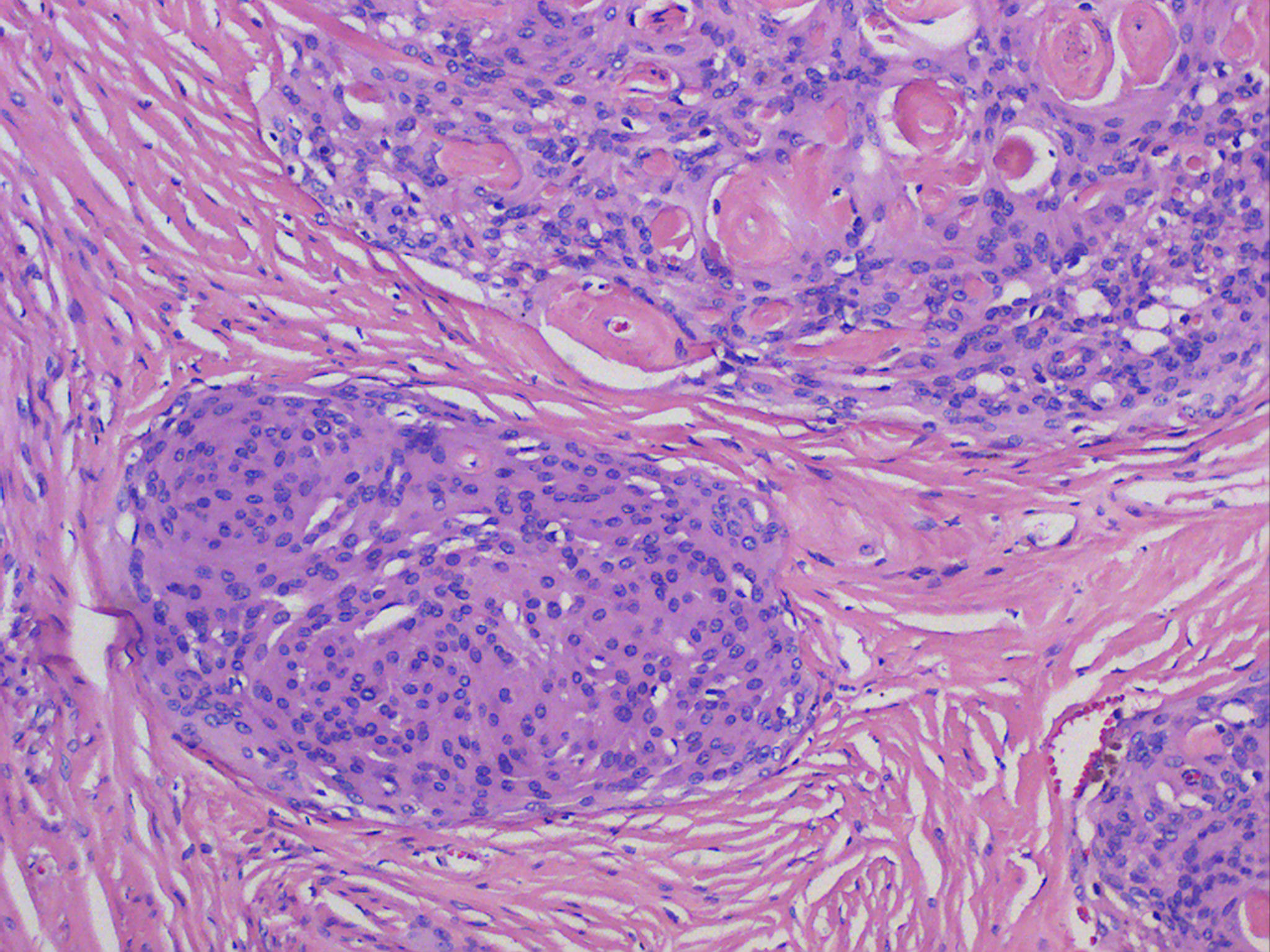

Supplement: S9 Fig — (ZIP) [file pone.0273682.s009.zip › 60.tif]
