# Supplementary material for: Nuclei segmentation of HE stained histopathological images based on feature global delivery connection network
Source: PLoS One. 2022 Sep 15;17(9):e0273682. doi: 10.1371/journal.pone.0273682 (PMC9477331; doi:10.1371/journal.pone.0273682)
Supplement: S1 File — (PDF) [file pone.0273682.s010.pdf]

✓ ㉔

福建医科大学附属协和医院科研项目伦理审查表

科研项目伦理审批编号：2019KJTYL024

研究项目名称	基于多模态功能磁共振成像及影像组学预测脑膜瘤生物学行为的研究		
研究项目负责人	林霖	项目承担科室	放射科

研究内容概述：

脑膜瘤的生物学行为与患者诊疗方案选择和预后评价息息相关，然而常规影像学在术前难以准确评估脑膜瘤的生物学行为。能否通过对功能磁共振成像，得到脑膜瘤的生物学信息是亟需解决的问题。本研究拟运用功能磁共振对脑膜瘤进行多模态弥散成像和灌注成像，结合基于影像组学的分析方法，通过术前精准定量肿瘤功能影像学指标，预测术后肿瘤病理分级分型及免疫组化指标，从而在体内无创性了解包括肿瘤的血流动力学、肿瘤的增殖活性等生物学行为，建立一项直观无创的功能影像诊断模型，为实现个体化治疗提供指导，提高患者的生活质量和预后。

本研究仅涉及患者病史数据采集和废弃组织样本使用，研究过程不干预患者的治疗，并征得患者或患者家属的知情同意，承诺保护患者隐私。

项目负责人（签章）：林霖

2019年 1 月 28日

审查意见：

同意我院 放射 科 林霖 医师在遵循 GCP 原则和国家有关法律要求的基础上申报该项研究。

伦理委员会盖章：

2019 年 1 月 31 日
